# Supplementary material for: Minute amounts of helicase-deficient truncated RECQL4 are sufficient for DNA replication
Source: EMBO Rep. 2026 Mar 10;27(7):1759–88. doi: 10.1038/s44319-026-00727-2 (PMC13076768; doi:10.1038/s44319-026-00727-2)
Supplement: Supplementary file 1 — Appendix [file 44319_2026_727_MOESM1_ESM.pdf]

Appendix to: **Minute amounts of helicase-deficient truncated RECQL4 are sufficient for DNA replication.**

Table of Contents

Appendix Table S1. Genotyping primers (all 5'-3') 2

Appendix Table S2. sgRNA sequences used in this study 3

Appendix Figure S1. Loss of function rescue screen metrics. 5-6

Appendix Figure S2. Additional validation that loss of *Klhdc3* rescues the proliferative defect of both *Recql4* point mutant and deficient myeloid cells. 7-8

Appendix Figure S3. Validation of *Klhdc3* targeting by sgRNA. 9-10

Appendix Figure S4. Synthetic lethal interactions with RECQL4 in human RPE-1 cells. 11-12

Appendix Table S1. Genotyping primers (all 5'-3')

Gene	Primer	Primer (all 5'-3')	Product size	Ref
Recql4	WT	ACAGCAACAGAACAGCAACTACG	WT = 165bp	(Smeets *et al.*, 2014)
	floxed	CACTCTAGAAGAGGGAGTCAGATGG	floxed =325bp	
	deleted	CGCGCGAAAGCTGAGGAGTT	deleted =256bp	
**Klhdc3**	WT	GCATGGTGGAACAAGAGACTTT	WT = 306bp	
	floxed	GCCAAGAGCAGAGAGATGGG	floxed =386bp	
	deleted	CTGTGGCCCAGGGGATGTTA	recombined = 552bp	
			germ-line deleted =451bp	
***R26*-CreER**	Primer 1	AAAGTCGCTCTGAGTTGTTAT	WT = 650bp	RRID:IMSR_JAX:008463
	Primer 2	CCTGATCCTGGCAATTTCG	KI =825bp	
	Primer 3	GGAGCGGGAGAAATGGATATG		
Recql4^{R347*}	Primer 1	GAAGGTGACCAAGTTCATGCTAAAGCGTTTGTTTTTCATGTTGAGTCG	KASP assay	(Castillo-Tandazo *et al.*, 2019)
	Primer 2	GAAGGTCGGAGTCAACGGATTCAAAGCGTTTGTTTTTCATGTTGAGTCA		
	Reverse primer	GCTTCCCTAGACAGAGGGAACTATA		

Recql4^{G522Efs}

The presence of the G522Efs (Castillo-Tandazo *et al.*, 2019) and R347* mutations was determined by KASP (competitive allele specific PCR) technology (LGC) with custom designed (G522Efs) sequences according to manufacturer instructions.

Appendix Table S2. sgRNA sequences used in this study

Name	Sequence (5'-3')	Notes
sg*Cd44* F	CACCGAAGGAAATGTGGTAATTCCG	
sg*Cd44* R	AAACCGGAATTACCACATTTCCTTC	
sg*Klhdc3* BRIE_2 F	CACCGAGTCTTTGATACCAGAACGG	validation_1 F
sg*Klhdc3* BRIE_2 R	AAACCCGTTCTGGTATCAAAGACTC	validation_1 R
sg*Klhdc3* BRIE_3 F	CACCGGCTAGGGCAATCCTGCACGT	validation_2 F
sg*Klhdc3* BRIE_3 F	AAACACGTGCAGGATTGCCCTAGCC	validation_2 R
sg*Klhdc3* validation_3 F	CACCGAAAGGCGTAGAGTACGTTGC	
sg*Klhdc3* validation_3 R	AAACGCAACGTACTCTACGCCTTTC	
sg*Klhdc3* validation_4 F	CACCGGGCACCGAGTATATTCCTTC	
sg*Klhdc3* validation_4 R	AAACGAAGGAATATACTCGGTGCCC	
m*Klhdc3* del PCR 1F	GCAGGGTGAACCATGCTG	T7E1 assay/sequencing
m*Klhdc3* del PCR 1R	CCAGGTCCAAGGTGTCATTT	
m*Klhdc3* del PCR 2F	CCACCCTTGGCTGGAATAG	T7E1 assay/sequencing
m*Klhdc3* del PCR 2R	CAGACACTCACAGGCTGAAT	
m*Klhdc3* RT-qPRC 1F	TTGGCTACAATGGAGAGCTG	
m*Klhdc3* RT-qPCR 1R	CAGGTAAAGGACCCAGGATTAAA	
sg*KLHDC3*_1 F	CACCGACGGTGGACAGTGCACCTGG	
sg*KLHDC3*_1 R	AAACCCAGGTGCACTGTCCACCGTC	
sg*KLHDC3*_2 F	CACCGGGCGGGCGGAATGACACCGA	
sg*KLHDC3*_2 R	AAACTCGGTGTCATTCCGCCCGCCC	
h*KLHDC3* del PCR F	GGAGGCAAAGGCTGGTTC	T7E1 assay/sequencing
h*KLHDC3* del PCR R	TTGGGCAACTGAGGCAAC	
sgControl_1 F	CACCGGGCAGAAGGAACACAGGCTC	
sgControl_1R	AAACGAGCCTGTGTTCCTTCTGCCC	
sg*Zfp36l2*_1 F	CACCGTCATCCACAACGCGGACGAG	negative control
sg*Zfp36l2*_1 R	AAACCTCGTCCGCGTTGTGGATGAC	
sg*Cd81*_1 F	CACCGGCAACCACAGAGCTACACCT	negative control
sg*Cd81*_1 R	AAACAGGTGTAGCTCTGTGGTTGCC	
sg*Recql4* BRIE_1 F	CACCGGACACCTCTCTAACCAACCA	
sg*Recql4* BRIE_1 R	AAACTGGTTGGTTAGAGAGGTGTCC	
sg*Recql4* BRIE_2 F	CACCGCAATCTGAAAAACACAACAC	
sg*Recql4* BRIE_2 R	AAACGTGTTGTGTTTTTCAGATTGC	
sg*Recql4* BRIE_3 F	CACCGGGGGCTCTGTGACAAAACCT	
sg*Recql4* BRIE_3 R	AAACAGGTTTTGTCACAGAGCCCCC	
sg*Recql4* BRIE_4 F	CACCGAGAGACTTTCCAGCACCCGT	
sg*Recql4* BRIE_4 R	AAACACGGGTGCTGGAAAGTCTCTC	
sg*Recql4* BROAD_1 F	CACCGGTACAACAGGCCTTCATGCG	

sg*Recql4* BROAD_1 R	AAACCGCATGAAGGCCTGTTGTACC	
sg*Ccnf* BRIE_2 F	CACCGGTAACTGACACTCCGCTCGG	
sg*Ccnf* BRIE_2 R	AAACCCGAGCGGAGTGTCAGTTACC	
sg*Ccnf* BRIE_4 F	CACCGAGTCTTTGGGTGCATCATCG	
sg*Ccnf* BRIE_4 R	AAACCGATGATGCACCCAAAGACTC	
sg*Ccnf* BROAD_1 F	CACCGATGGCTGAGAGACTGAATAC	
sg*Ccnf* BROAD_1 R	AAACGTATTCAGTCTCTCAGCCATC	
m*Ccnf* del PCR 1F	AGAGCTTTGCAGTGTGGTAG	T7E1 assay/sequencing
m*Ccnf* del PCR 1R	GCTTCTAGAATGCTAGGTGAGAG	
m*Ccnf* del PCR 2F	AACACTCTTGTGCCTGATCTT	T7E1 assay/sequencing
m*Ccnf* del PCR 2R	GACAGAGTAGACTCACCGTTTG	
m*Mcm10*_3 1F	CACCGCATCAATTAAACAGCCTCCA	
m*Mcm10*_3 1R	AAACTGGAGGCTGTTTAATTGATGC	
m*Mcm10*_4 1F	CACCGATGTTACAGCTACTGACCTG	
m*Mcm10*_4 1R	AAACCAGGTCAGTAGCTGTAACATC	
m*Mcm10* sg3 del PCR 1F	CAAACCACACACACACACAC	
m*Mcm10* sg3 del PCR 1R	GTAAGGAAGACCCTGAATGC	Amplicon: 1141bp
m*Mcm10* sg4 del PCR 1F	GGTTAGGTTGGCCATGTAACTA	
m*Mcm10* sg4 del PCR 1R	CATAAGAGGTGGAGAATGACTGAG	Amplicon: 911bp

Appendix Figure S1

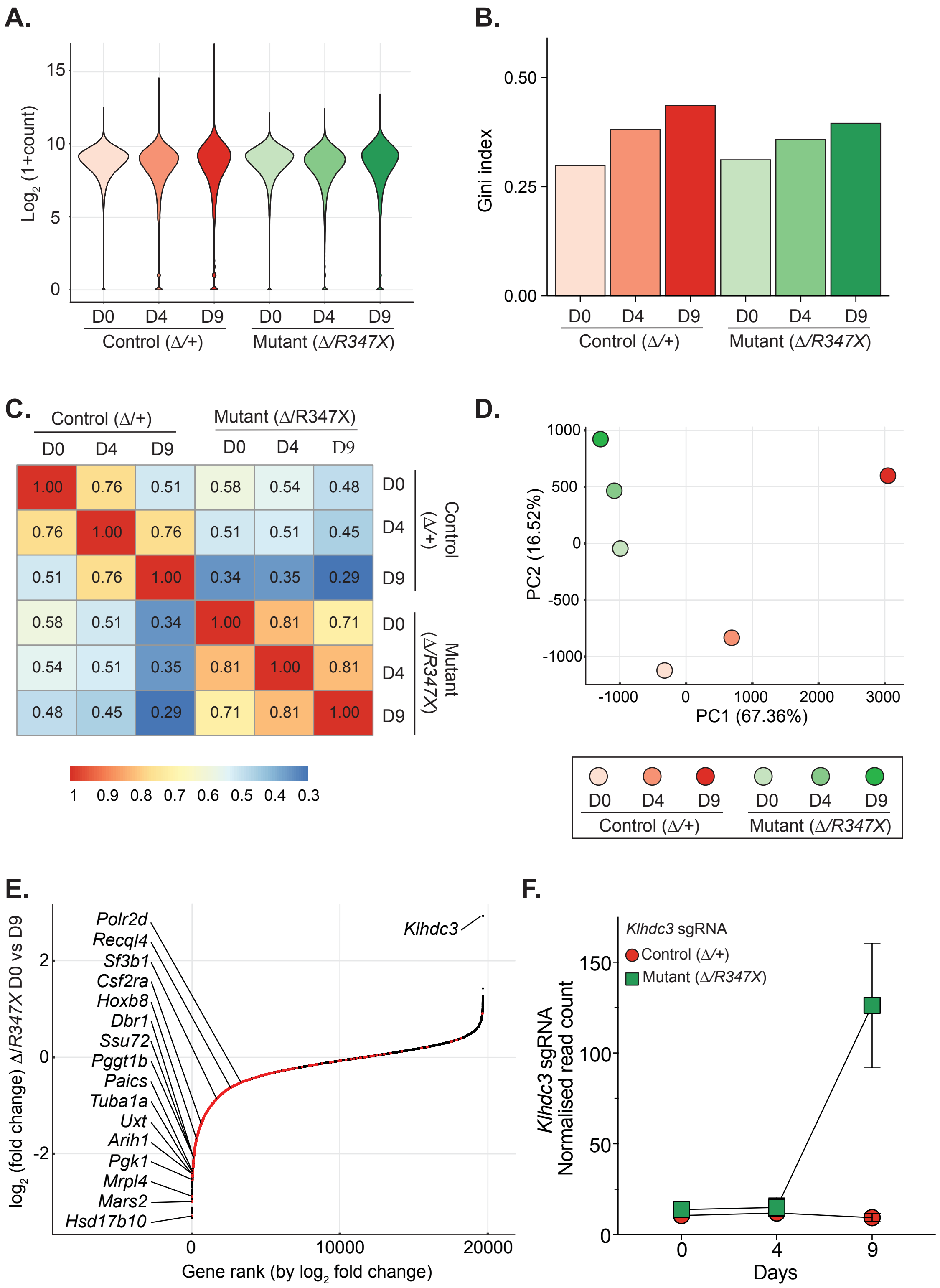

Appendix Figure S1. Loss of function rescue screen metrics.

A. sgRNA count distribution. Violin Plot showing sgRNA frequencies at day 0, 4, and 9 and control (fl/+) and mutant (fl/R347X) cell lines
B. Gini index computed from normalised sgRNA counts for all cell line replicates.
C. Heatmap showing sgRNA-level correlation between timepoints of control and mutant cell lines.
D. Principle component analysis (PCA) of sgRNA-sequencing results from day 0, 4, and 9 of control and mutant cell lines
E. Essential gene depletion. sgRNAs of R347 D9 vs D0 were ranked by log2 fold change, showing loss of representation of known and predicted essential genes.
F. Normalised sgRNA counts for the four *Klhdc3* guides in the library.

Appendix Figure S2

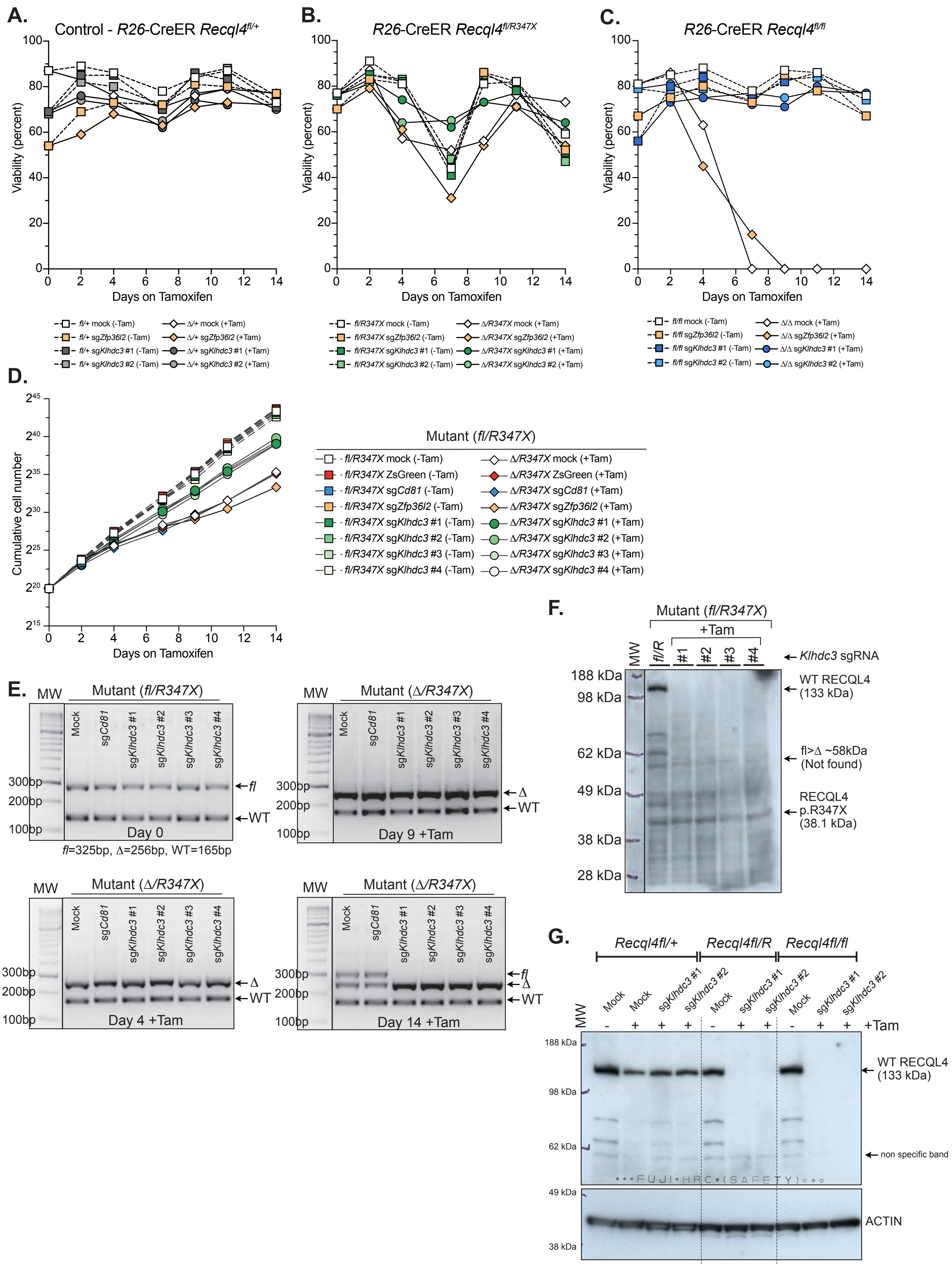

Appendix Figure S2. Additional validation that loss of *Klhdc3* rescues the proliferative defect of both *Recql4* point mutant and deficient myeloid cells.

A. Effect of loss of *Klhdc3* on control cells (*R26*-CreER *Recql4*$^{fl/+}$; become Δ/+ cell following tamoxifen treatment applied at Day 0) viability (related to Figure 1C).

B. Effect of loss of *Klhdc3* on *Recql4* p.R347X only expressing cells (*R26*-CreER *Recql4*$^{fl/R347X}$; become *Δ/R347X* cells following tamoxifen treatment applied at Day 0) viability (related to Figure 1D).

C. Effect of loss of *Klhdc3* on *Recql4* deficient cells (*R26*-CreER *Recql4*$^{fl/fl}$; become Δ/Δ cells following tamoxifen treatment applied at Day 0) viability (related to Figure 1E).

D. Proliferation assay showing that four sgRNAs against *Klhdc3* (two from the BRIE library and two new guides) rescue *Recql4*^{R347X} point mutant cells (circles). Results were compared to non-tamoxifen treated cells (squares), and sgRNA targeting *Cd81* (a non-essential cell surface marker), sg*Zfp36l2* (a guide depleted in the mutant but not in the control), ZsGreen (a non-targeting virus control), and a mock (non-infected) control (diamonds).

E. Genomic PCR showing successful recombination and stable deletion of the floxed *Recql4* allele after addition of tamoxifen in *Klhdc3 Recql4* R347X double mutant cells.

F. Western blot of full-length WT and truncated R347X mutant RECQL4 protein in the non-tamoxifen treated control and tamoxifen-treated *Klhdc3* sgRNA mutant cells (Day 9 Post-Tam).

G. Western blot of control and tamoxifen treated *Recql4*$^{fl/+}$ (heterozygous control after tamoxifen treatment), *Recql4*$^{R347X/fl}$ and *Recql4*$^{fl/fl}$ myeloid cells probing for RECQL4 or ACTIN as indicated. Treatment as indicated (Mock = non-infected or sg*Klhdc3* #1 or #2).

Appendix Figure S3

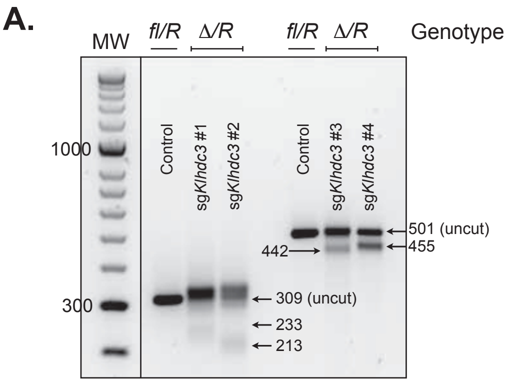

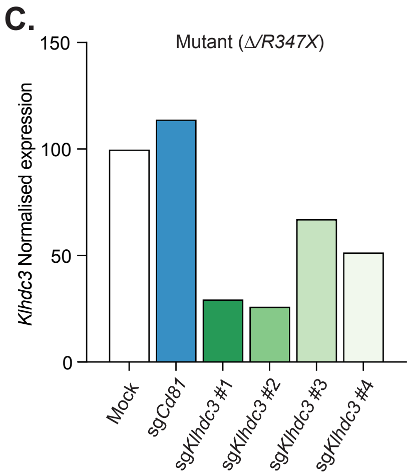

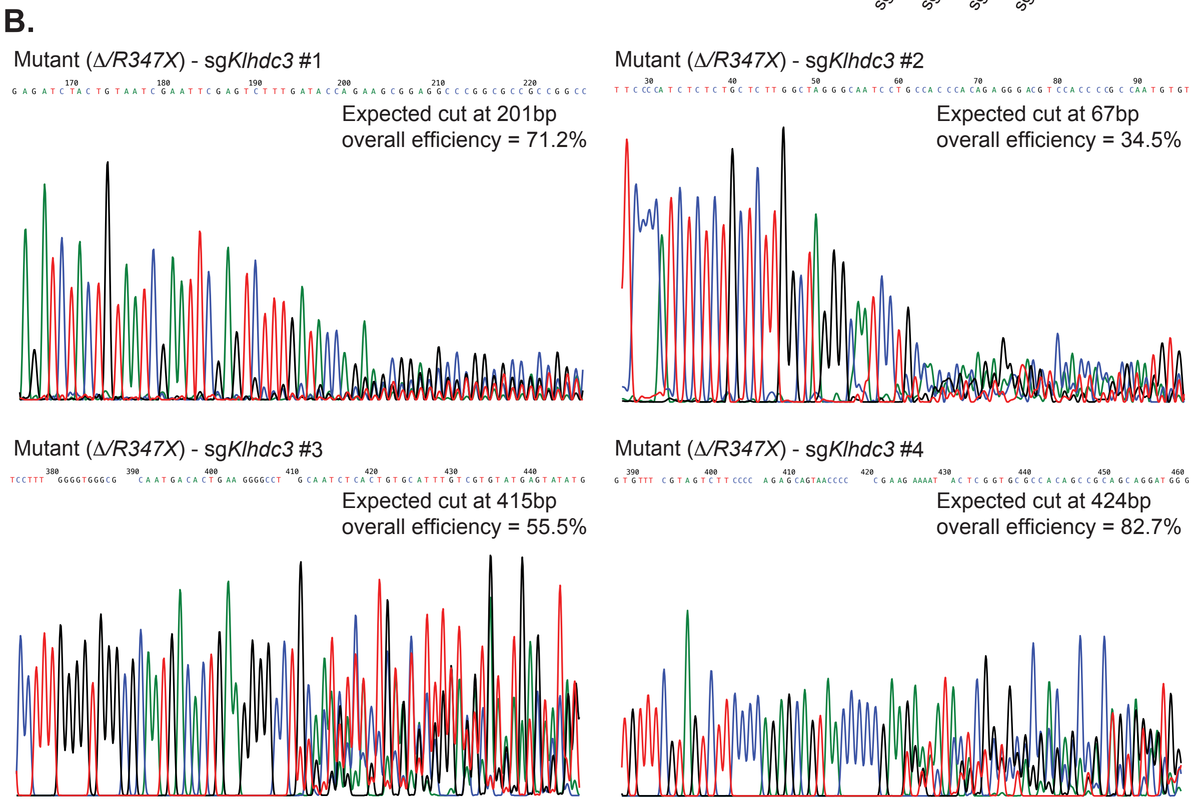

Appendix Figure S3. Validation of *Klhdc3* targeting by sgRNA.

A. T7 endonuclease digestion of heteroduplex PCR products amplified from the genomic DNA surrounding the sgRNA target sites, showing cleavage of mismatched DNA at sites of indels ≥2 bases in a mixed population of *Klhdc3* gRNA targeted mutant (R347X) cells (Day 9 Post-Tam). The sizes of the predicted cleavage products are labelled in the figure.

B. TIDE Quantitative Sanger sequence trace analysis of amplified genomic DNA regions surrounding the expected break site of individual *Klhdc3* sgRNAs in a mixed population of mutant (R347X) cells (Day 9 Post-Tam). All cells were subsequently cloned and sequenced to ensure homozygous mutations were present.

C. Quantitative real-time RT-PCR analysis of *Klhdc3* RNA expression in mock and tamoxifen-treated *sgKlhdc3 Recql4 R347X* double mutant cells (Day 9 Post-Tam). (Expression relative to *Gapdh* and normalised to mock).

Appendix Figure S4

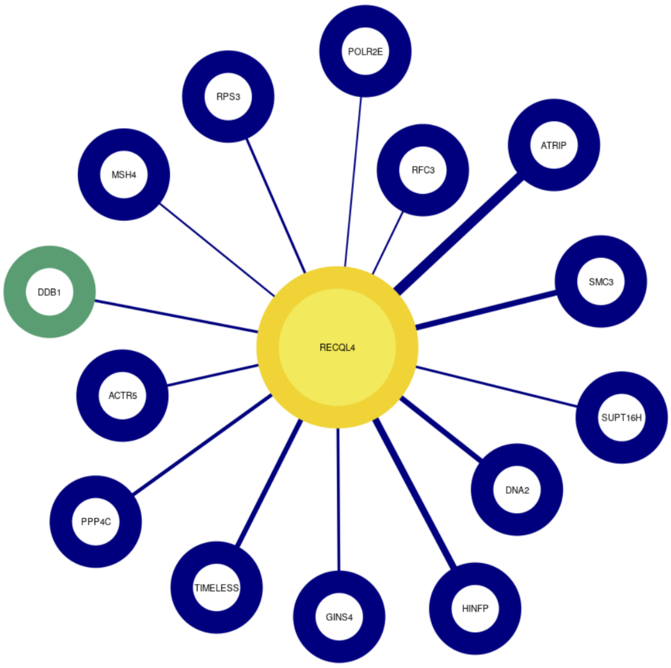

Gene A	Gene B	Pair	GEMINI sensitive	Cell line	Below cutoff?
RECQL4	ACTR5	ACTR5;RECQL4	-1.1105	RPE-1	Yes
RECQL4	ATRIP	ATRIP;RECQL4	-1.9635	RPE-1	Yes
RECQL4	DDB1	DDB1;RECQL4	-1.1188	RPE-1	Yes
RECQL4	DNA2	DNA2;RECQL4	-1.4275	RPE-1	Yes
RECQL4	GINS4	GINS4;RECQL4	-1.1264	RPE-1	Yes
RECQL4	HINFP	HINFP;RECQL4	-1.5895	RPE-1	Yes
RECQL4	MSH4	MSH4;RECQL4	-1.0257	RPE-1	Yes
RECQL4	POLR2E	POLR2E;RECQL4	-1.0015	RPE-1	Yes
RECQL4	PPP4C	PPP4C;RECQL4	-1.2235	RPE-1	Yes
RECQL4	RFC3	RECQL4;RFC3	-1.0218	RPE-1	Yes
RECQL4	RPS3	RECQL4;RPS3	-1.0959	RPE-1	Yes
RECQL4	SMC3	RECQL4;SMC3	-1.4712	RPE-1	Yes
RECQL4	SUPT16H	RECQL4;SUPT16H	-1.0922	RPE-1	Yes
RECQL4	TIMELESS	RECQL4;TIMELESS	-1.38	RPE-1	Yes

https://spidrweb.org/app/spidrweb/
Accessed30/05/2025
Gene of interest search term: RECQL4
Cell line: RPE-1
Filter GEMINI score: -1

Appendix Figure S4. Synthetic lethal interactions with RECQL4 in human RPE-1 cells.
Data from spidrweb.org/app/spidrweb/; SPIDRweb: Comprehensive Interrogation of Synthetic Lethality in the DNA Damage Response Published in *Nature*: https://doi.org/10.1038/s41586-025-08815-4.
