# Supplementary material for: Minute amounts of helicase-deficient truncated RECQL4 are sufficient for DNA replication
Source: EMBO Rep. 2026 Mar 10;27(7):1759–88. doi: 10.1038/s44319-026-00727-2 (PMC13076768; doi:10.1038/s44319-026-00727-2)
Supplement: Supplementary file 5 — Source data Fig. 1 [file 44319_2026_727_MOESM5_ESM.zip › Figure 1 Source Data/Figure 1 Source data READ ME.docx]

Figure 1 Source data:

Figure 1A. Schematic

Figure 1B. Crispr screen – all raw data in Dataset S1_RECQL4_CB2_Screen_Results excel sheet

Figure 1C-1E. Raw data in Source data excel sheet

Figure 1F. Raw data in Source data

Figure 1G. Raw data in Source data
