# Supplementary figures and images for: Minute amounts of helicase-deficient truncated RECQL4 are sufficient for DNA replication
Source: EMBO Rep. 2026 Mar 10;27(7):1759–88. doi: 10.1038/s44319-026-00727-2 (PMC13076768; doi:10.1038/s44319-026-00727-2)

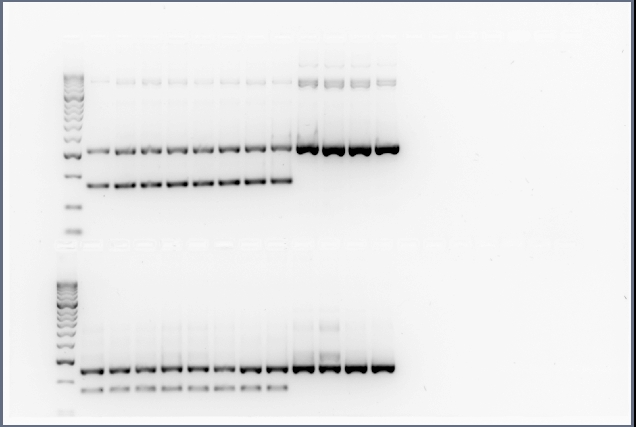

Supplement: Supplementary file 5 — Source data Fig. 1 [file 44319_2026_727_MOESM5_ESM.zip › Figure 1 Source Data/Figure 1F/wcastillo 2020-12-14 15hr 52min Day 0 (top) and 4 (bottom).jpg]

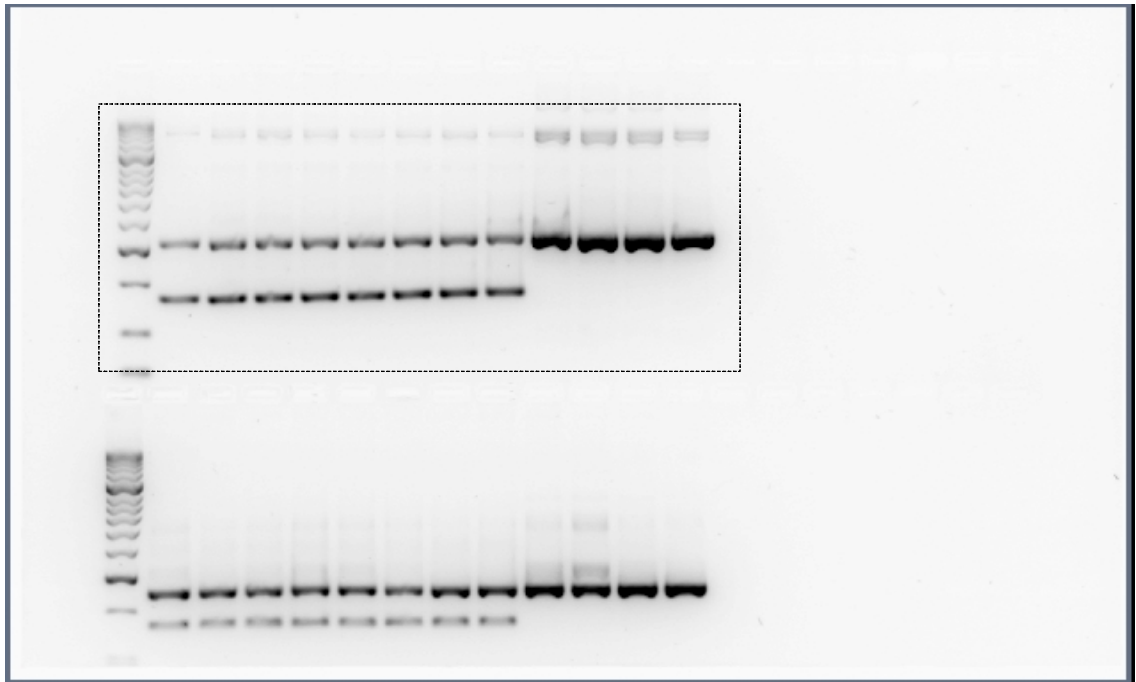

Supplement: Supplementary file 5 — Source data Fig. 1 [file 44319_2026_727_MOESM5_ESM.zip › Figure 1 Source Data/Figure 1F/Figure 1F cropped area indicator.pdf]

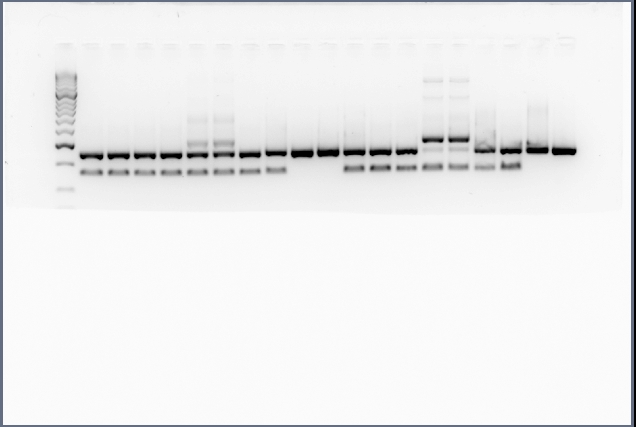

Supplement: Supplementary file 5 — Source data Fig. 1 [file 44319_2026_727_MOESM5_ESM.zip › Figure 1 Source Data/Figure 1G/wcastillo 2020-12-14 15hr 54min.jpg]

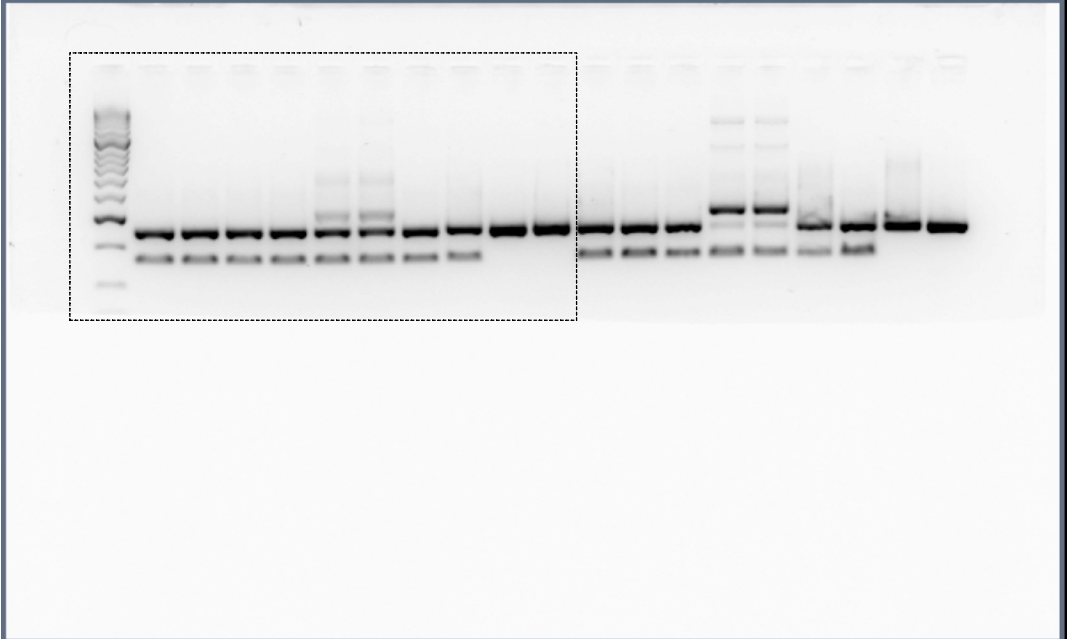

Supplement: Supplementary file 5 — Source data Fig. 1 [file 44319_2026_727_MOESM5_ESM.zip › Figure 1 Source Data/Figure 1G/Figure 1G cropped area indicated.pdf]

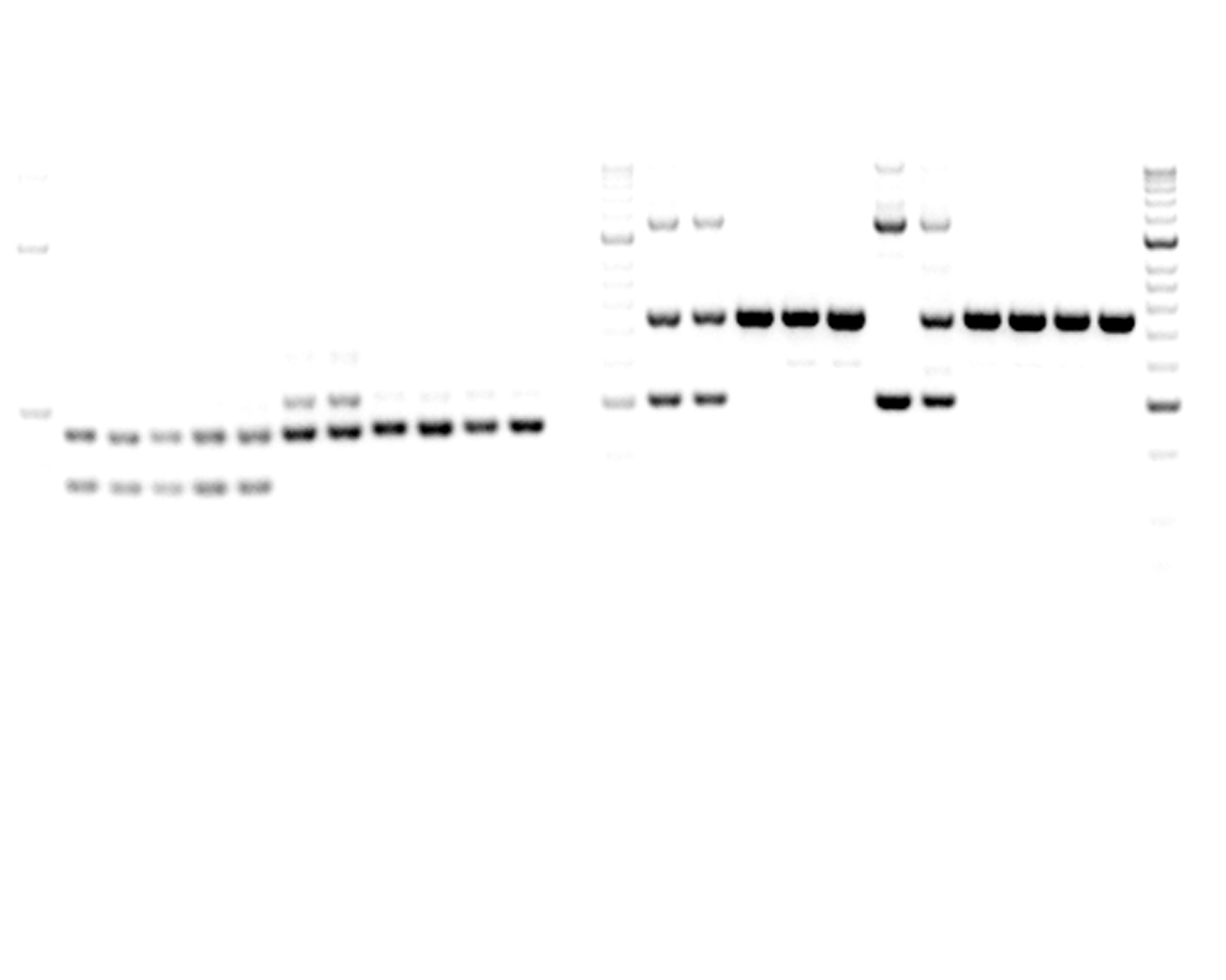

Supplement: Supplementary file 7 — Source data Fig. 3 [file 44319_2026_727_MOESM7_ESM.zip › Figure 3 Source Data/Figure 3D/KLHDC3 GT/NUCLEIC_ACID_24092024_133611_4ms.tif]

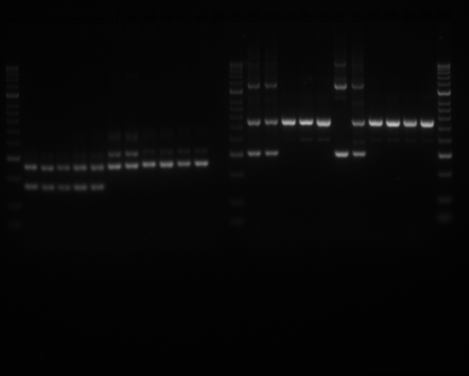

Supplement: Supplementary file 7 — Source data Fig. 3 [file 44319_2026_727_MOESM7_ESM.zip › Figure 3 Source Data/Figure 3D/KLHDC3 GT/NUCLEIC_ACID_24092024_133611_4ms_(Nucleic Acid)_raw.tif]

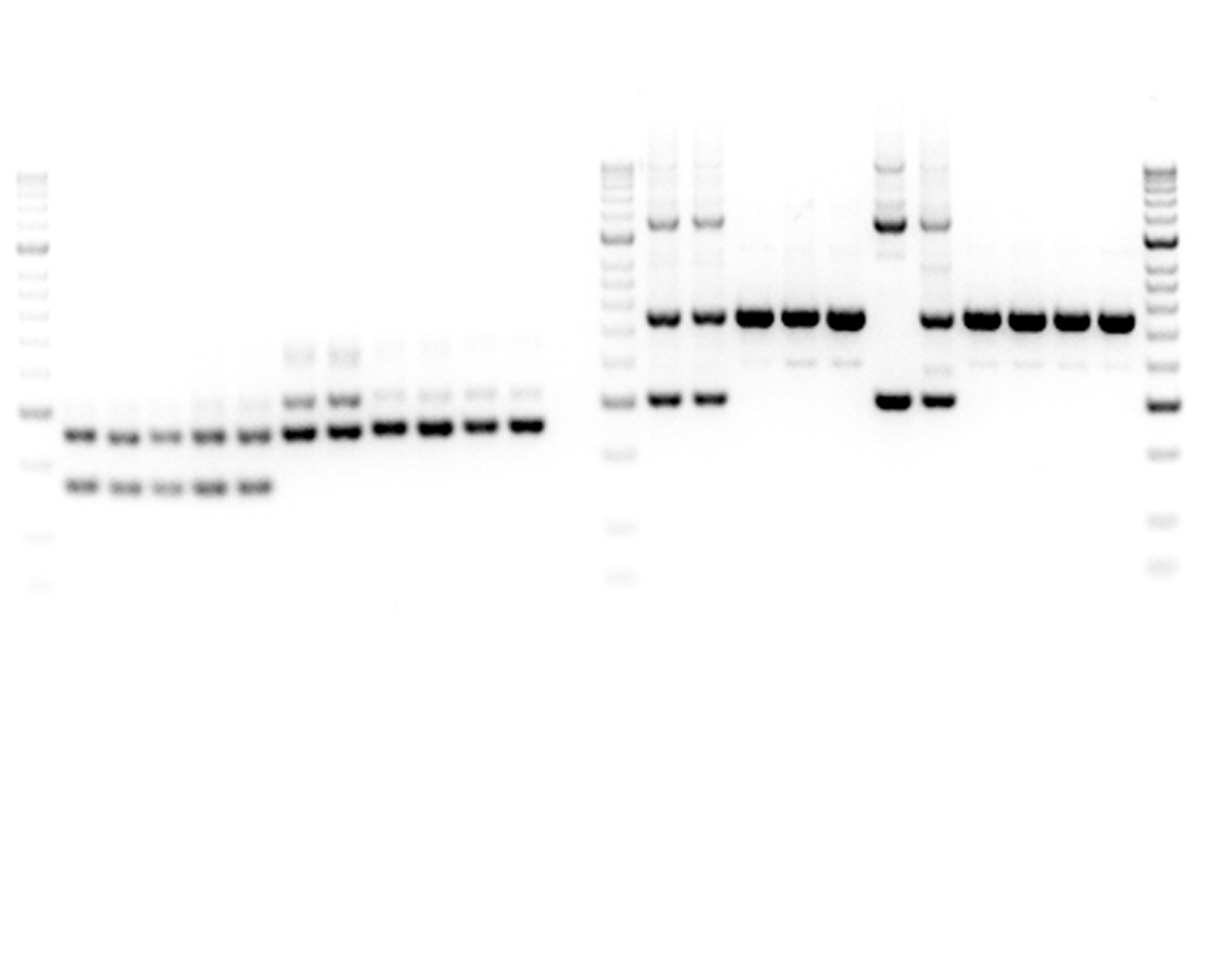

Supplement: Supplementary file 7 — Source data Fig. 3 [file 44319_2026_727_MOESM7_ESM.zip › Figure 3 Source Data/Figure 3D/RECQL4 GT/NUCLEIC_ACID_24092024_133611_4ms.tif]

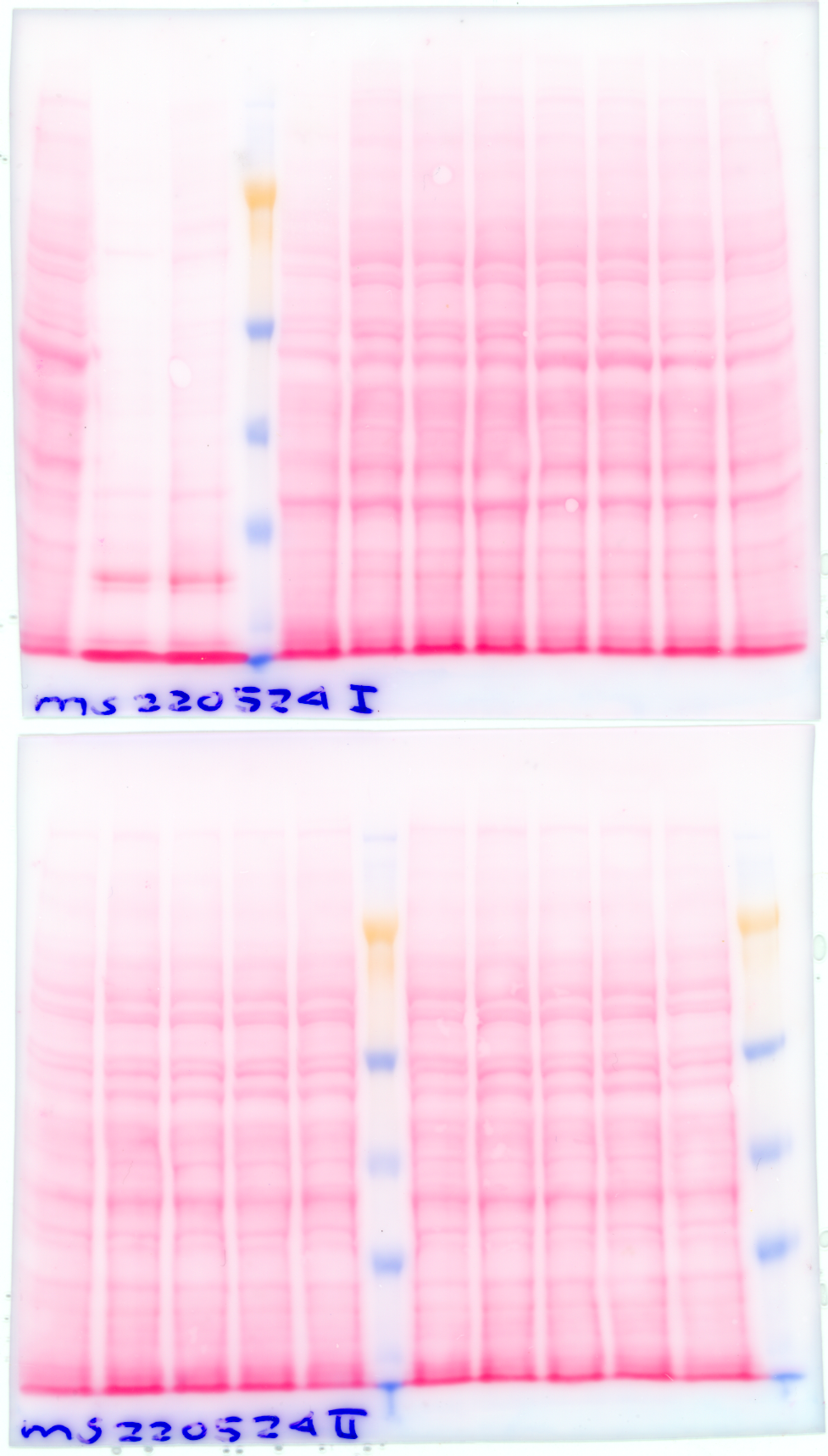

Supplement: Supplementary file 9 — Source data Fig. 5 [file 44319_2026_727_MOESM9_ESM.zip › Figure 5 Source Data/Figure 5C/ms2220524 Myeloid V5 Klhdc3_2.tif]

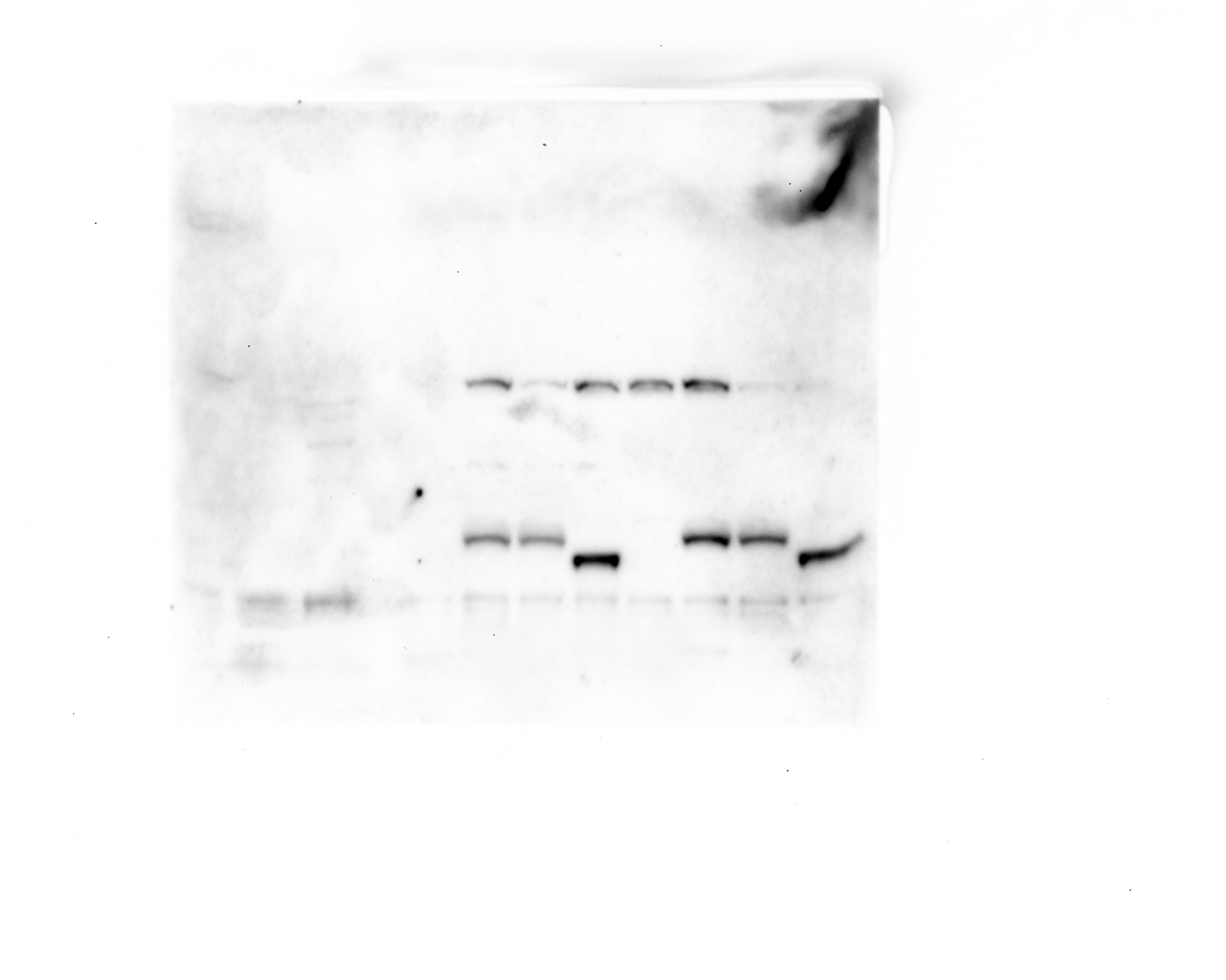

Supplement: Supplementary file 9 — Source data Fig. 5 [file 44319_2026_727_MOESM9_ESM.zip › Figure 5 Source Data/Figure 5C/CHEMI_24052024_093100_25mins_(Chemi).tif]

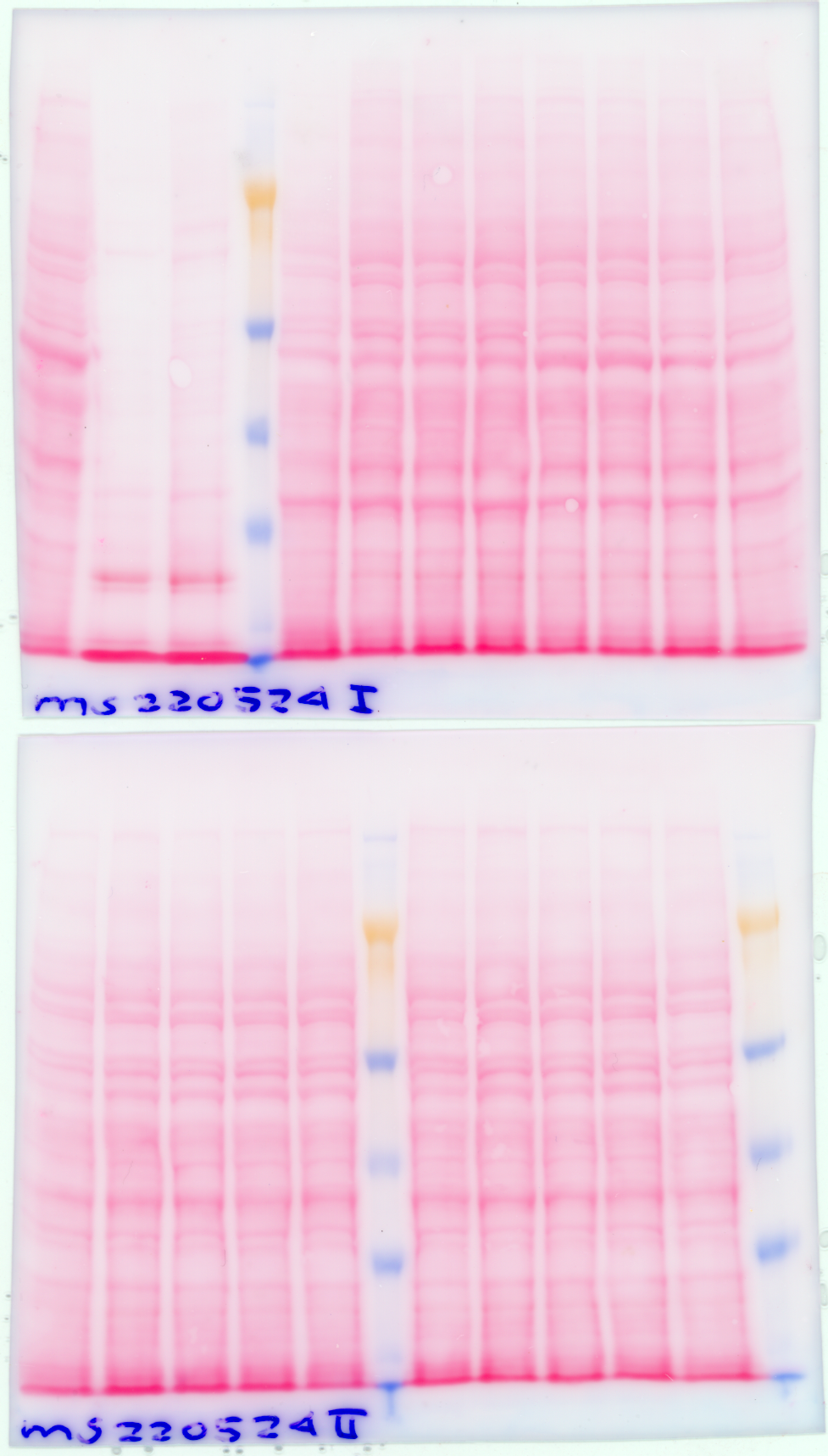

Supplement: Supplementary file 9 — Source data Fig. 5 [file 44319_2026_727_MOESM9_ESM.zip › Figure 5 Source Data/Figure 5C/ms2220524 Myeloid V5 Klhdc3_1.tif]

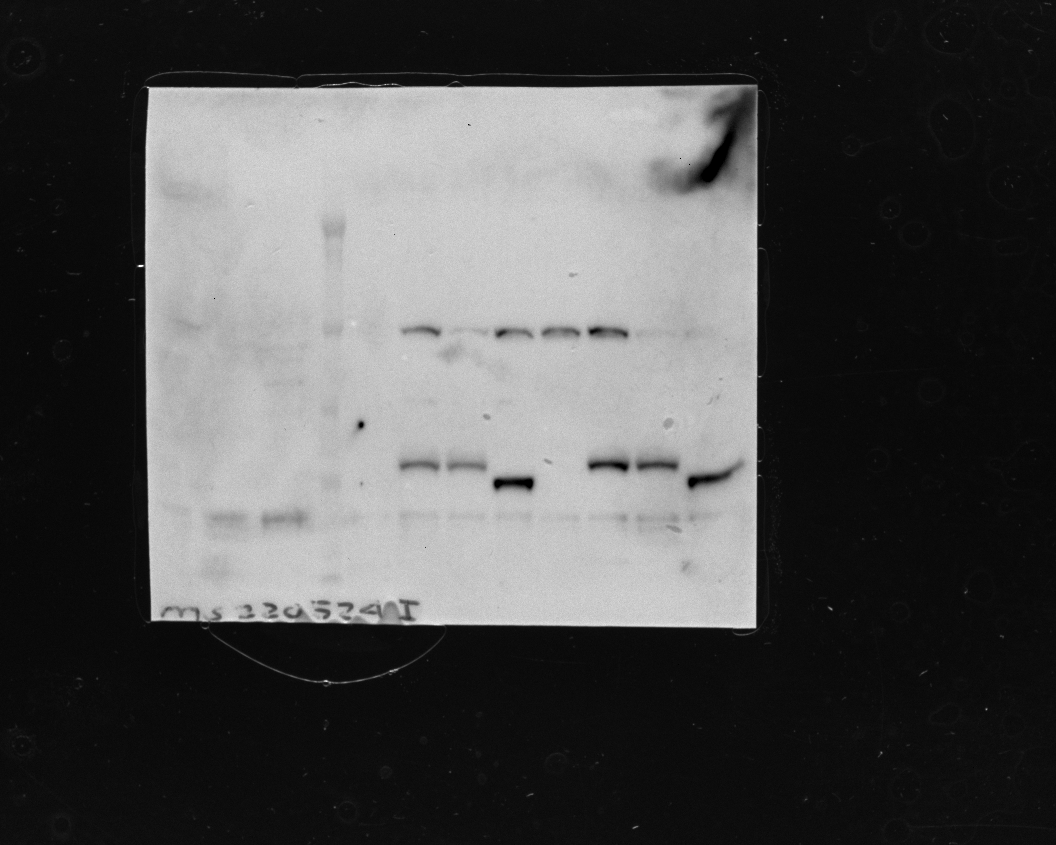

Supplement: Supplementary file 9 — Source data Fig. 5 [file 44319_2026_727_MOESM9_ESM.zip › Figure 5 Source Data/Figure 5C/CHEMI_24052024_093100_25mins_(Chemi) overlay.tif]

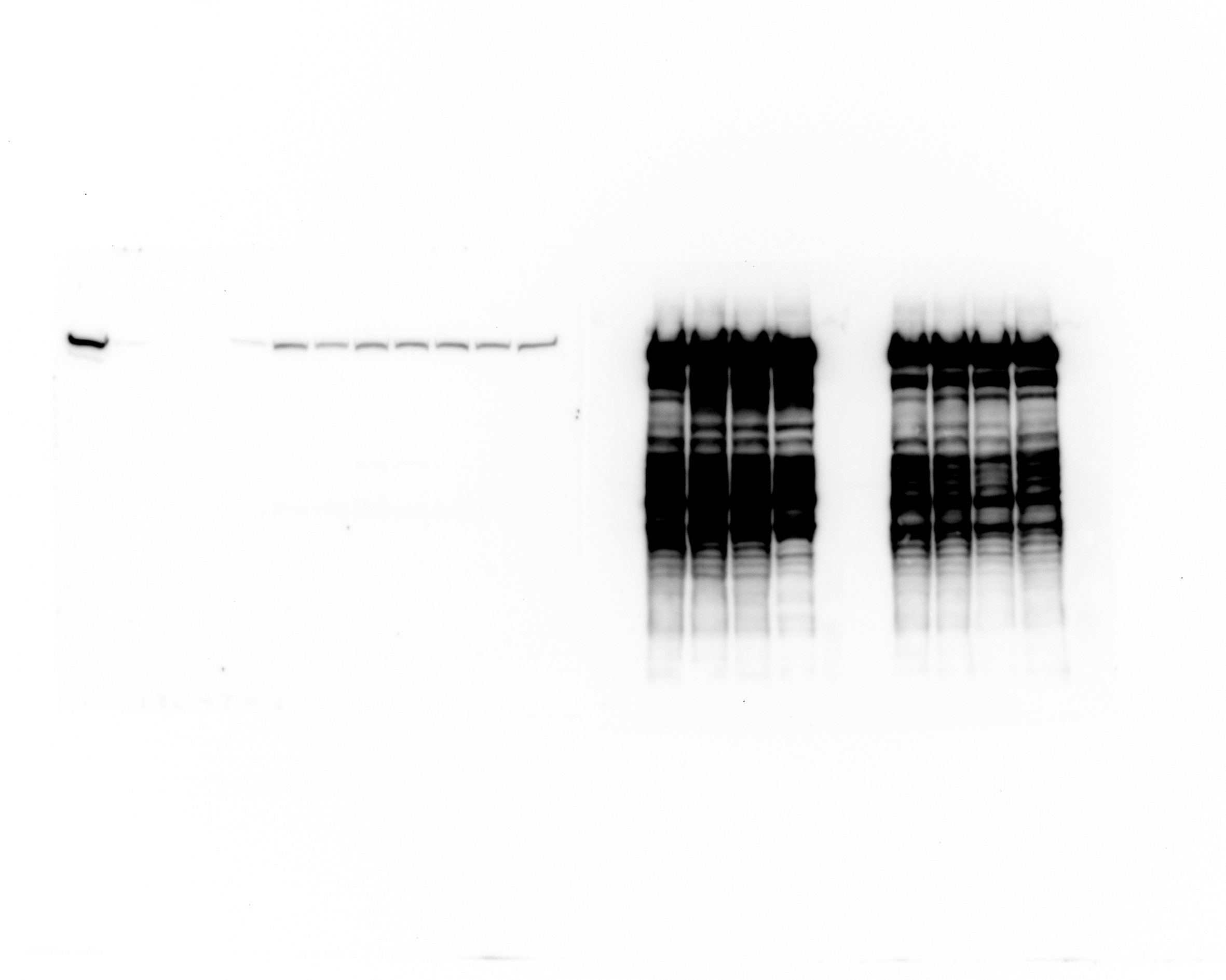

Supplement: Supplementary file 9 — Source data Fig. 5 [file 44319_2026_727_MOESM9_ESM.zip › Figure 5 Source Data/Figure 5C/CHEMI_23052024_091737_5mins_(Chemi).tif]

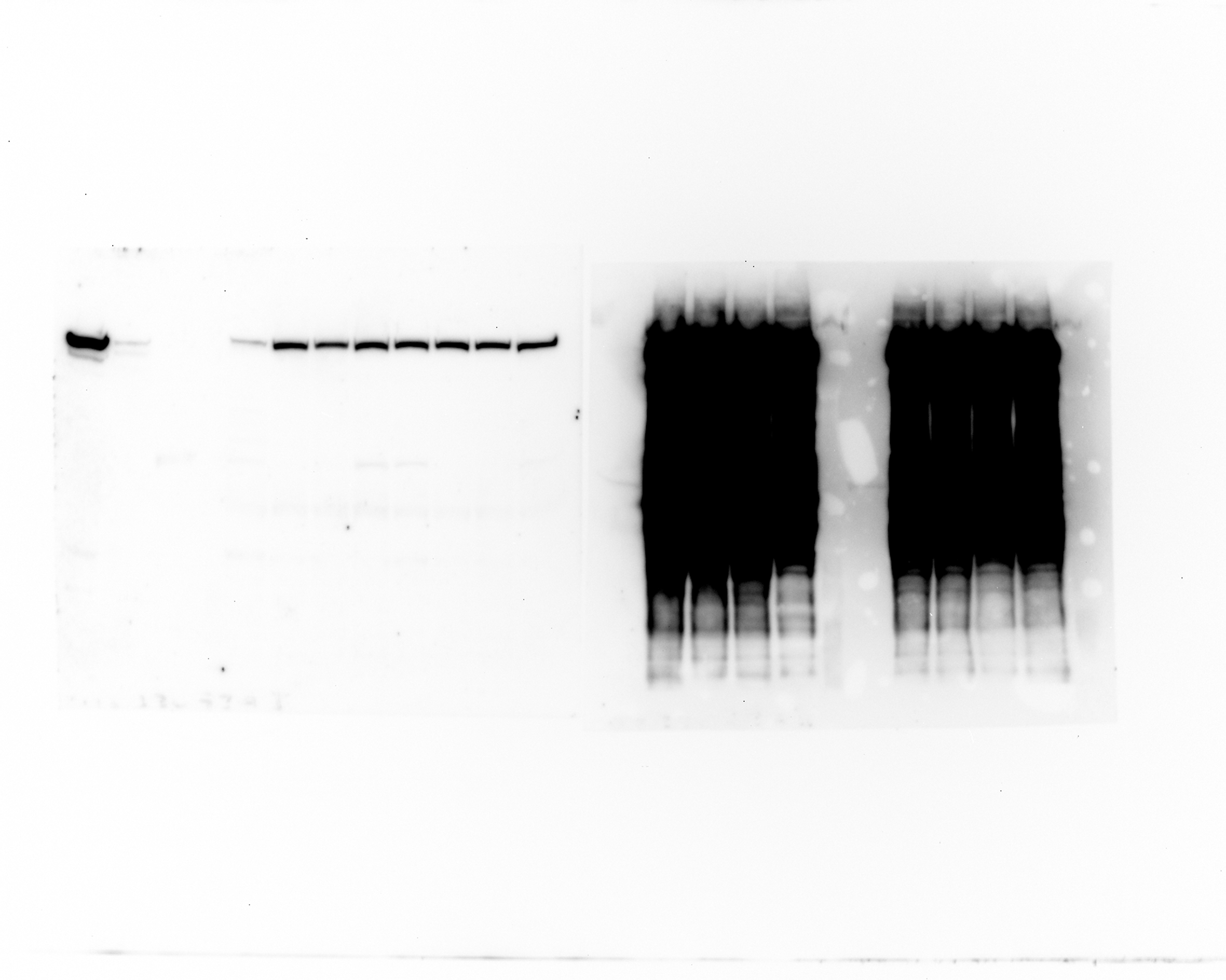

Supplement: Supplementary file 9 — Source data Fig. 5 [file 44319_2026_727_MOESM9_ESM.zip › Figure 5 Source Data/Figure 5C/CHEMI_23052024_093316_20mins_(Chemi).tif]

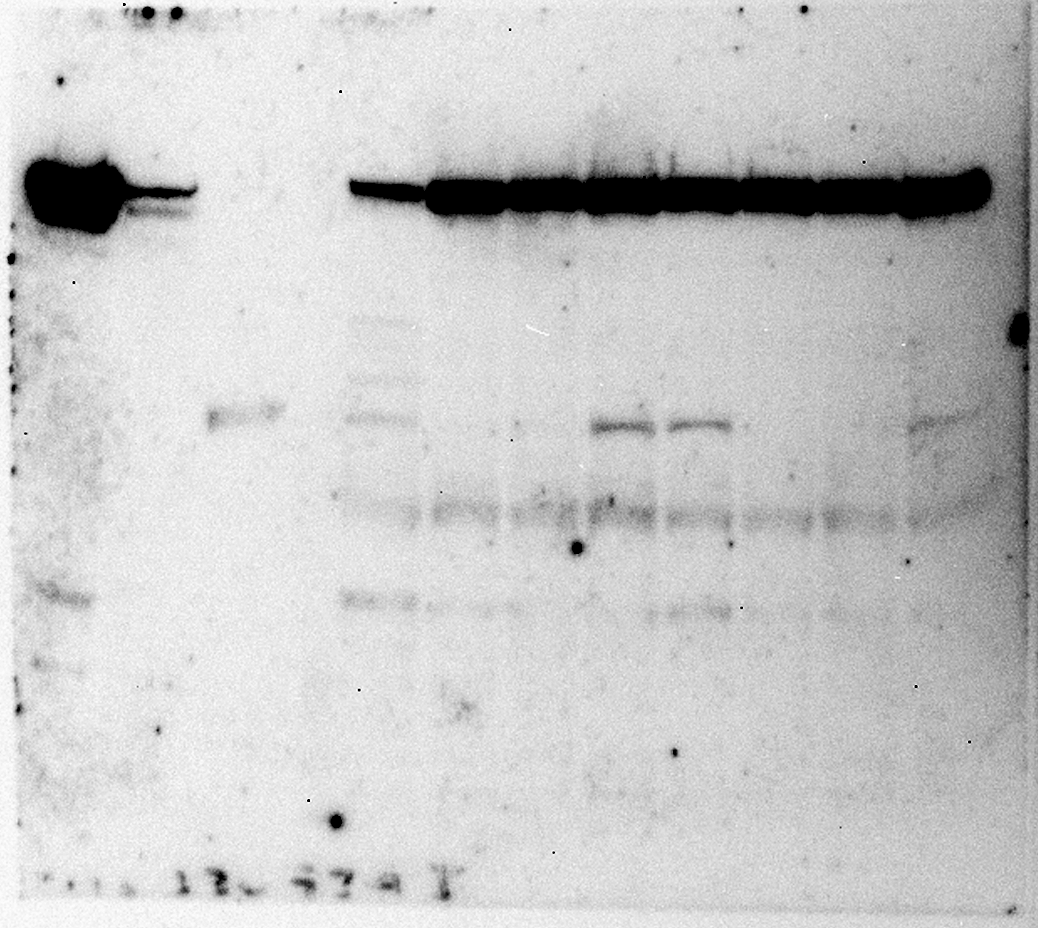

Supplement: Supplementary file 9 — Source data Fig. 5 [file 44319_2026_727_MOESM9_ESM.zip › Figure 5 Source Data/Figure 5C/CHEMI_23052024_093316_20mins_(Chemi) adjusted.tiff]

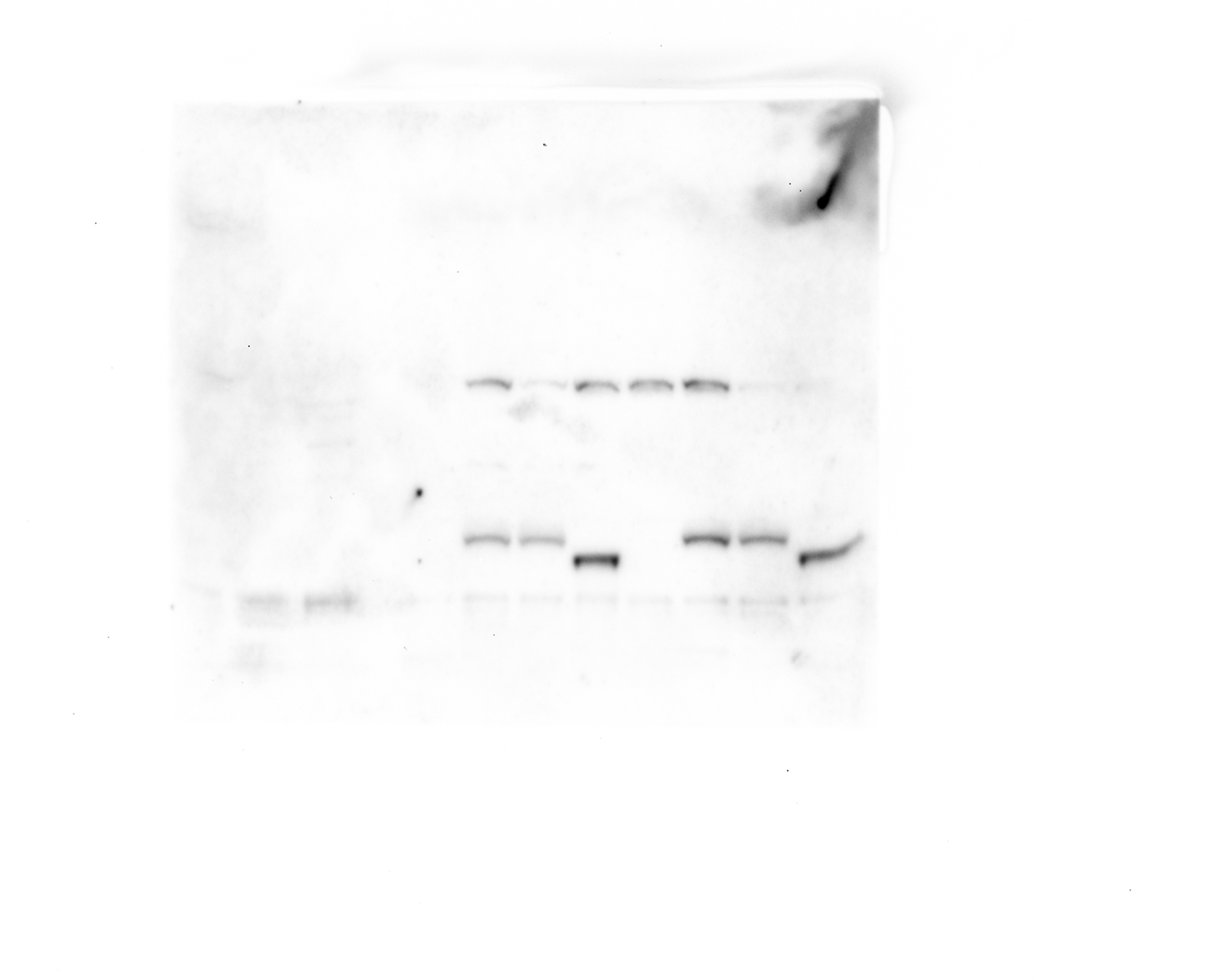

Supplement: Supplementary file 9 — Source data Fig. 5 [file 44319_2026_727_MOESM9_ESM.zip › Figure 5 Source Data/Figure 5C/CHEMI_24052024_091545_15mins_(Chemi).tif]

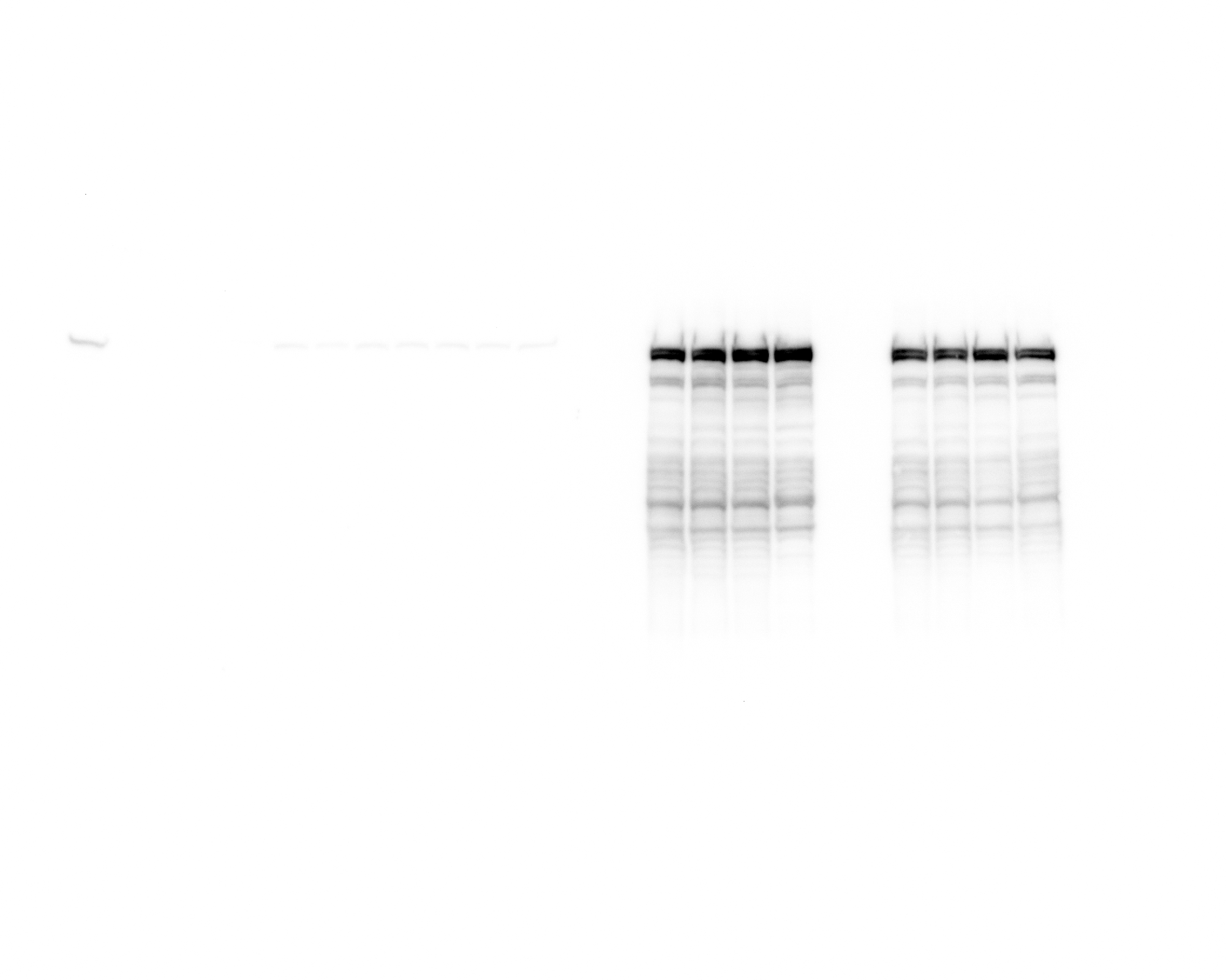

Supplement: Supplementary file 9 — Source data Fig. 5 [file 44319_2026_727_MOESM9_ESM.zip › Figure 5 Source Data/Figure 5C/CHEMI_23052024_091640_15s_(Chemi).tif]

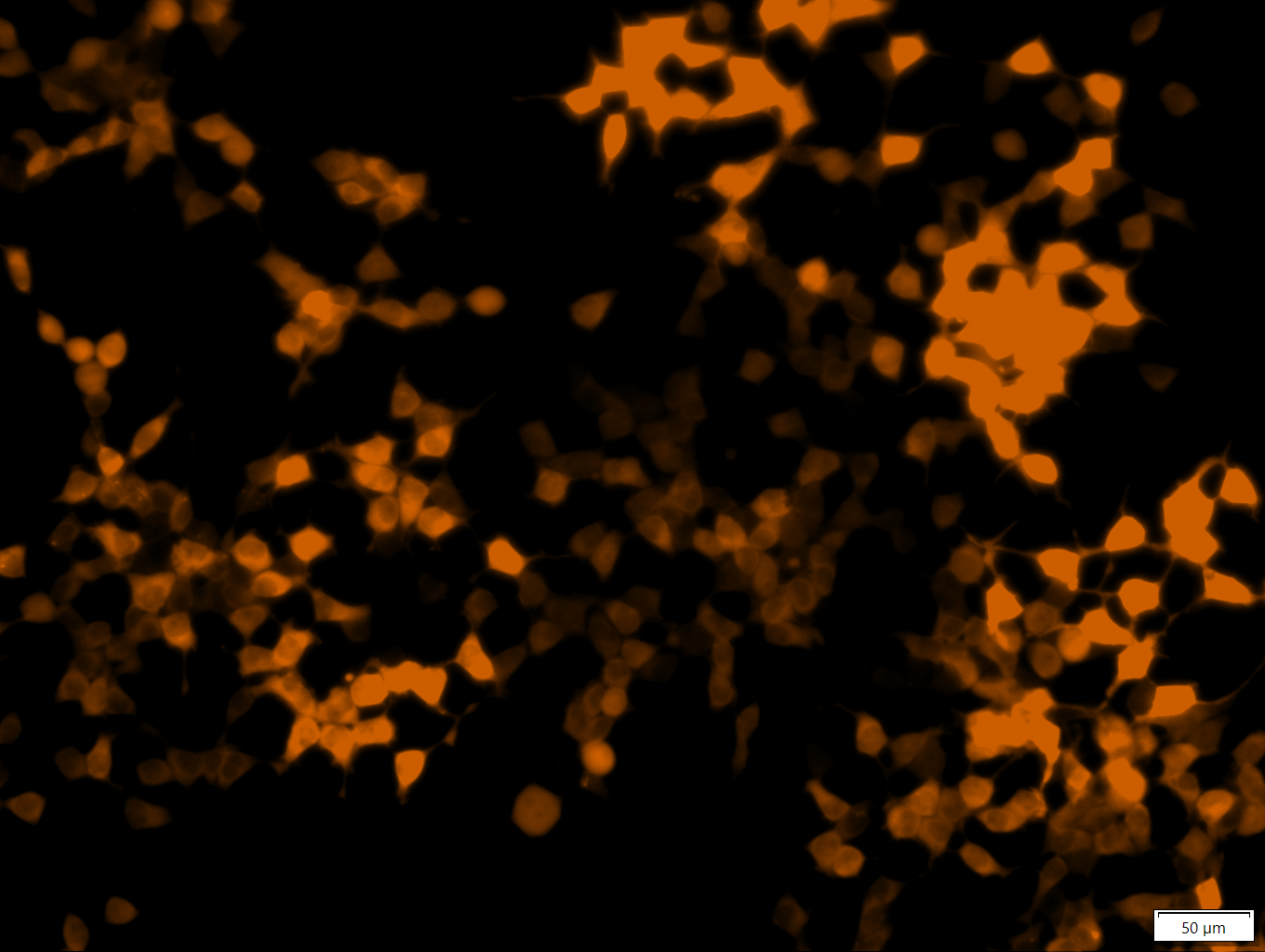

Supplement: Supplementary file 9 — Source data Fig. 5 [file 44319_2026_727_MOESM9_ESM.zip › Figure 5 Source Data/Figure 5D/293T S4 dsRed 2.tif]

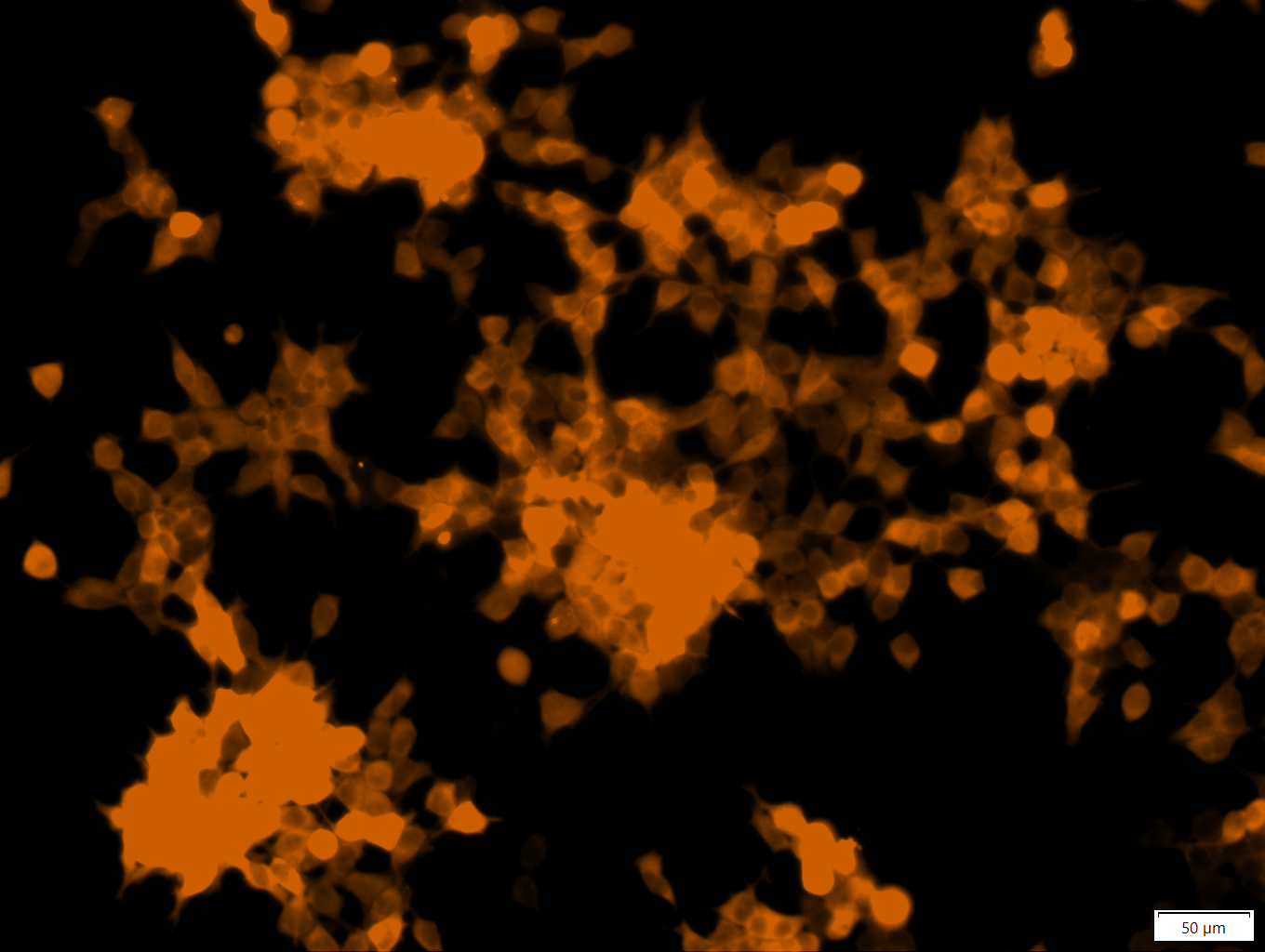

Supplement: Supplementary file 9 — Source data Fig. 5 [file 44319_2026_727_MOESM9_ESM.zip › Figure 5 Source Data/Figure 5D/293T S4 dsRed 1.tif]

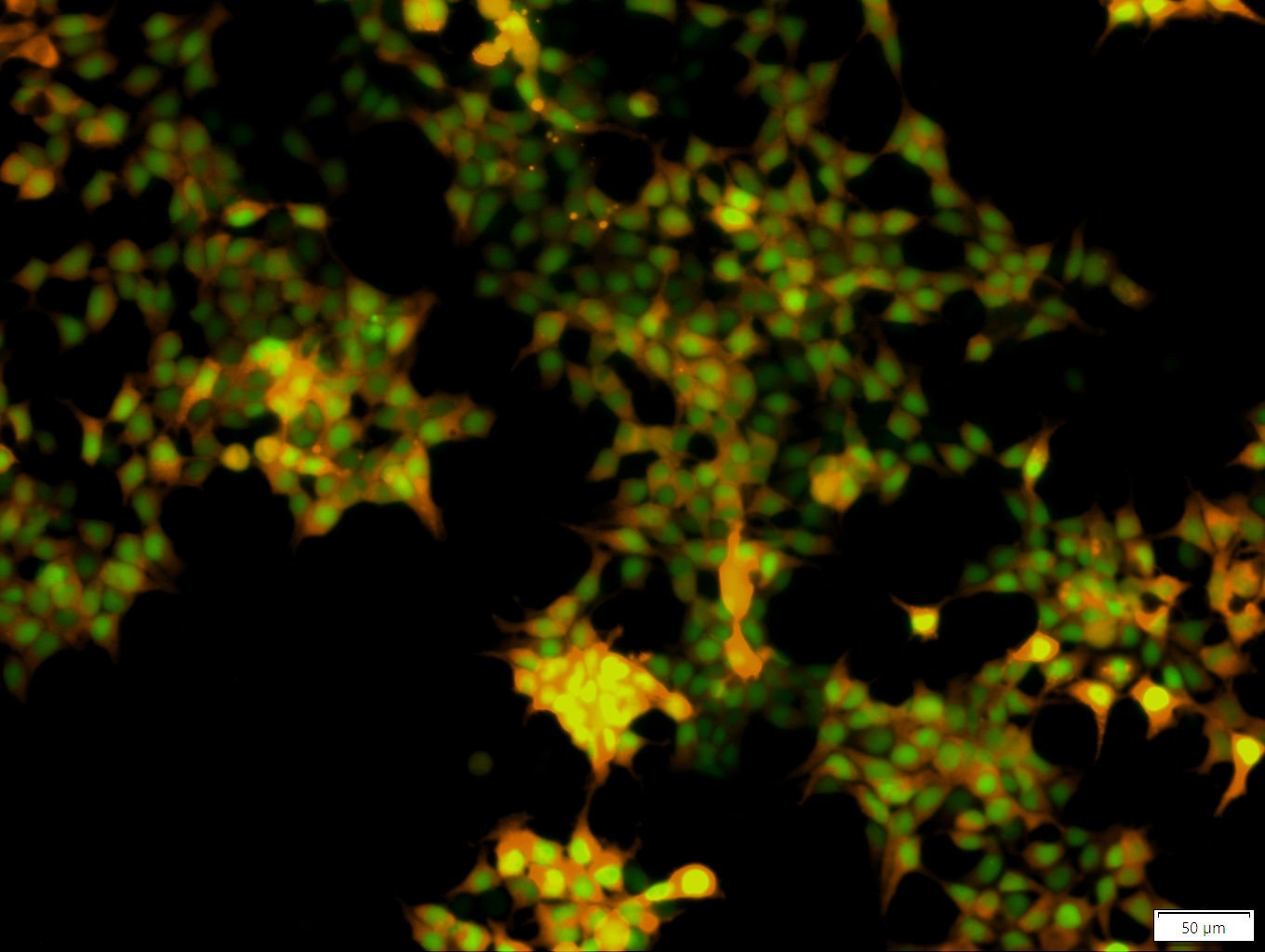

Supplement: Supplementary file 9 — Source data Fig. 5 [file 44319_2026_727_MOESM9_ESM.zip › Figure 5 Source Data/Figure 5D/293T K2D10 S4 overlay 2.tiff]

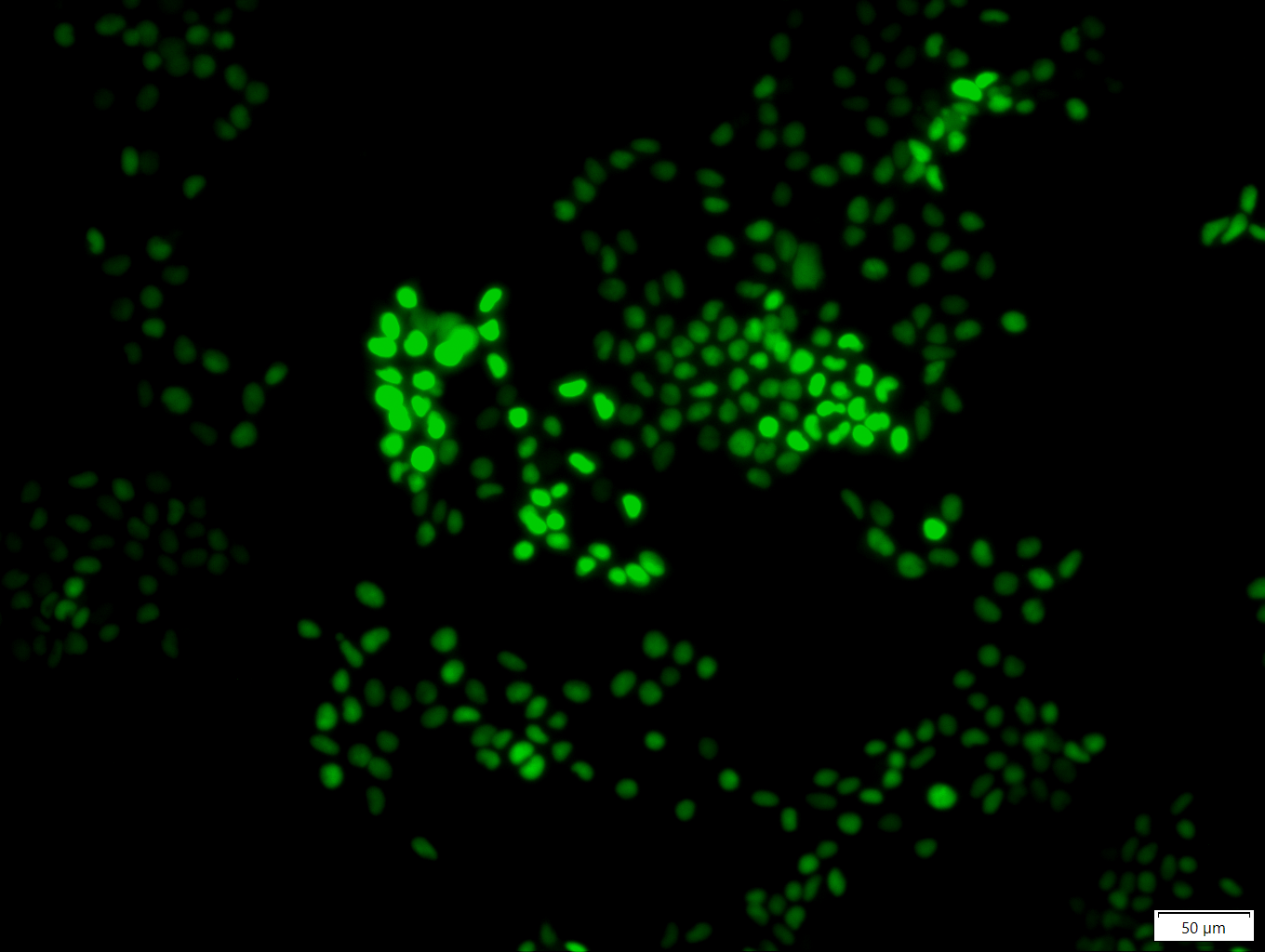

Supplement: Supplementary file 9 — Source data Fig. 5 [file 44319_2026_727_MOESM9_ESM.zip › Figure 5 Source Data/Figure 5D/293T K2D10 S4 GFP 1.tif]

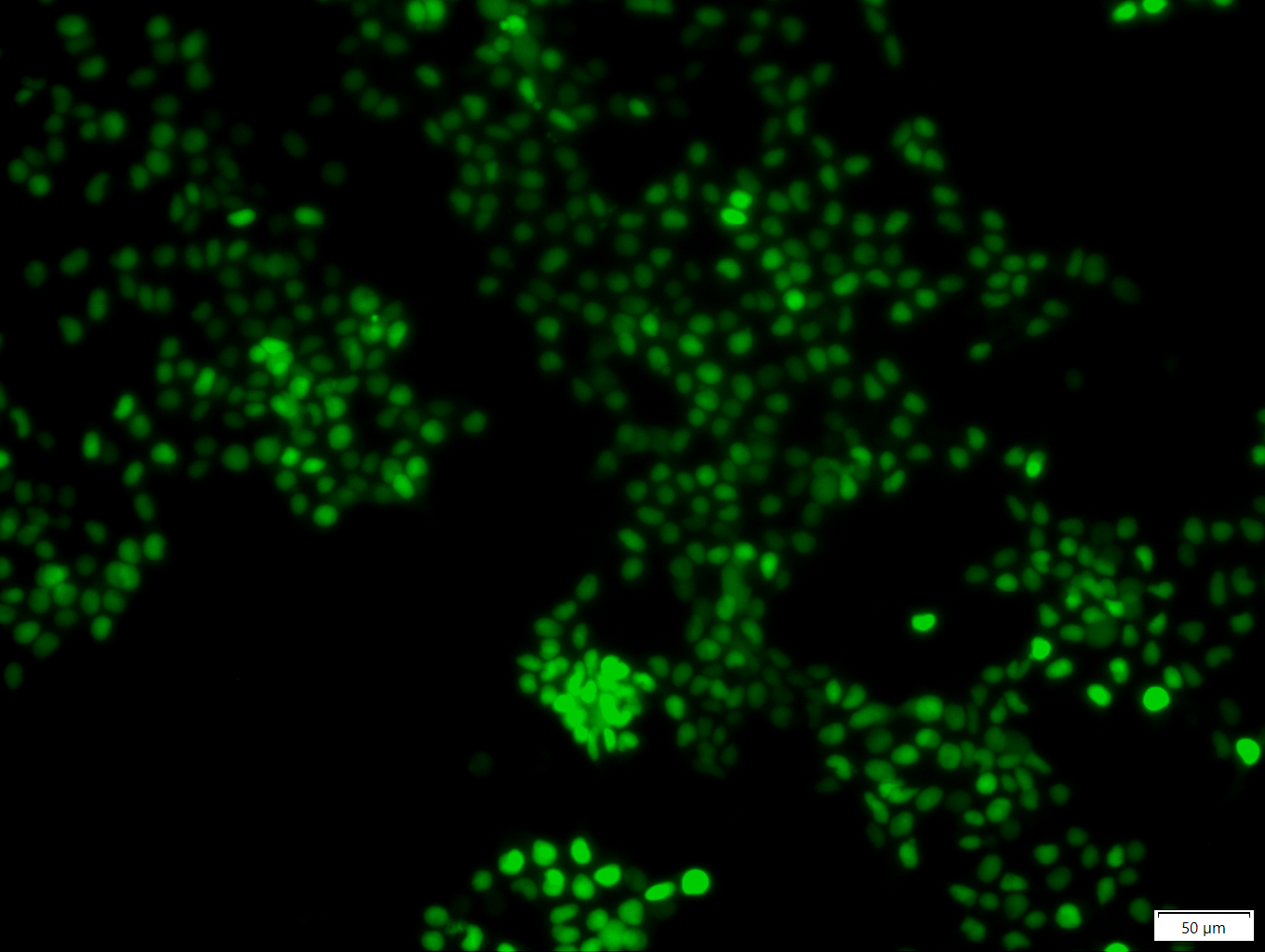

Supplement: Supplementary file 9 — Source data Fig. 5 [file 44319_2026_727_MOESM9_ESM.zip › Figure 5 Source Data/Figure 5D/293T K2D10 S4 GFP 2.tif]

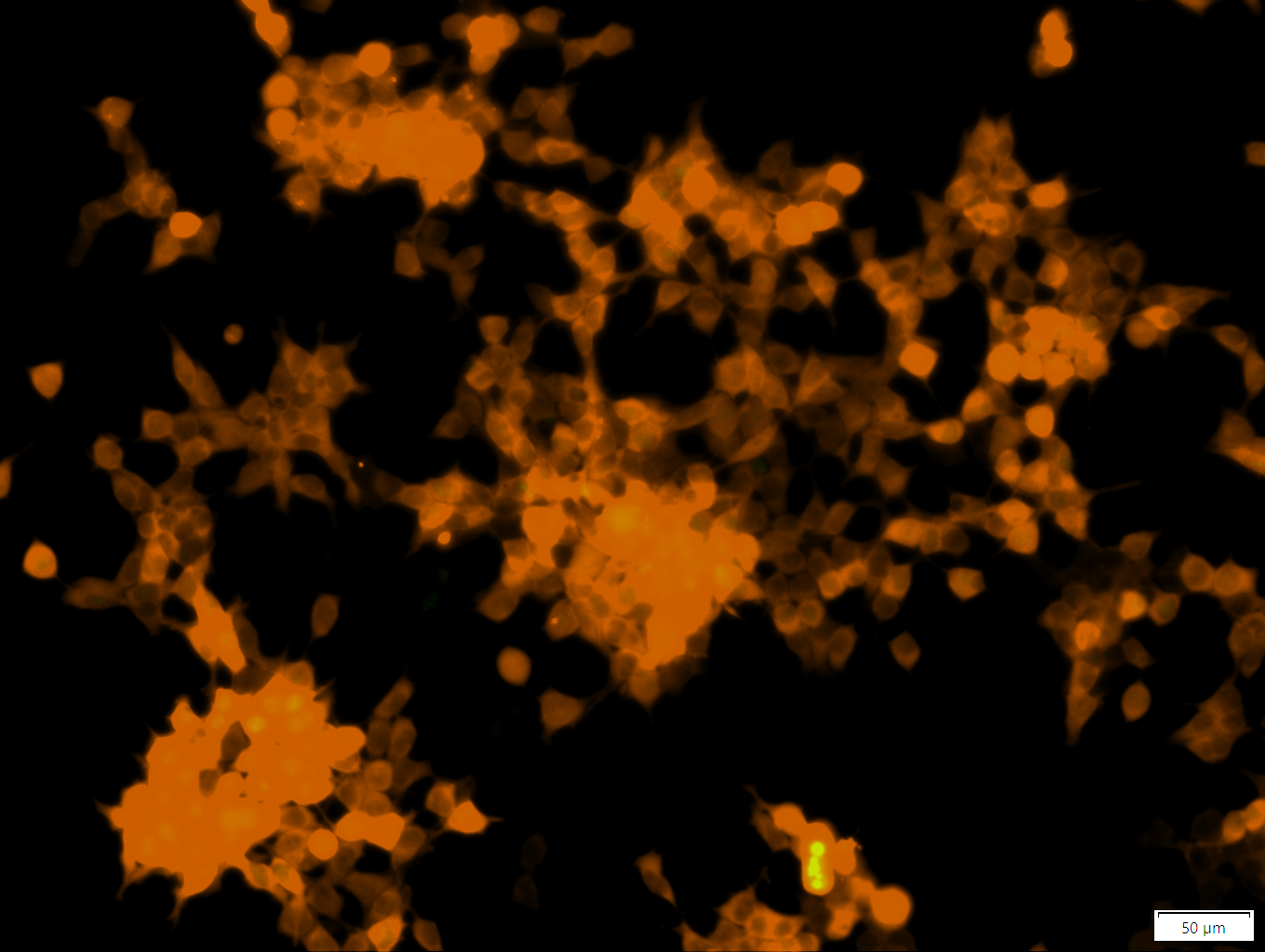

Supplement: Supplementary file 9 — Source data Fig. 5 [file 44319_2026_727_MOESM9_ESM.zip › Figure 5 Source Data/Figure 5D/293T S4 overlay 1.tiff]

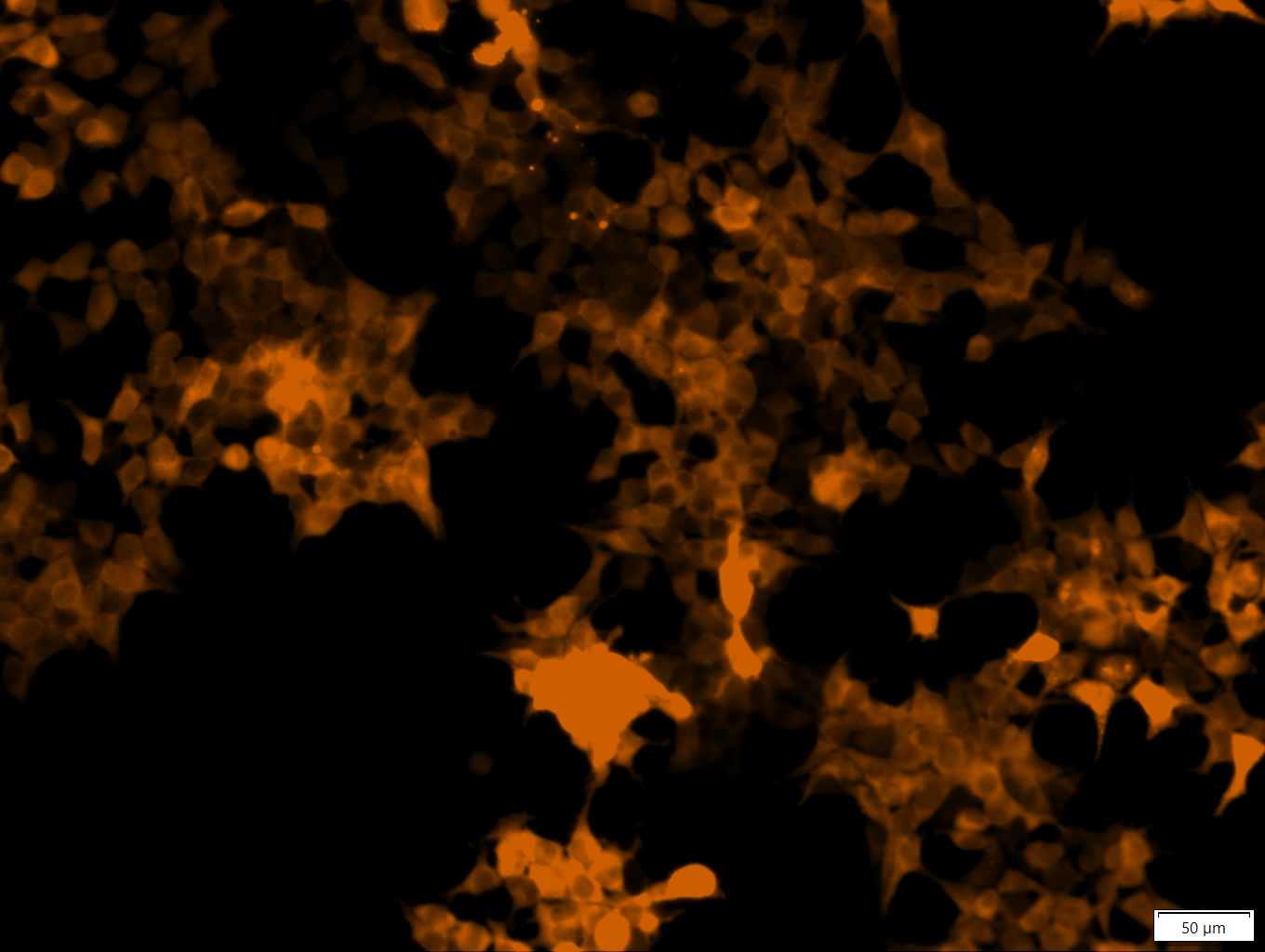

Supplement: Supplementary file 9 — Source data Fig. 5 [file 44319_2026_727_MOESM9_ESM.zip › Figure 5 Source Data/Figure 5D/293T K2D10 S4 dsRed 2.tif]

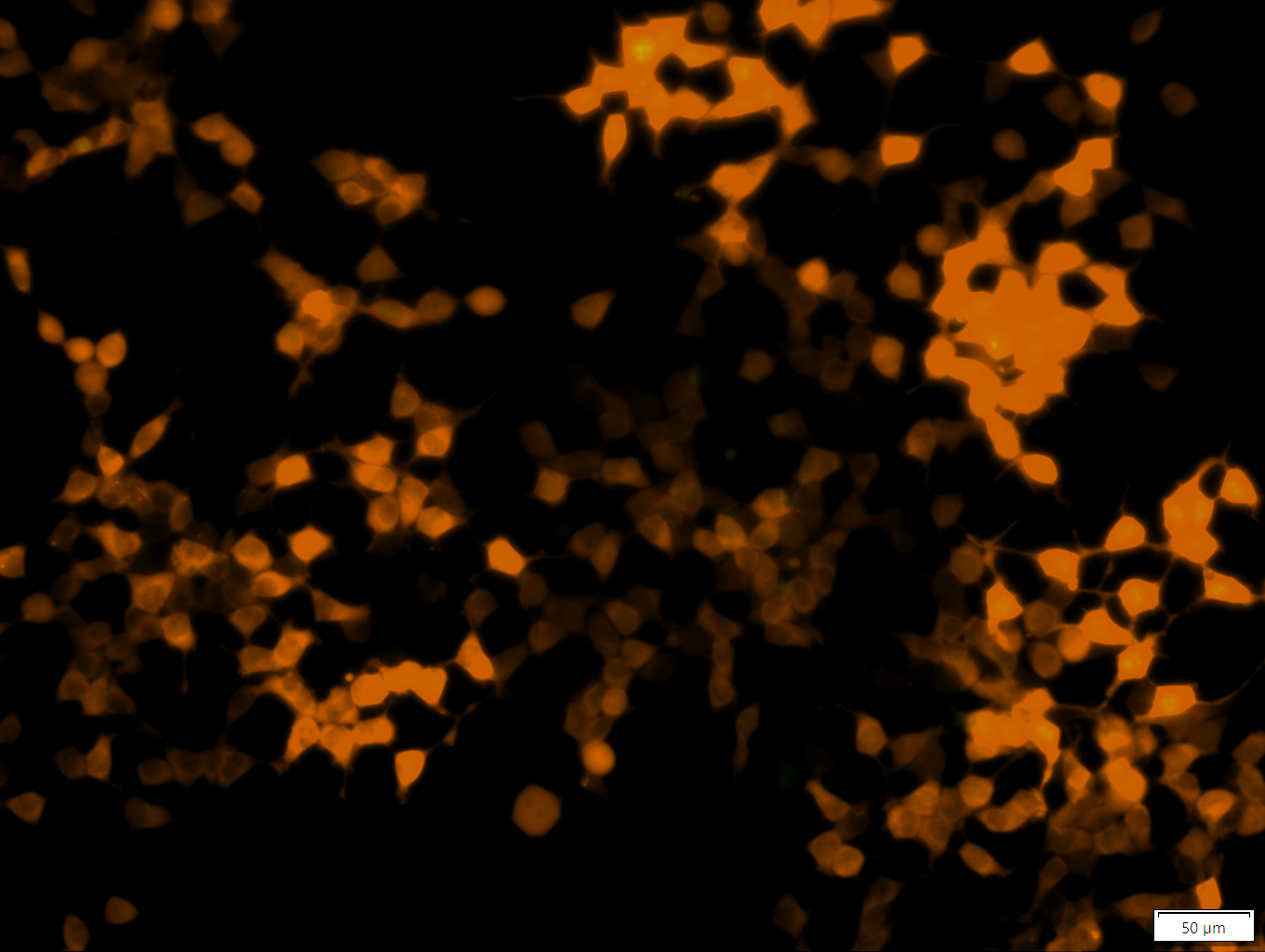

Supplement: Supplementary file 9 — Source data Fig. 5 [file 44319_2026_727_MOESM9_ESM.zip › Figure 5 Source Data/Figure 5D/293T S4 overlay 2.tiff]

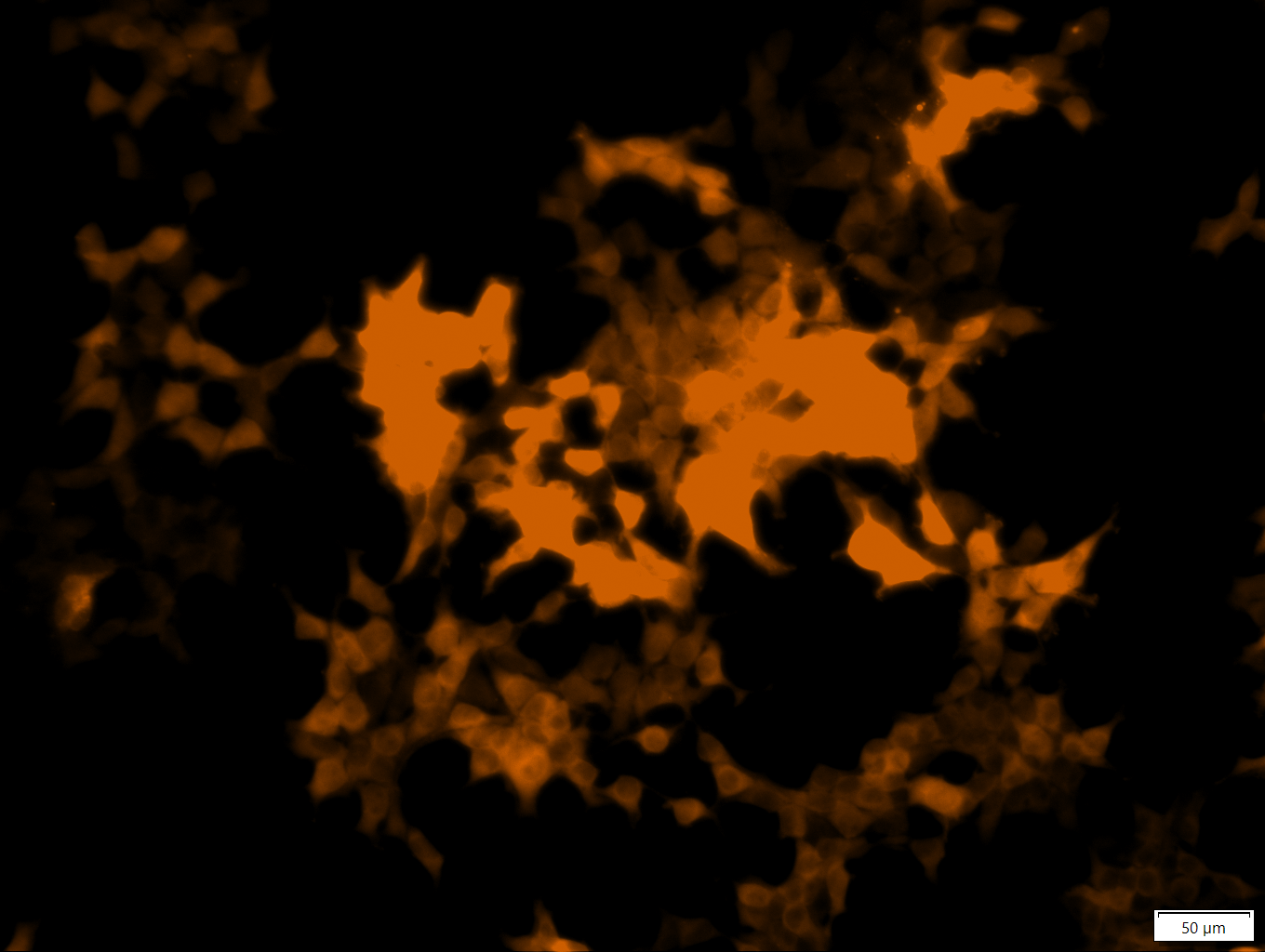

Supplement: Supplementary file 9 — Source data Fig. 5 [file 44319_2026_727_MOESM9_ESM.zip › Figure 5 Source Data/Figure 5D/293T K2D10 S4 dsRed 1.tif]

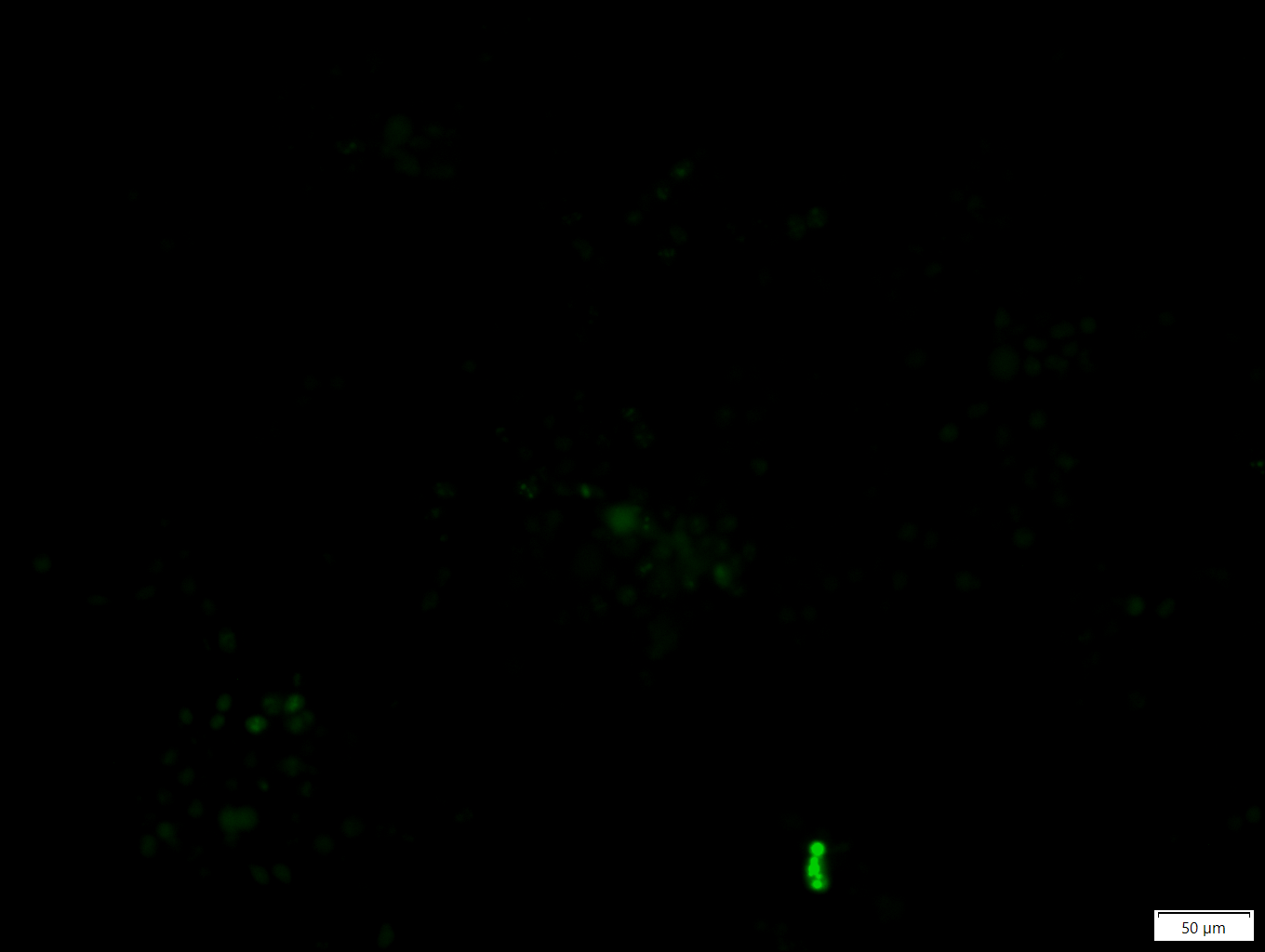

Supplement: Supplementary file 9 — Source data Fig. 5 [file 44319_2026_727_MOESM9_ESM.zip › Figure 5 Source Data/Figure 5D/293T S4 GFP 1.tif]

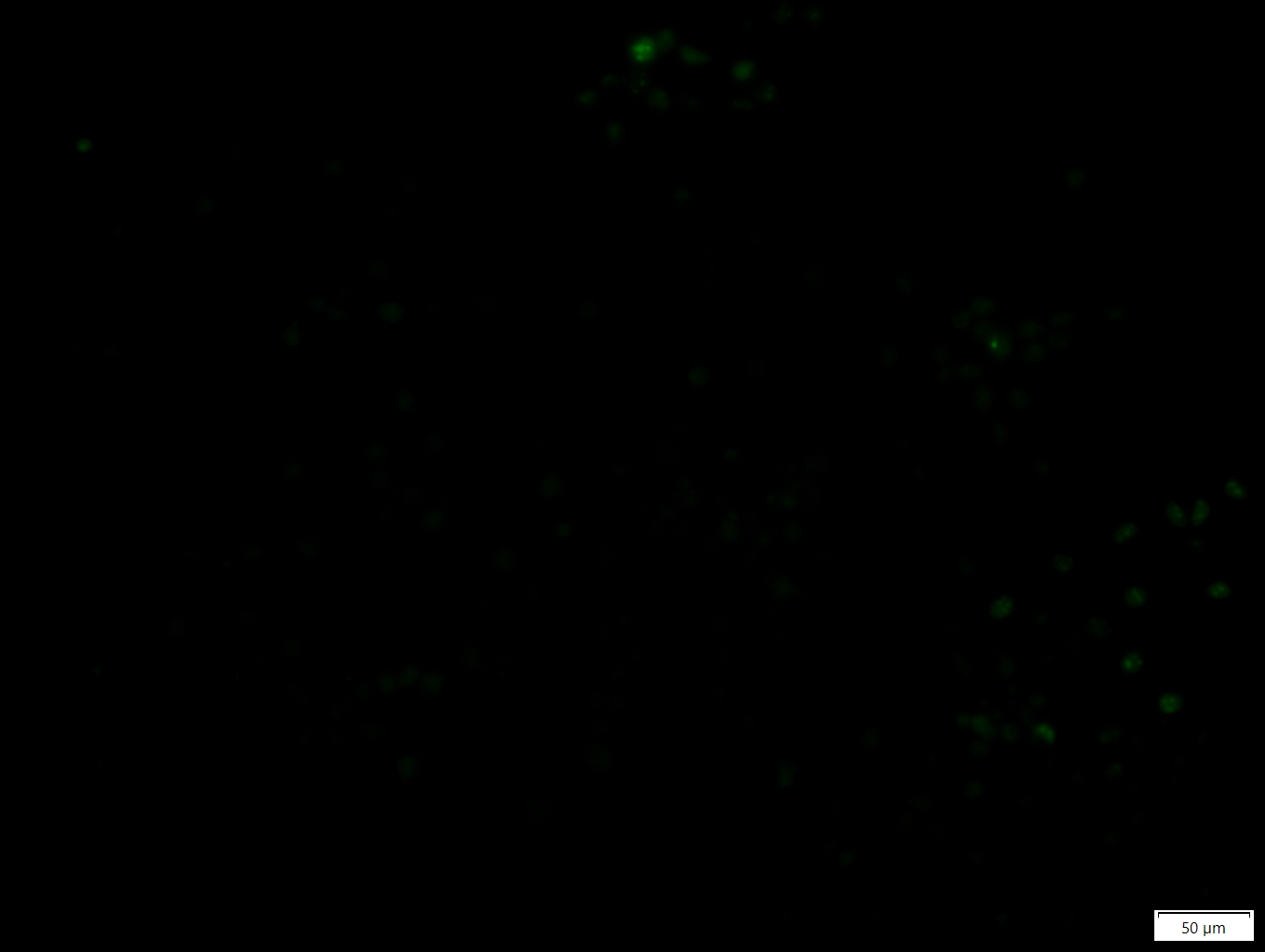

Supplement: Supplementary file 9 — Source data Fig. 5 [file 44319_2026_727_MOESM9_ESM.zip › Figure 5 Source Data/Figure 5D/293T S4 GFP 2.tif]

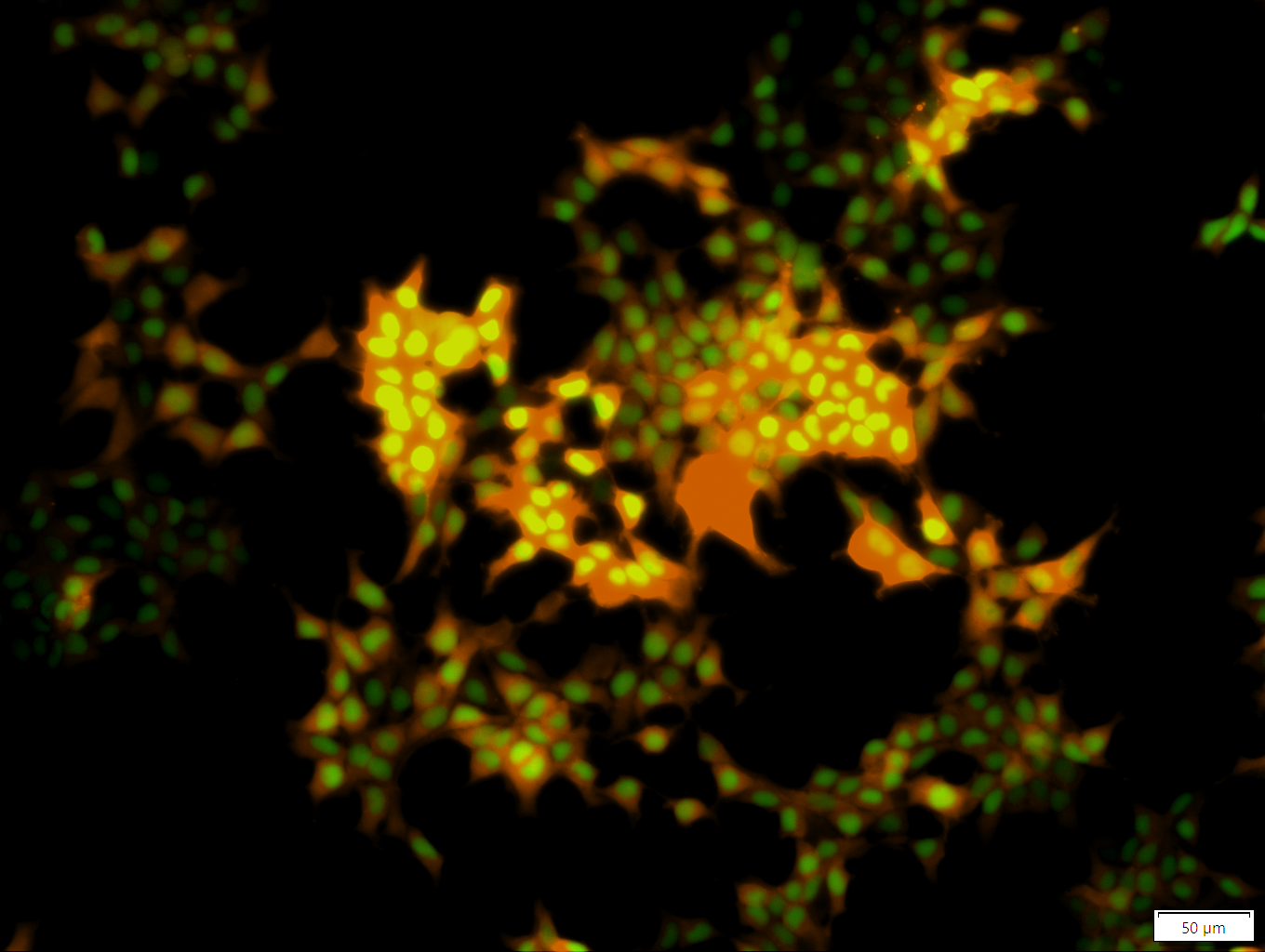

Supplement: Supplementary file 9 — Source data Fig. 5 [file 44319_2026_727_MOESM9_ESM.zip › Figure 5 Source Data/Figure 5D/293T K2D10 S4 overlay 1.tiff]

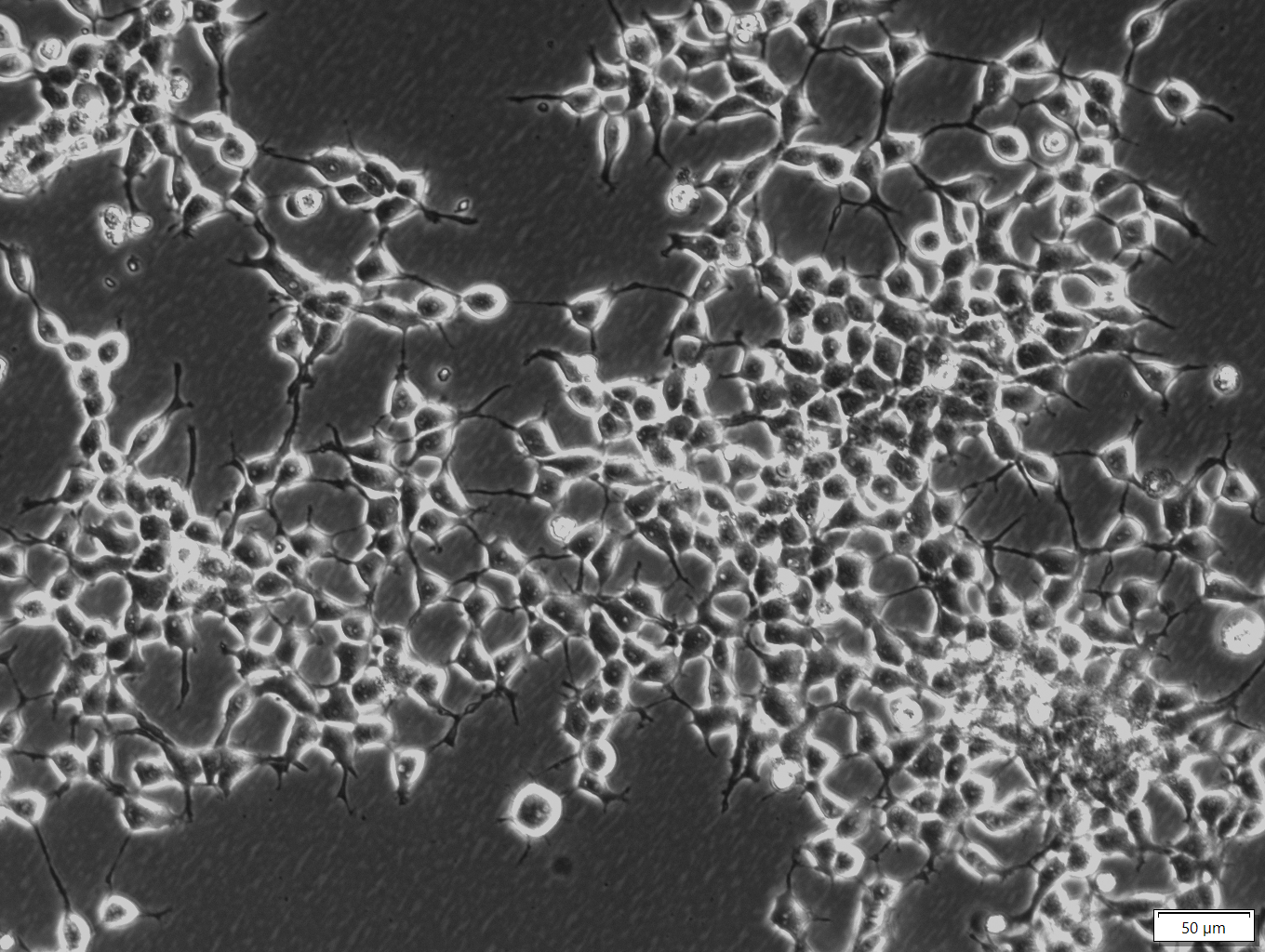

Supplement: Supplementary file 9 — Source data Fig. 5 [file 44319_2026_727_MOESM9_ESM.zip › Figure 5 Source Data/Figure 5D/293T S4 PH 2.tif]

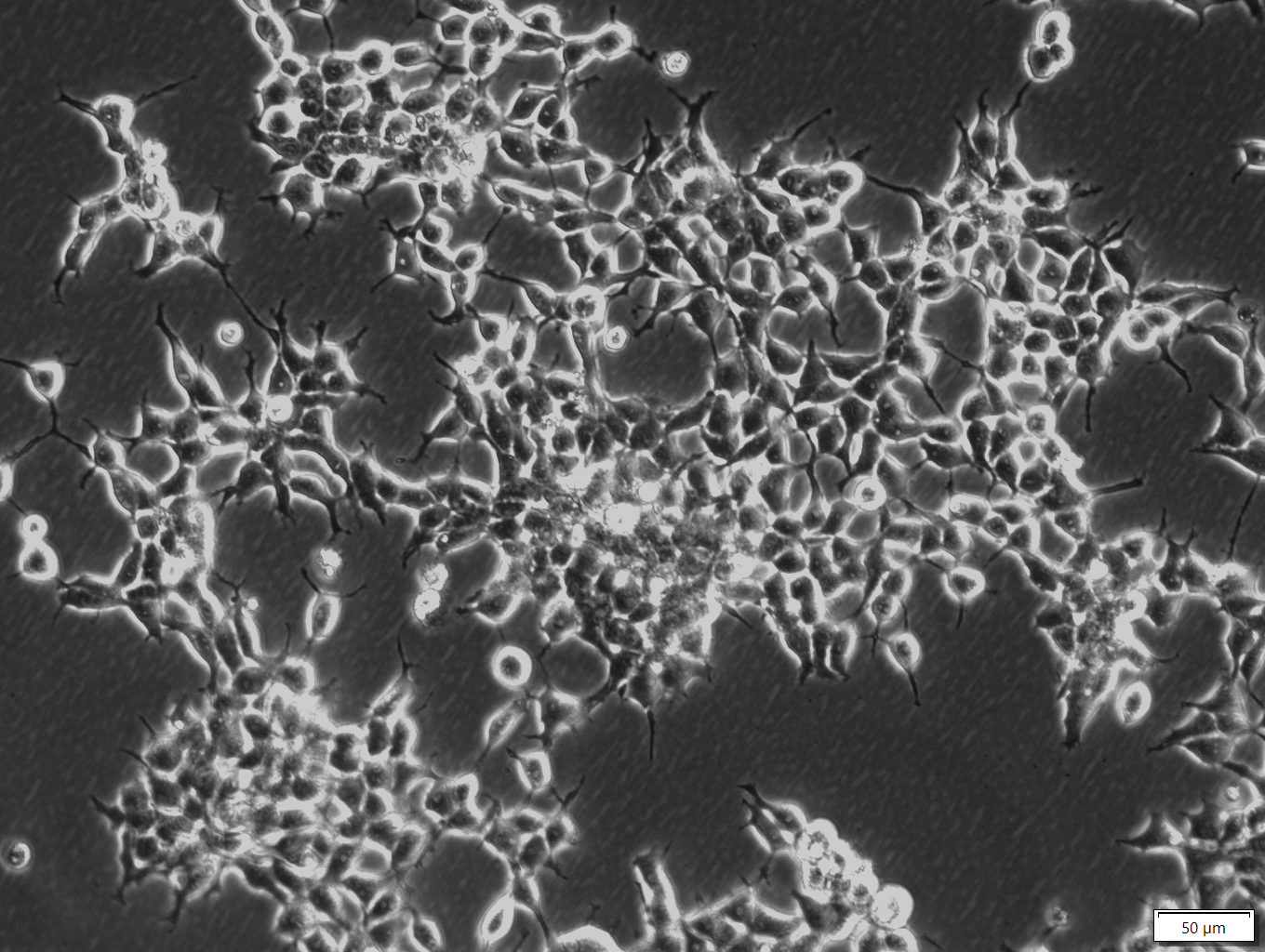

Supplement: Supplementary file 9 — Source data Fig. 5 [file 44319_2026_727_MOESM9_ESM.zip › Figure 5 Source Data/Figure 5D/293T S4 PH 1.tif]

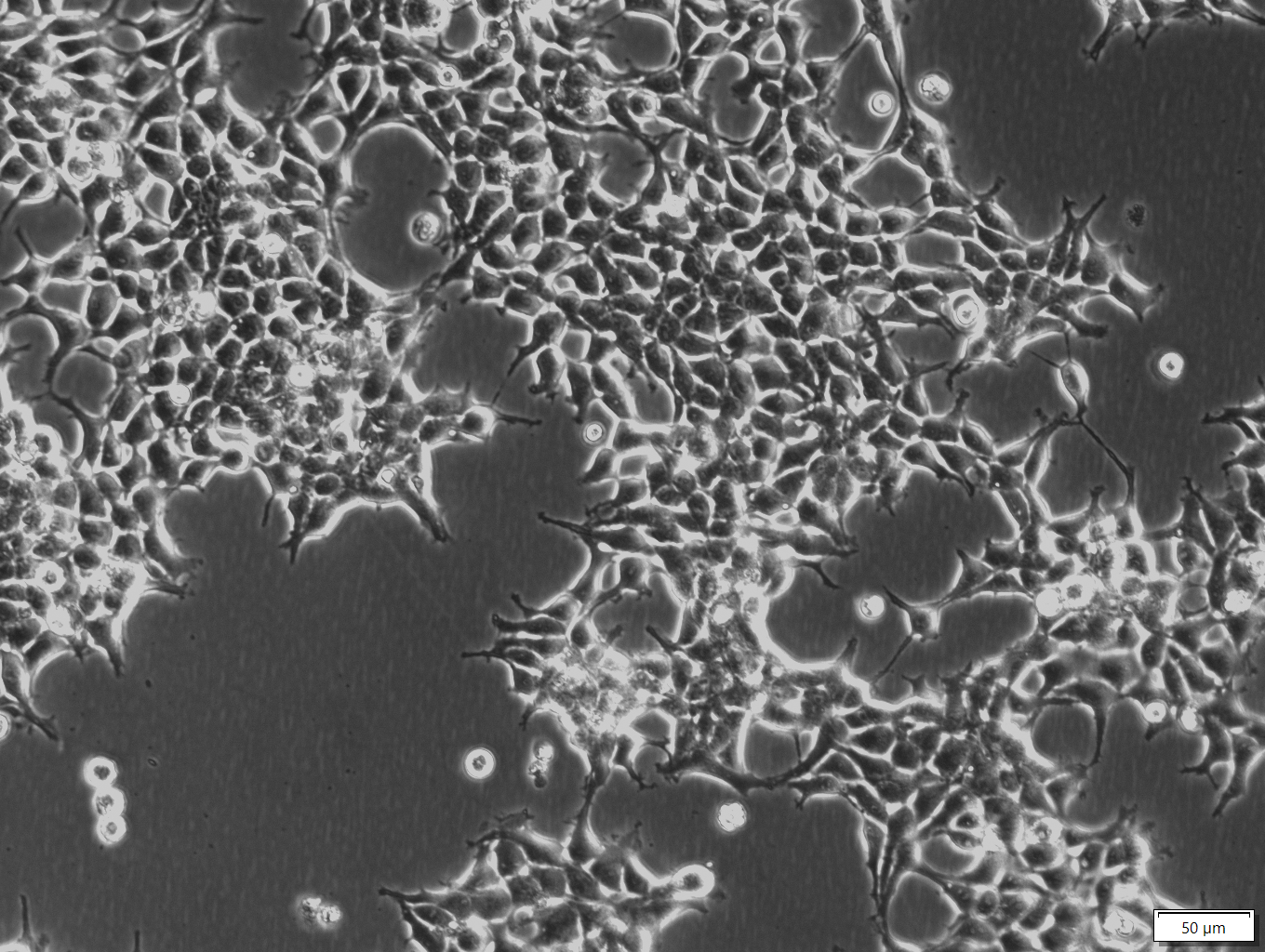

Supplement: Supplementary file 9 — Source data Fig. 5 [file 44319_2026_727_MOESM9_ESM.zip › Figure 5 Source Data/Figure 5D/293T K2D10 S4 PH 2.tif]

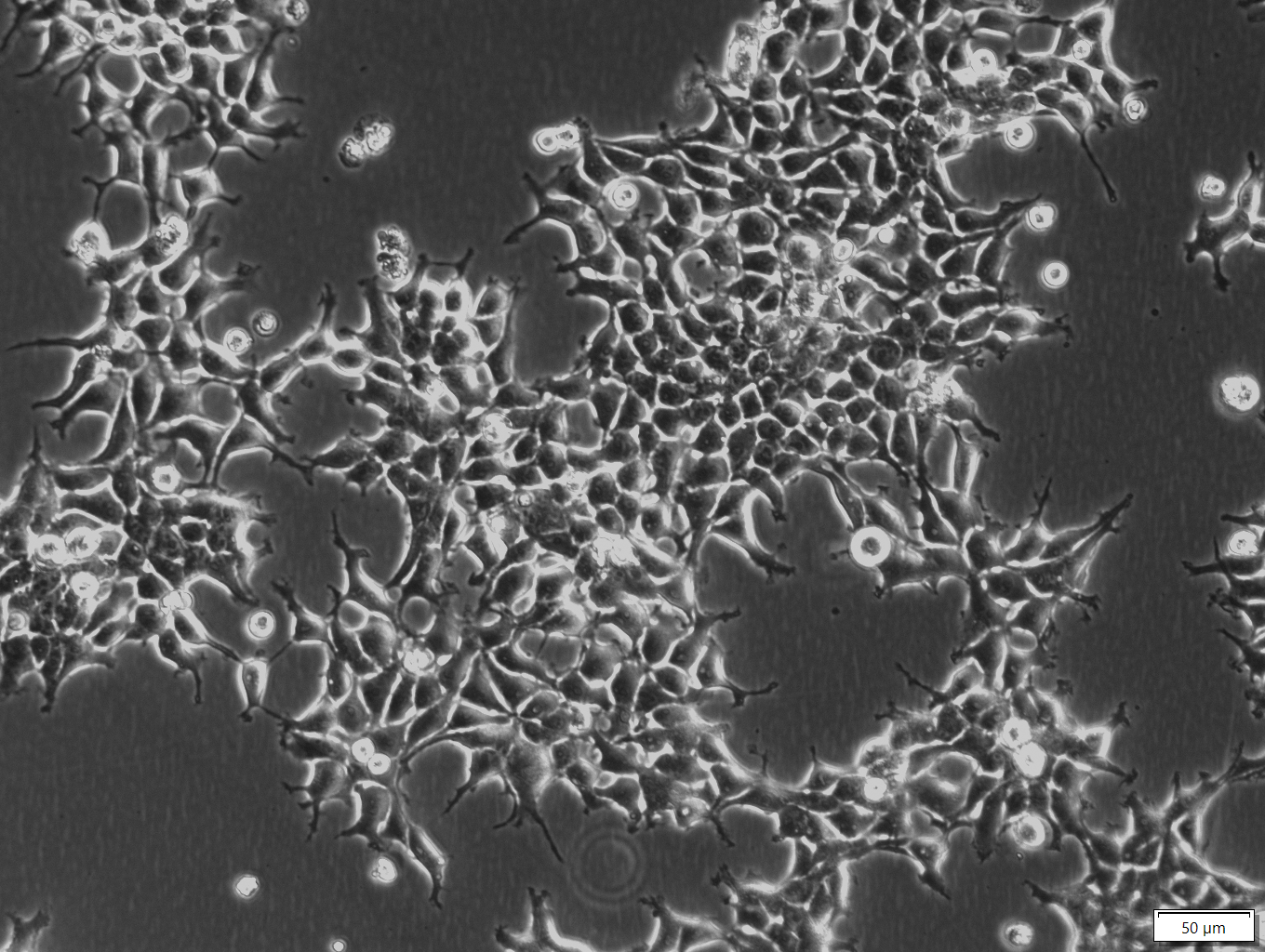

Supplement: Supplementary file 9 — Source data Fig. 5 [file 44319_2026_727_MOESM9_ESM.zip › Figure 5 Source Data/Figure 5D/293T K2D10 S4 PH 1.tif]

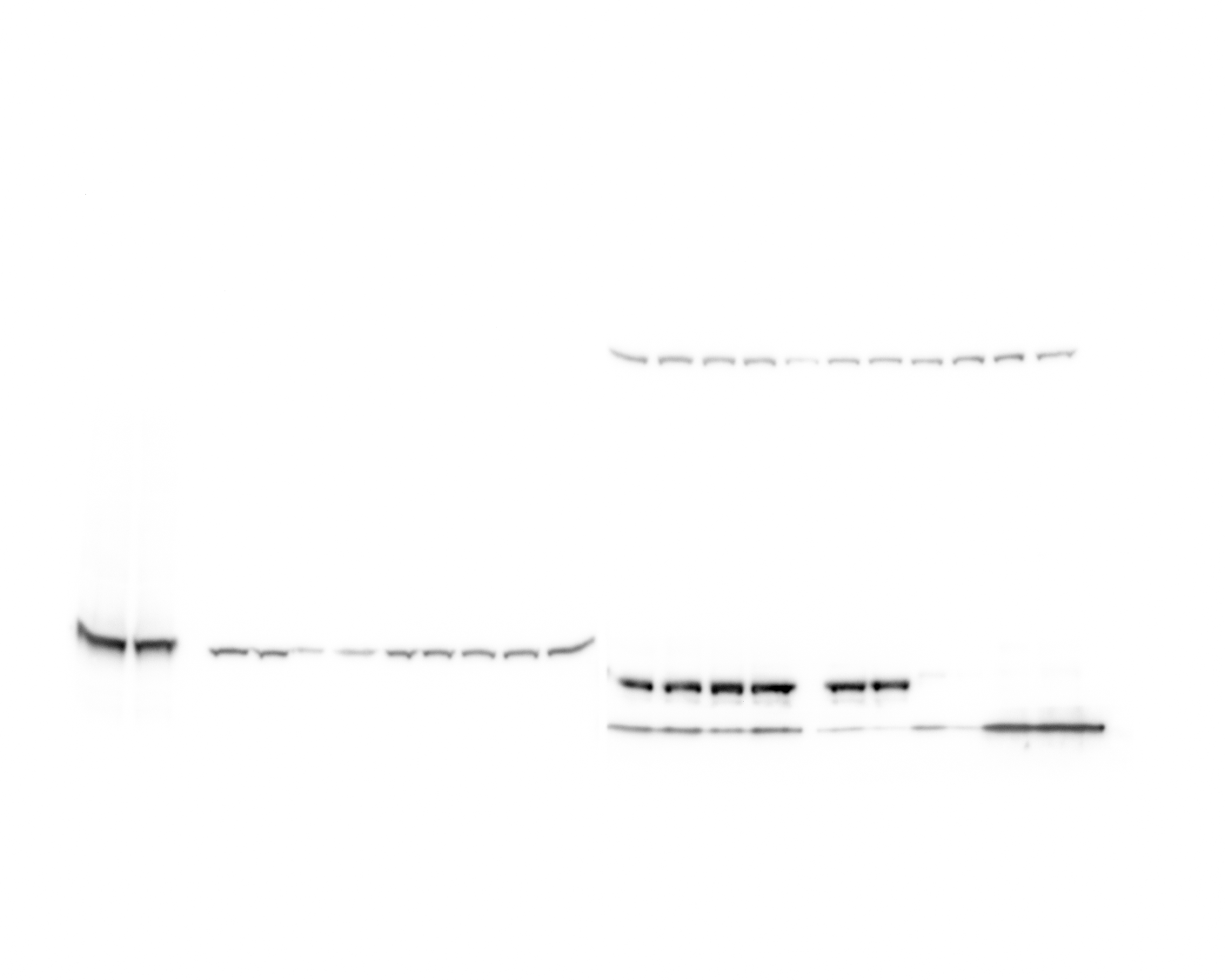

Supplement: Supplementary file 9 — Source data Fig. 5 [file 44319_2026_727_MOESM9_ESM.zip › Figure 5 Source Data/Figure 5B/CHEMI_07052024_132749_10s_(Chemi).tif]

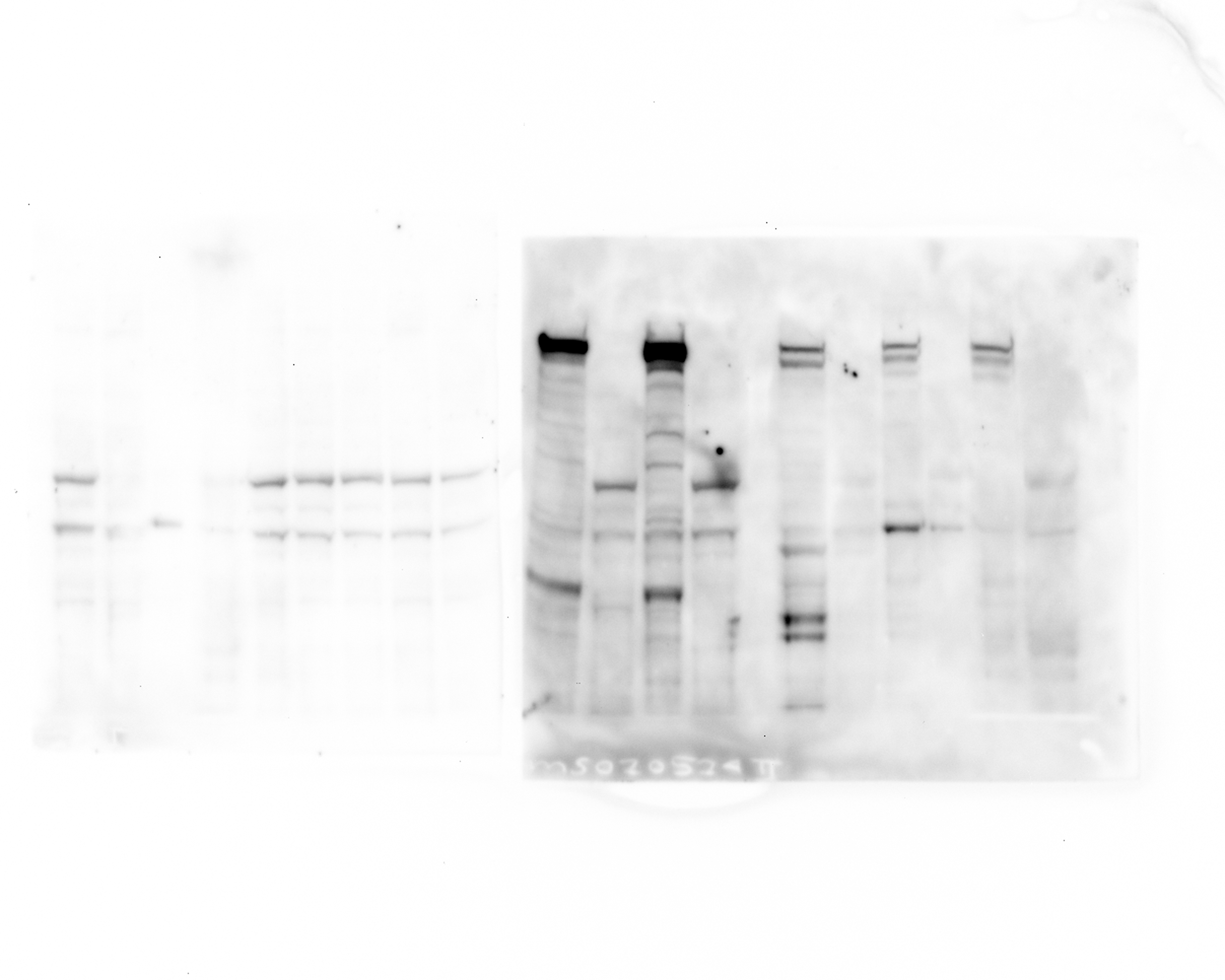

Supplement: Supplementary file 9 — Source data Fig. 5 [file 44319_2026_727_MOESM9_ESM.zip › Figure 5 Source Data/Figure 5B/CHEMI_03052024_162551_15mins_(Chemi).tif]

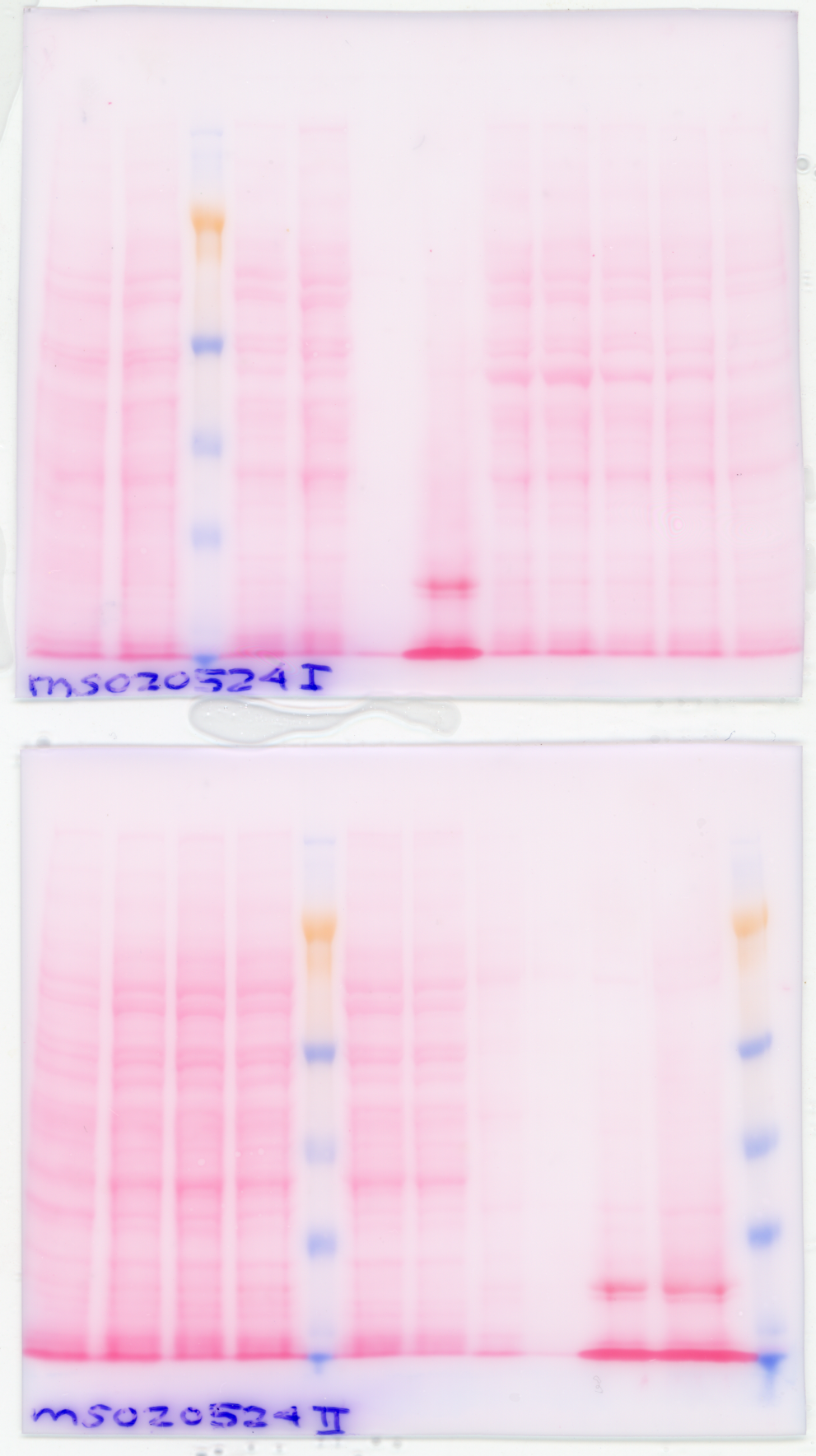

Supplement: Supplementary file 9 — Source data Fig. 5 [file 44319_2026_727_MOESM9_ESM.zip › Figure 5 Source Data/Figure 5B/ms020524_1.tif]

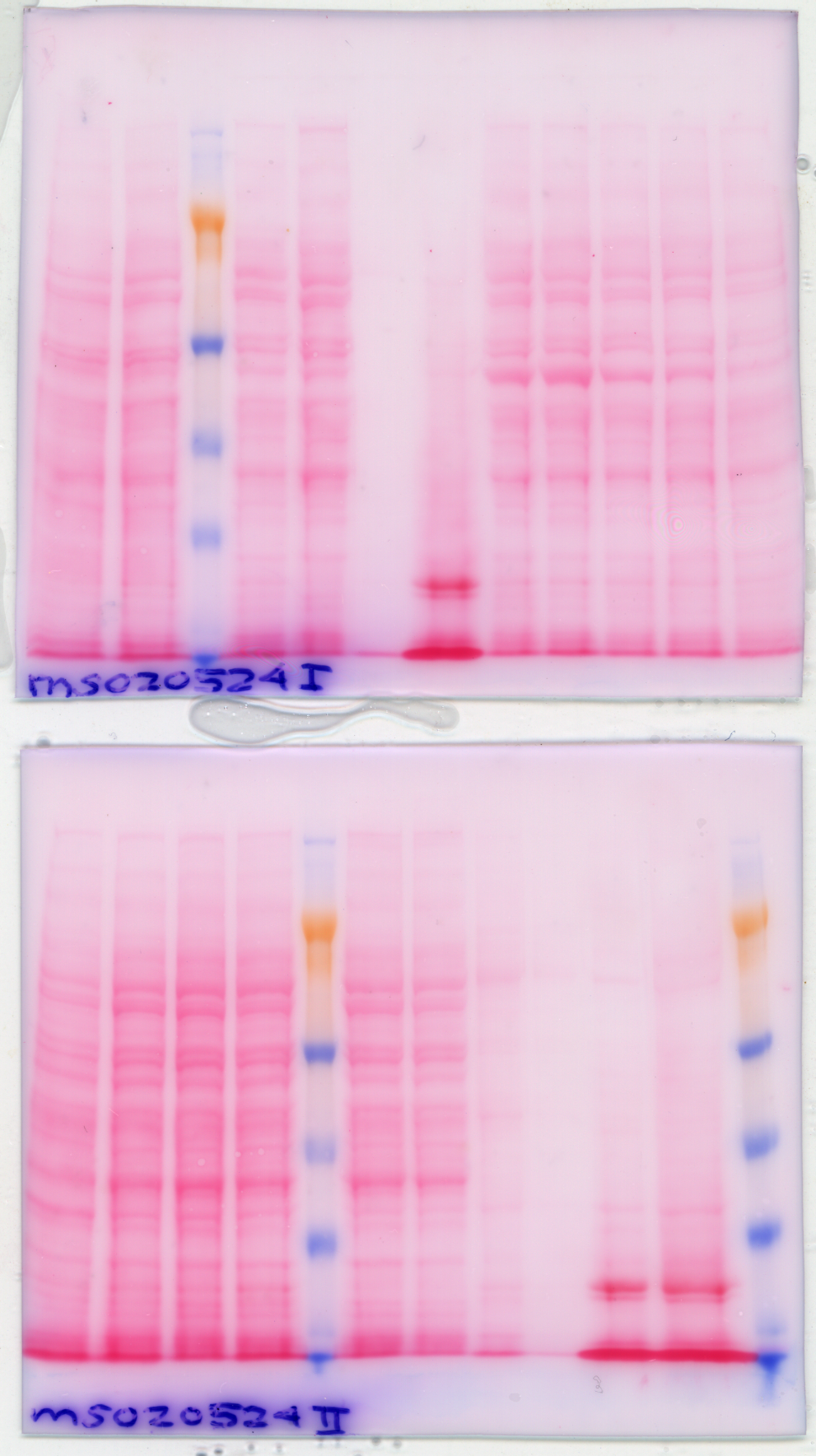

Supplement: Supplementary file 9 — Source data Fig. 5 [file 44319_2026_727_MOESM9_ESM.zip › Figure 5 Source Data/Figure 5B/ms020524_2.tif]

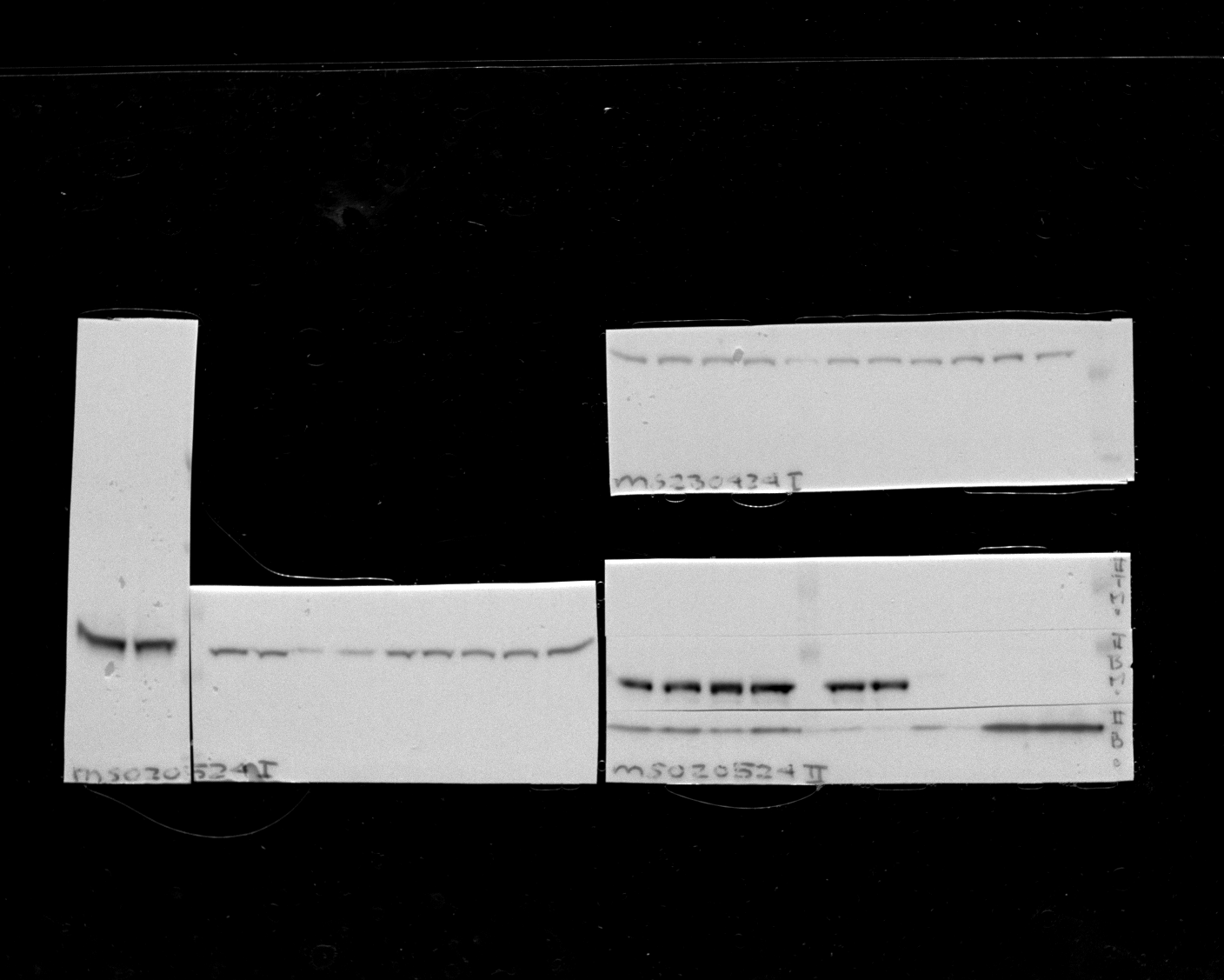

Supplement: Supplementary file 9 — Source data Fig. 5 [file 44319_2026_727_MOESM9_ESM.zip › Figure 5 Source Data/Figure 5B/CHEMI_07052024_132749_10s_(Chemi) overlay.tif]

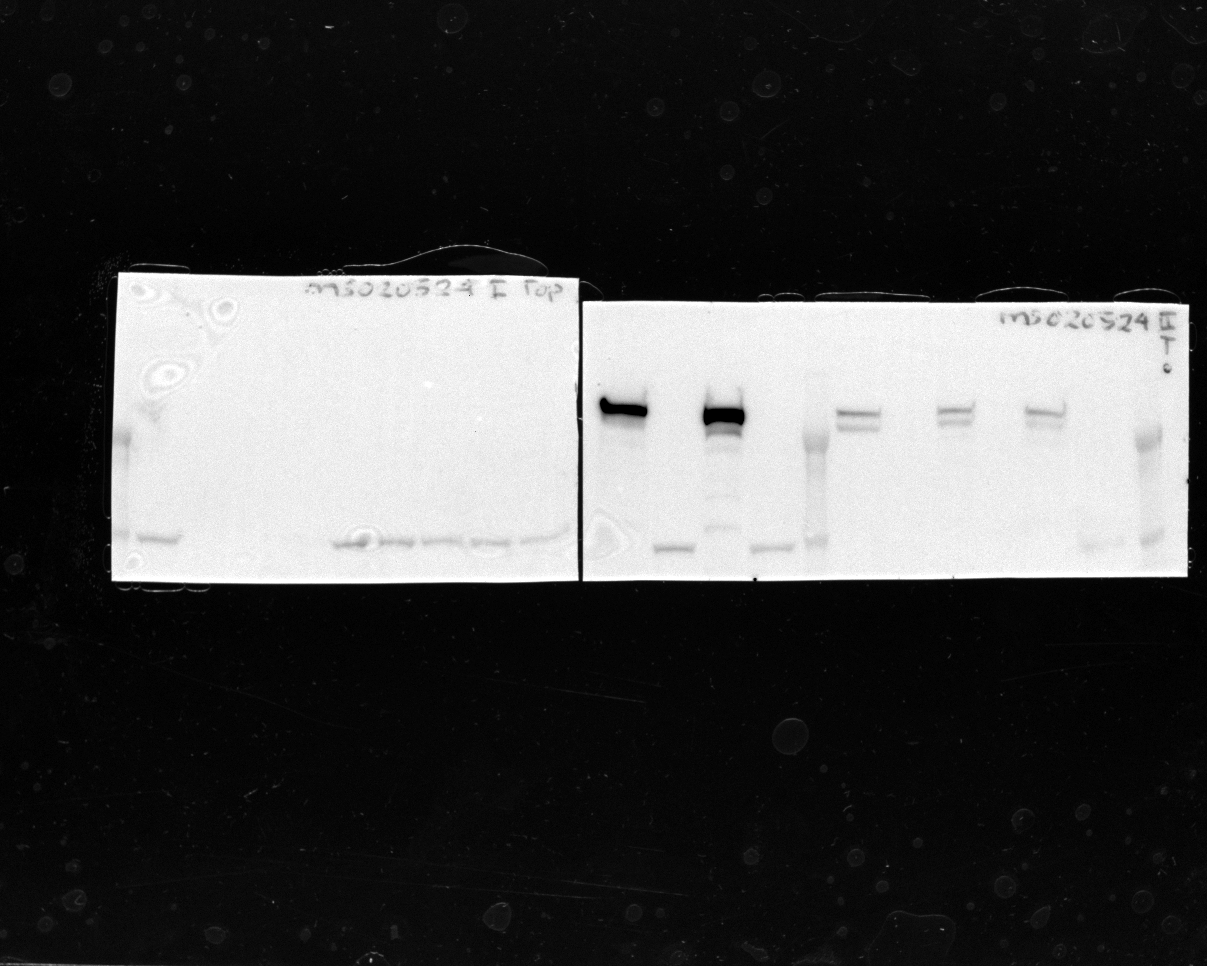

Supplement: Supplementary file 9 — Source data Fig. 5 [file 44319_2026_727_MOESM9_ESM.zip › Figure 5 Source Data/Figure 5B/CHEMI_07052024_130845_20mins_(Chemi) overlay.tif]

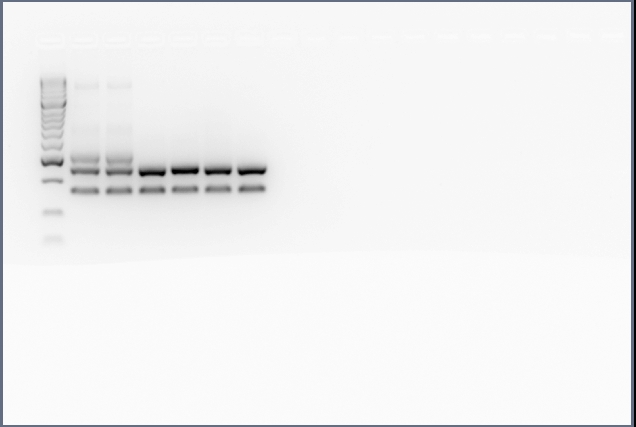

Supplement: Supplementary file 11 — Appendix Figure S2 Source Data [file 44319_2026_727_MOESM11_ESM.zip › Appendix Figure S2 Source data/Panel 2E/wcastillo 2020-09-23 15hr 14min.jpg]

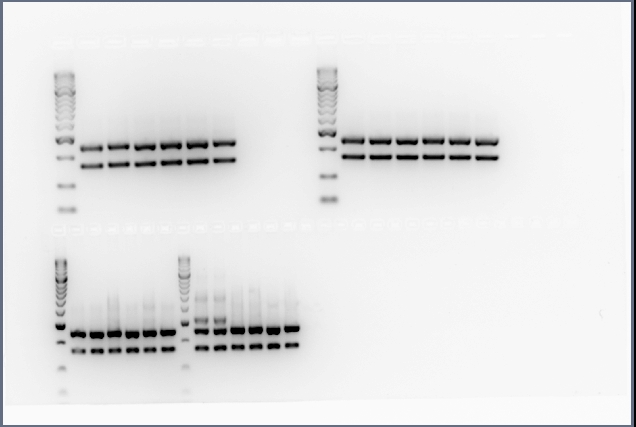

Supplement: Supplementary file 11 — Appendix Figure S2 Source Data [file 44319_2026_727_MOESM11_ESM.zip › Appendix Figure S2 Source data/Panel 2E/wcastillo 2020-09-21 17hr 33min.jpg]

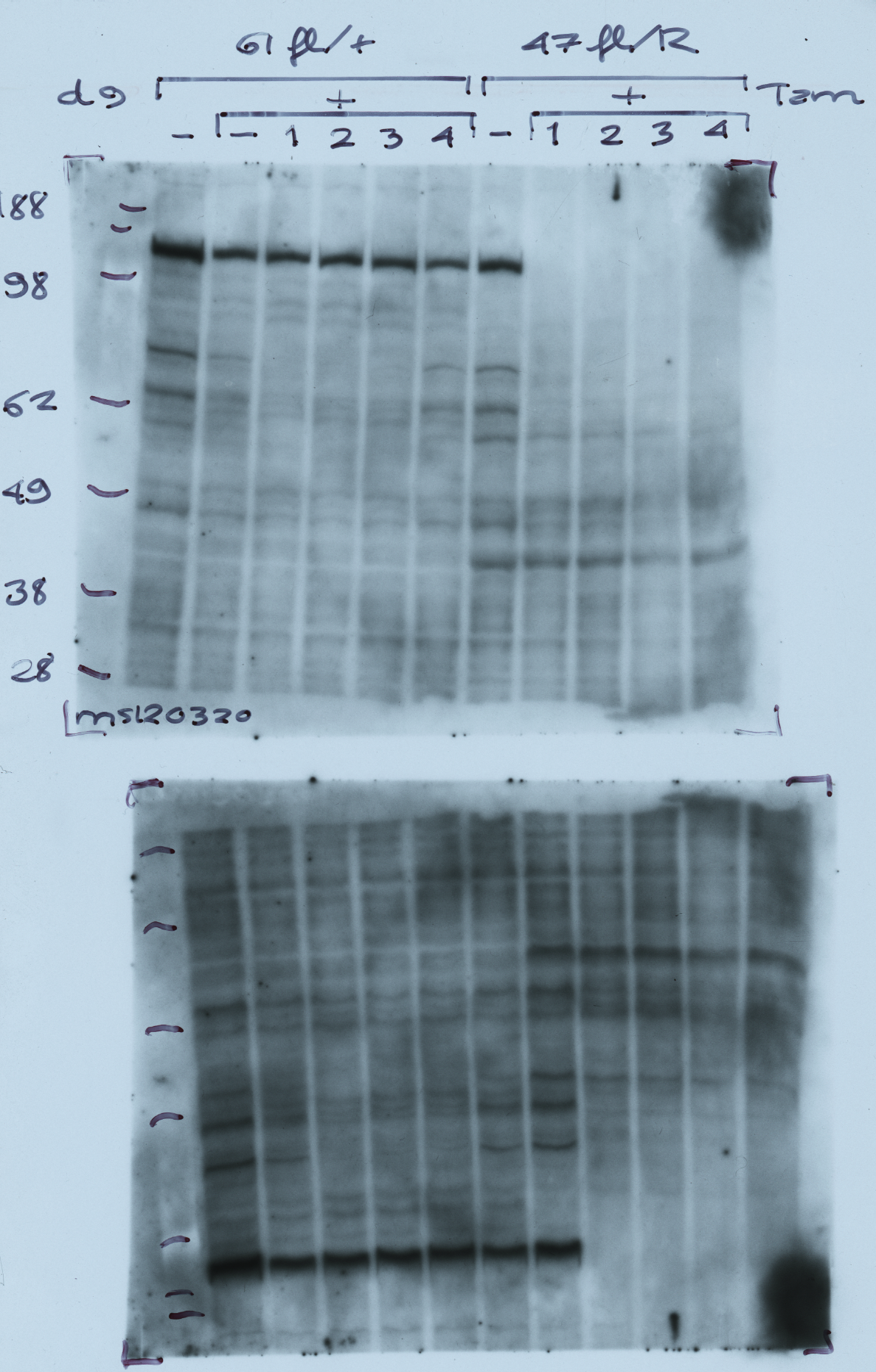

Supplement: Supplementary file 11 — Appendix Figure S2 Source Data [file 44319_2026_727_MOESM11_ESM.zip › Appendix Figure S2 Source data/Panel 2F/ms130320 Klhdc3 3B10002.tif]

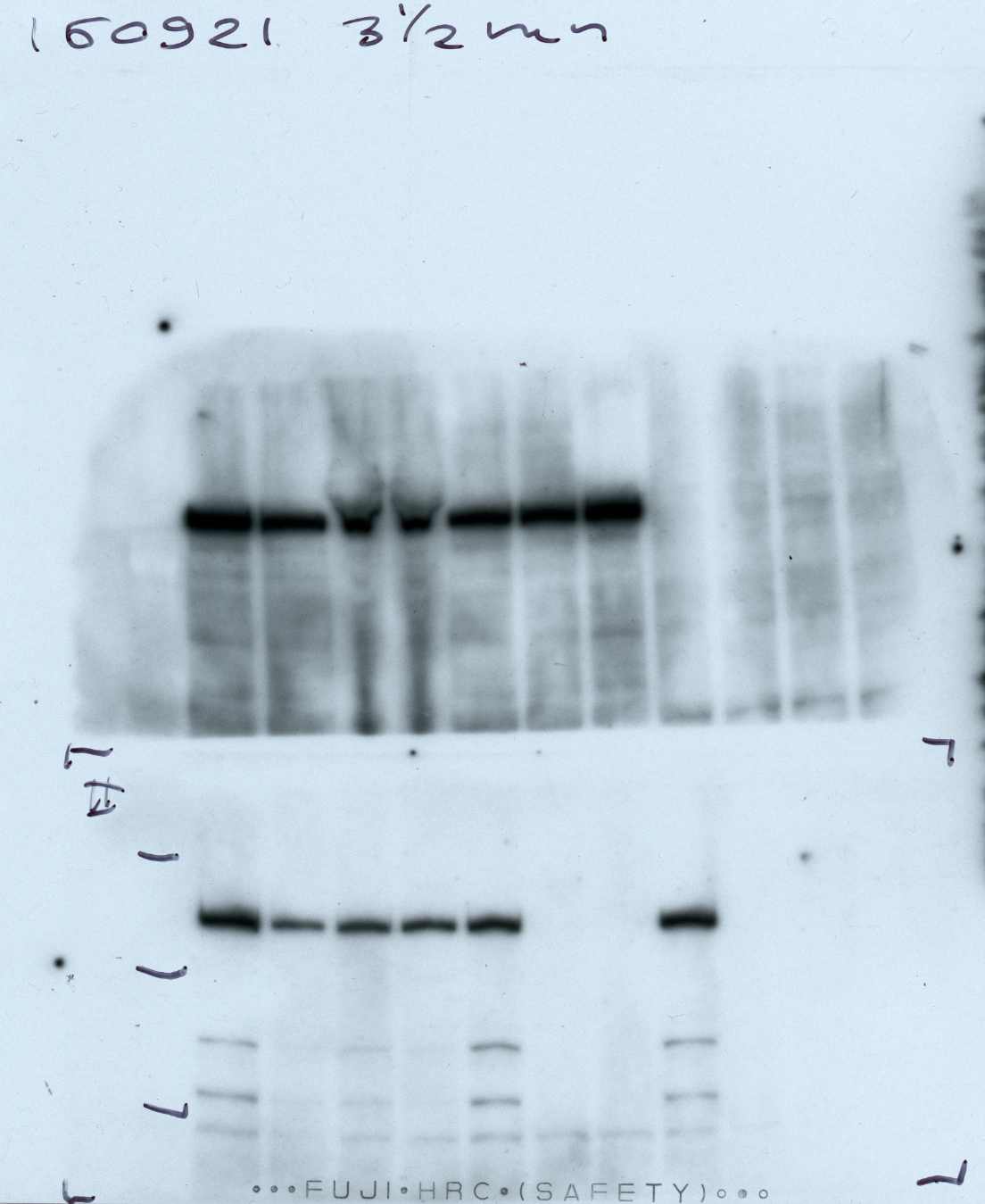

Supplement: Supplementary file 11 — Appendix Figure S2 Source Data [file 44319_2026_727_MOESM11_ESM.zip › Appendix Figure S2 Source data/Panel 2G/ms160921 I and II RECQL4 002 copy.tif]

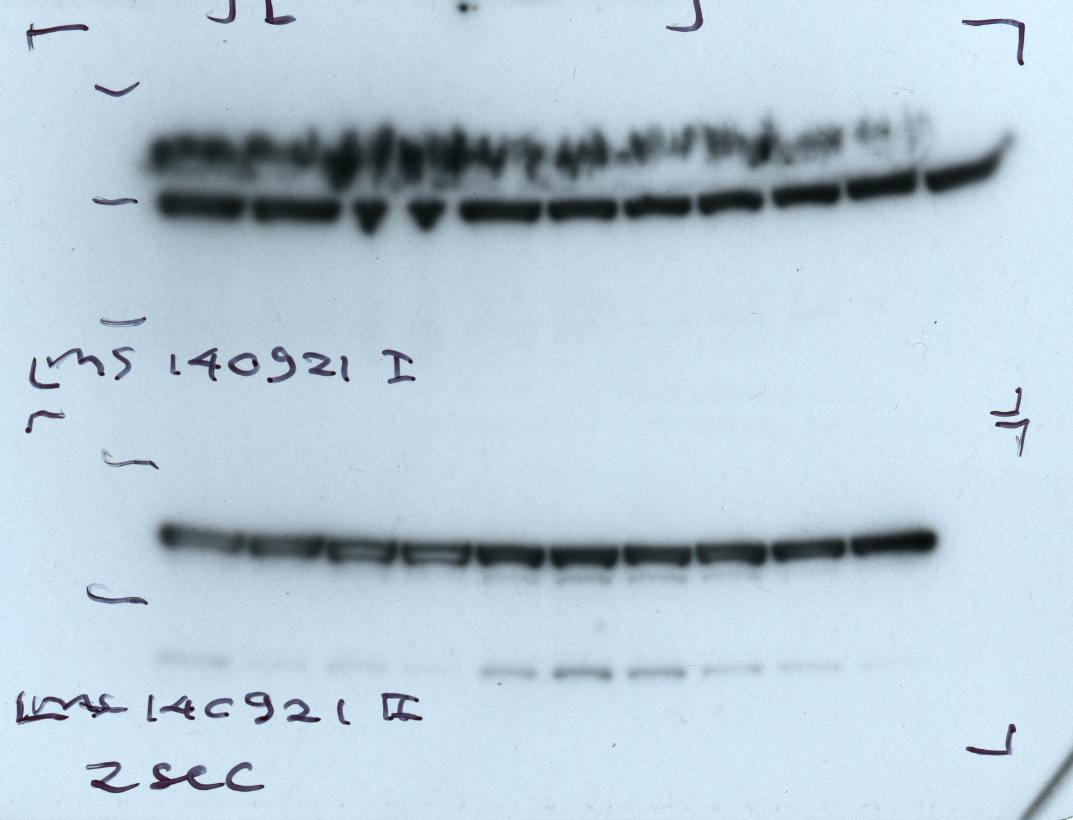

Supplement: Supplementary file 11 — Appendix Figure S2 Source Data [file 44319_2026_727_MOESM11_ESM.zip › Appendix Figure S2 Source data/Panel 2G/ms160921 I and II Actin001 copy.tif]

Appendix Figure S3 Panel A

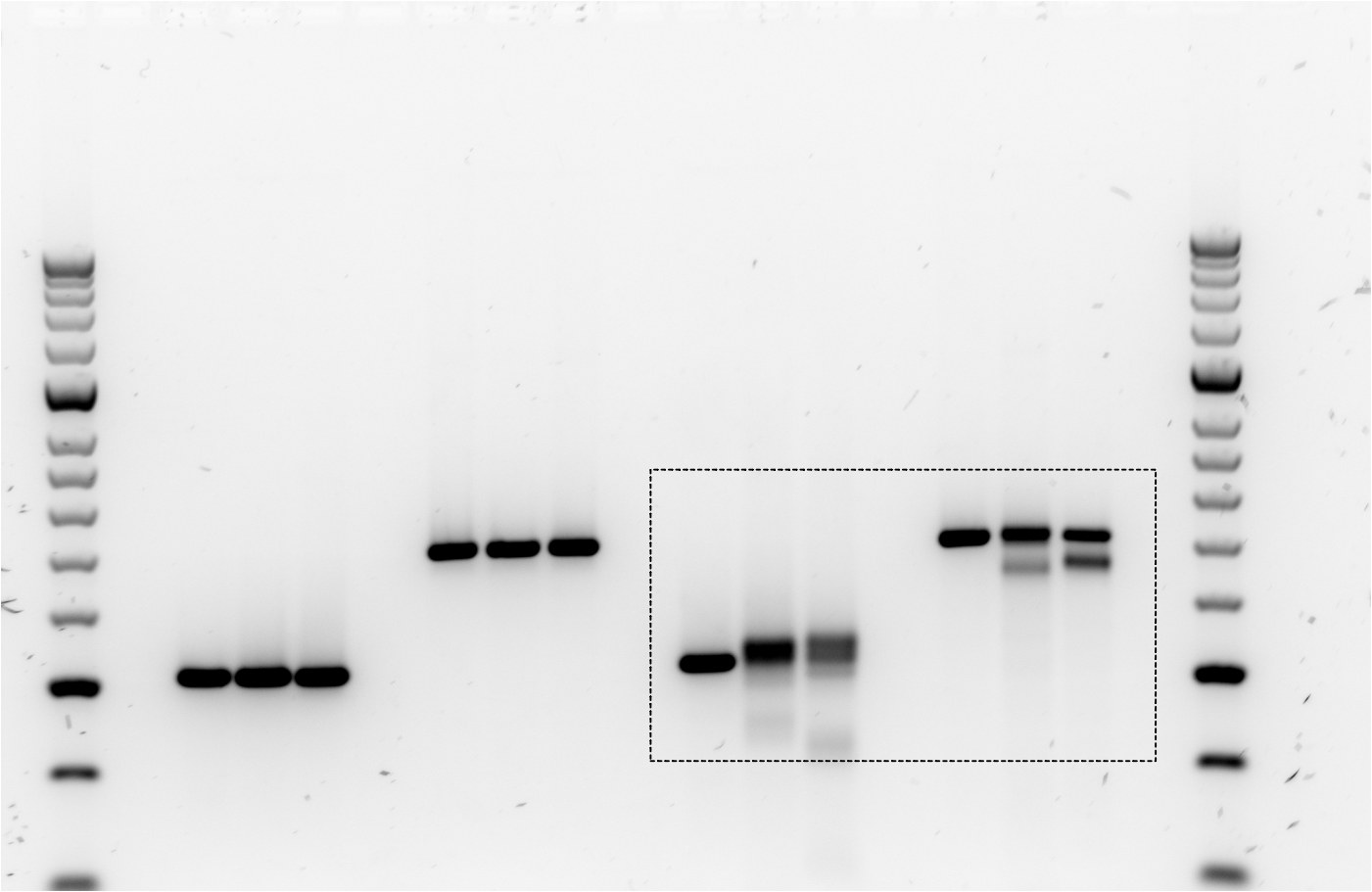

Supplement: Supplementary file 12 — Appendix Figure S3 Source Data [file 44319_2026_727_MOESM12_ESM.zip › Appendix Figure S3 Source data/Appendix Figure S3 cropped area.pdf]

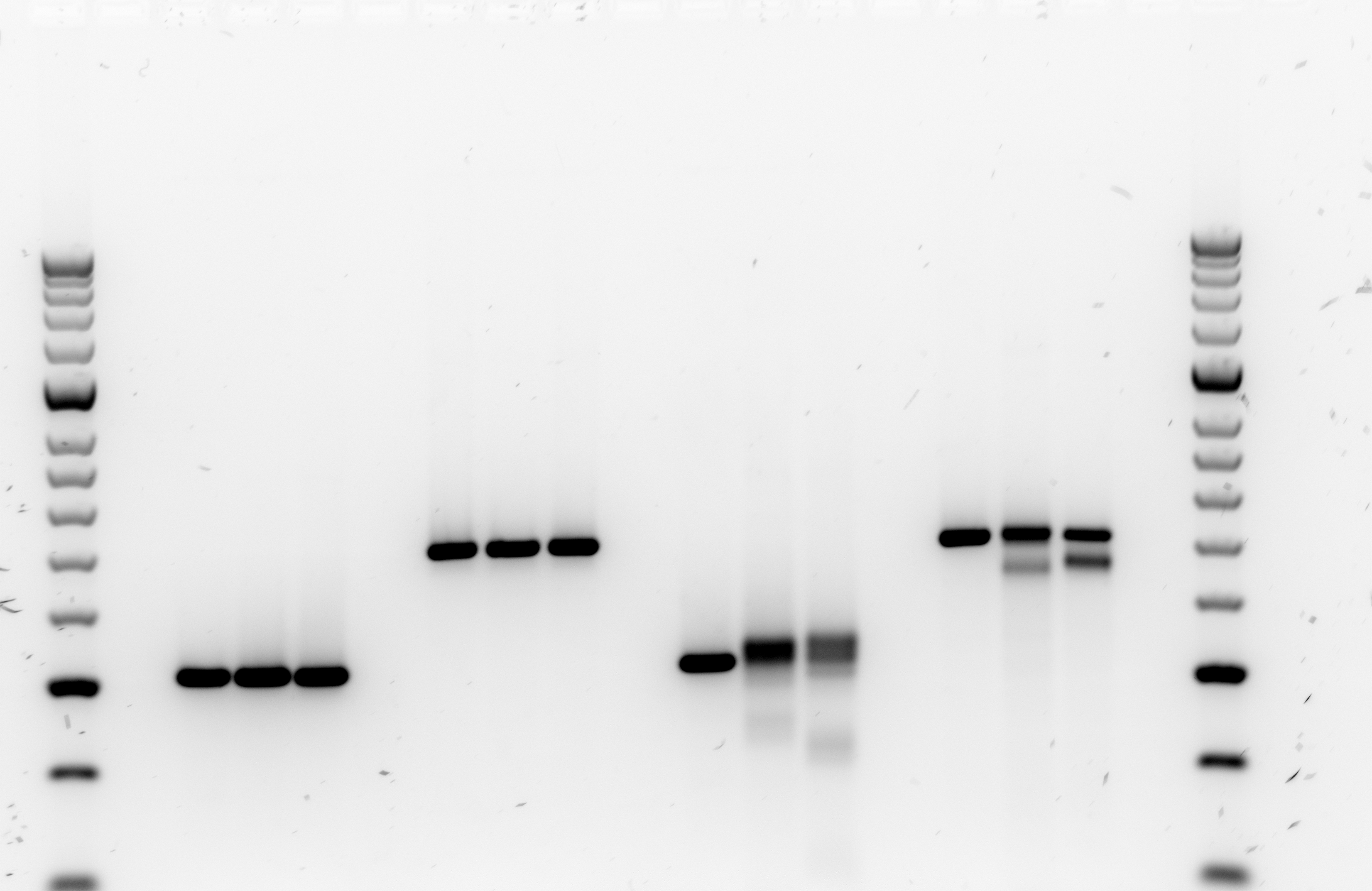

Supplement: Supplementary file 12 — Appendix Figure S3 Source Data [file 44319_2026_727_MOESM12_ESM.zip › Appendix Figure S3 Source data/Panel 3A/msmeets 2020-04-02 17hr 08min T7 endonuclease.tif]
