# Supplementary material for: Minute amounts of helicase-deficient truncated RECQL4 are sufficient for DNA replication
Source: EMBO Rep. 2026 Mar 10;27(7):1759–88. doi: 10.1038/s44319-026-00727-2 (PMC13076768; doi:10.1038/s44319-026-00727-2)
Supplement: Supplementary file 6 — Source data Fig. 2 [file 44319_2026_727_MOESM6_ESM.zip › Figure 2 Source Data/Figure 2 Source data READ ME.docx]

Figure 2 Source data:

Figure 2A. Raw data in Source data

Figure 2B. Raw data in Source data

Figure 2C. Raw data in Source data

Figure 2D. Raw data in Source data
