# Supplementary material for: Minute amounts of helicase-deficient truncated RECQL4 are sufficient for DNA replication
Source: EMBO Rep. 2026 Mar 10;27(7):1759–88. doi: 10.1038/s44319-026-00727-2 (PMC13076768; doi:10.1038/s44319-026-00727-2)
Supplement: Supplementary file 7 — Source data Fig. 3 [file 44319_2026_727_MOESM7_ESM.zip › Figure 3 Source Data/Figure 3 Source data READ ME.docx]

Figure 3 Source data:

Figure 3A. Schematic

Figure 3B. Schematic

Figure 3C. Raw data in Source data

Figure 3D. Raw data in Source data

Figure 3E. Raw data in Source data

Figure 3F. Raw data in Source data

Figure 3G. Raw data in Source data

Figure 3H. Raw data in Source data

Figure 3I. Raw data in Source data
