# Supplementary material for: Minute amounts of helicase-deficient truncated RECQL4 are sufficient for DNA replication
Source: EMBO Rep. 2026 Mar 10;27(7):1759–88. doi: 10.1038/s44319-026-00727-2 (PMC13076768; doi:10.1038/s44319-026-00727-2)
Supplement: Supplementary file 8 — Source data Fig. 4 [file 44319_2026_727_MOESM8_ESM.zip › Figure 4 Source Data/Figure 4 Source data READ ME.docx]

Figure 4 Source data:

Figure 4A. Raw data in Source data

Figure 4B. Schematic

Figure 4C. Raw data in Dataset S2_RECQL4_dropout_CB2_Screen_Results excel sheet

Figure 4D. Raw data in Dataset S2_RECQL4_dropout_CB2_Screen_Results excel sheet

Figure 4E. Schematic and Raw data in Source data
