# Supplementary material for: Minute amounts of helicase-deficient truncated RECQL4 are sufficient for DNA replication
Source: EMBO Rep. 2026 Mar 10;27(7):1759–88. doi: 10.1038/s44319-026-00727-2 (PMC13076768; doi:10.1038/s44319-026-00727-2)
Supplement: Supplementary file 9 — Source data Fig. 5 [file 44319_2026_727_MOESM9_ESM.zip › Figure 5 Source Data/Figure 5 Source data READ ME.docx]

Figure 5 Source data:

Figure 5A. Schematic

Figure 5B. Raw data in Source data

Figure 5C. Raw data in Source data

Figure 5D. Raw data in Source data

Figure 5E. Raw data in Source data
