# Supplementary material for: Minute amounts of helicase-deficient truncated RECQL4 are sufficient for DNA replication
Source: EMBO Rep. 2026 Mar 10;27(7):1759–88. doi: 10.1038/s44319-026-00727-2 (PMC13076768; doi:10.1038/s44319-026-00727-2)
Supplement: Supplementary file 9 — Source data Fig. 5 [file 44319_2026_727_MOESM9_ESM.zip › Figure 5 Source Data/Figure 5B/CHEMI_07052024_132749_10s.pdf]

iBright™ Imager Analysis Report

Date:	2024/05/07 01:27:49 PM
Mode:	Chemi Blots
Notes:	No Comments
Model:	iBright™ CL750
Inst Name:	2462423040007
Serial No:	2462423040007
Version:	1.8.0
User:	WALKLEY
Exposure Time:	10000 ms
Exposure Mode:	Normal
Image area:	225.40mm x 180.32mm
Image size	1408px x 1127px
Optical Zoom:	1x
Digital Zoom:	1.2x
Focus level:	200
Dye:	Chemi
Excitation:	None
Emission:	None
Resolution:	2 x 2
Sensitivity:	100%

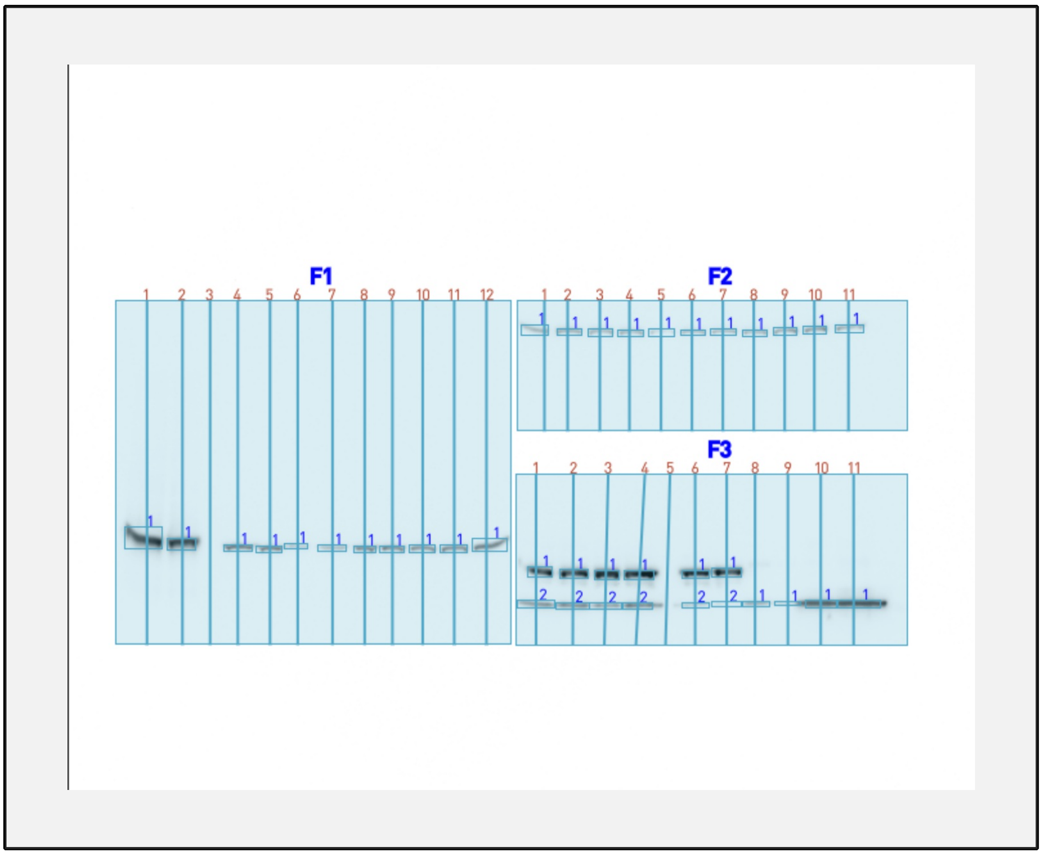

CHEMI_07052024_132749_10s_(Chemi).tif

CHEMI_07052024_132749_10s_(Chemi).tif
LANE AND BAND ANALYSIS DATA TABLE

Frame 1 Channel 1
Lane 1

Band ID	Volume	Area	Local Bg. Corr. Vol.	Rf	Density	Band Purity %	Lane Purity %
1	46910000	2220	36731100	0.688	21130	100	109.231

Lane 2

Band ID	Volume	Area	Local Bg. Corr. Vol.	Rf	Density	Band Purity %	Lane Purity %
1	31632600	987	23649000	0.705	32049	100	81.762

Lane 4

Band ID	Volume	Area	Local Bg. Corr. Vol.	Rf	Density	Band Purity %	Lane Purity %
1	10461500	598	8833940	0.716	17494	100	91.238

Lane 5

Band ID	Volume	Area	Local Bg. Corr. Vol.	Rf	Density	Band Purity %	Lane Purity %
1	8185530	546	6778610	0.720	14991	100	84.709

Lane 6

Band ID	Volume	Area	Local Bg. Corr. Vol.	Rf	Density	Band Purity %	Lane Purity %
1	2511830	468	1839330	0.711	5367	100	75.436

Lane 7

Band ID	Volume	Area	Local Bg. Corr. Vol.	Rf	Density	Band Purity %	Lane Purity %
1	4045110	552	3429120	0.716	7328	100	83.830

Lane 8

Band ID	Volume	Area	Local Bg. Corr. Vol.	Rf	Density	Band Purity %	Lane Purity %
1	7185220	532	6079140	0.720	13506	100	83.031

Lane 9

Band ID	Volume	Area	Local Bg. Corr. Vol.	Rf	Density	Band Purity %	Lane Purity %
1	7393850	520	6169620	0.720	14218	100	81.494

Lane 10

Band ID	Volume	Area	Local Bg. Corr. Vol.	Rf	Density	Band Purity %	Lane Purity %
1	7578480	645	6569370	0.720	11749	100	86.777

Lane 11

Band ID	Volume	Area	Local Bg. Corr. Vol.	Rf	Density	Band Purity %	Lane Purity %
1	8667880	675	7575480	0.720	12841	100	89.180

Lane 12

Band ID	Volume	Area	Local Bg. Corr. Vol.	Rf	Density	Band Purity %	Lane Purity %
1	13382300	1254	11939300	0.707	10671	100	112.078

Frame 2 Channel 1

Lane 1

Band ID	Volume	Area	Local Bg. Corr. Vol.	Rf	Density	Band Purity %	Lane Purity %
1	5175300	817	4492800	0.221	6334	100	121.425

Lane 2

Band ID	Volume	Area	Local Bg. Corr. Vol.	Rf	Density	Band Purity %	Lane Purity %
1	5134400	574	4309090	0.235	8944	100	89.480

Lane 3

Band ID	Volume	Area	Local Bg. Corr. Vol.	Rf	Density	Band Purity %	Lane Purity %
1	4305350	588	3704170	0.240	7322	100	89.354

Lane 4

Band ID	Volume	Area	Local Bg. Corr. Vol.	Rf	Density	Band Purity %	Lane Purity %
1	4158520	546	3604710	0.245	7616	100	88.597

Lane 5

Band ID	Volume	Area	Local Bg. Corr. Vol.	Rf	Density	Band Purity %	Lane Purity %
1	1568590	616	1336930	0.240	2546	100	90.229

Lane 6

Band ID	Volume	Area	Local Bg. Corr. Vol.	Rf	Density	Band Purity %	Lane Purity %
1	3228460	492	2833730	0.240	6561	100	86.953

Lane 7

Band ID	Volume	Area	Local Bg. Corr. Vol.	Rf	Density	Band Purity %	Lane Purity %
1	3512780	516	3082500	0.235	6807	100	89.941

Lane 8

Band ID	Volume	Area	Local Bg. Corr. Vol.	Rf	Density	Band Purity %	Lane Purity %
1	3697380	533	3267420	0.245	6936	100	88.967

Lane 9

Band ID	Volume	Area	Local Bg. Corr. Vol.	Rf	Density	Band Purity %	Lane Purity %
1	5229190	600	4653740	0.235	8715	100	91.573

Lane 10

Band ID	Volume	Area	Local Bg. Corr. Vol.	Rf	Density	Band Purity %	Lane Purity %
1	5728150	546	5121120	0.221	10491	100	89.290

Lane 11

Band ID	Volume	Area	Local Bg. Corr. Vol.	Rf	Density	Band Purity %	Lane Purity %
1	4883000	720	4329830	0.211	6781	100	106.824

Frame 3 Channel 1

Lane 1

Band ID	Volume	Area	Local Bg. Corr. Vol.	Rf	Density	Band Purity %	Lane Purity %
1	20929800	798	17493600	0.571	26227	67.522	61.638
2	10459200	885	8414570	0.757	11818	32.478	30.802

Lane 2

Band ID	Volume	Area	Local Bg. Corr. Vol.	Rf	Density	Band Purity %	Lane Purity %
1	25152700	799	20089500	0.582	31480	70.590	60.601
2	10696500	715	8369830	0.765	14960	29.410	25.771

Lane 3

Band ID	Volume	Area	Local Bg. Corr. Vol.	Rf	Density	Band Purity %	Lane Purity %
1	27466500	738	21921300	0.586	37217	82.330	64.800
2	7014740	624	4704900	0.765	11241	17.670	16.550

Lane 4

Band ID	Volume	Area	Local Bg. Corr. Vol.	Rf	Density	Band Purity %	Lane Purity %
1	31817100	784	24070800	0.586	40583	75.066	64.023
2	10535000	588	7995300	0.765	17916	24.934	21.199

Lane 6

Band ID	Volume	Area	Local Bg. Corr. Vol.	Rf	Density	Band Purity %	Lane Purity %
1	24160600	675	17599900	0.582	35793	89.871	71.214
2	2559690	495	1983730	0.761	5171	10.130	7.545

Lane 7

Band ID	Volume	Area	Local Bg. Corr. Vol.	Rf	Density	Band Purity %	Lane Purity %
1	24299700	816	19441500	0.575	29779	97.508	83.256
2	1081590	480	496811	0.754	2253	2.492	3.706

Lane 8

Band ID	Volume	Area	Local Bg. Corr. Vol.	Rf	Density	Band Purity %	Lane Purity %
1	4483980	495	3754290	0.754	9058	100	59.526

Lane 9

Band ID	Volume	Area	Local Bg. Corr. Vol.	Rf	Density	Band Purity %	Lane Purity %
1	3573200	344	1919380	0.750	10387	100	55.663

Lane 10

Band ID	Volume	Area	Local Bg. Corr. Vol.	Rf	Density	Band Purity %	Lane Purity %
1	21205800	728	13609100	0.754	29128	100	87.275

Lane 11

Band ID	Volume	Area	Local Bg. Corr. Vol.	Rf	Density	Band Purity %	Lane Purity %
1	31192800	966	20677400	0.754	32290	100	109.074
