# Supplementary material for: Minute amounts of helicase-deficient truncated RECQL4 are sufficient for DNA replication
Source: EMBO Rep. 2026 Mar 10;27(7):1759–88. doi: 10.1038/s44319-026-00727-2 (PMC13076768; doi:10.1038/s44319-026-00727-2)
Supplement: Supplementary file 10 — Source data Fig. 6 [file 44319_2026_727_MOESM10_ESM.zip › Figure 6 Source Data/Figure 6 Source data READ ME.docx]

Figure 6 Source data:

Figure 6A-6D all data underlying these figures is contained in

**Appendix Dataset S3**. Whole Genome sequencing dataset used to calculate replication timing: GSE272599 (NCBI GEO dataset)

| [GSM8406471](https://www.ncbi.nlm.nih.gov/geo/query/acc.cgi?acc=GSM8406471) | Immortalized myeloid cell line, WT replicate 1 |
| --- | --- |
| [GSM8406472](https://www.ncbi.nlm.nih.gov/geo/query/acc.cgi?acc=GSM8406472) | Immortalized myeloid cell line, WT replicate 2 |
| [GSM8406473](https://www.ncbi.nlm.nih.gov/geo/query/acc.cgi?acc=GSM8406473) | Immortalized myeloid cell line, WT replicate 3 |

| [GSM8406474](https://www.ncbi.nlm.nih.gov/geo/query/acc.cgi?acc=GSM8406474) | Immortalized myeloid cell line, Recql4 -/- Klhdc3 -/- replicate 1 |
| --- | --- |
| [GSM8406475](https://www.ncbi.nlm.nih.gov/geo/query/acc.cgi?acc=GSM8406475) | Immortalized myeloid cell line, Recql4 -/- Klhdc3 -/- replicate 2 |
| [GSM8406476](https://www.ncbi.nlm.nih.gov/geo/query/acc.cgi?acc=GSM8406476) | Immortalized myeloid cell line, Recql4 -/- Klhdc3 -/- replicate 3 |

Figure 6E. Schematic
