# Supplementary material for: Minute amounts of helicase-deficient truncated RECQL4 are sufficient for DNA replication
Source: EMBO Rep. 2026 Mar 10;27(7):1759–88. doi: 10.1038/s44319-026-00727-2 (PMC13076768; doi:10.1038/s44319-026-00727-2)

Appendix Figure S2 Panel E

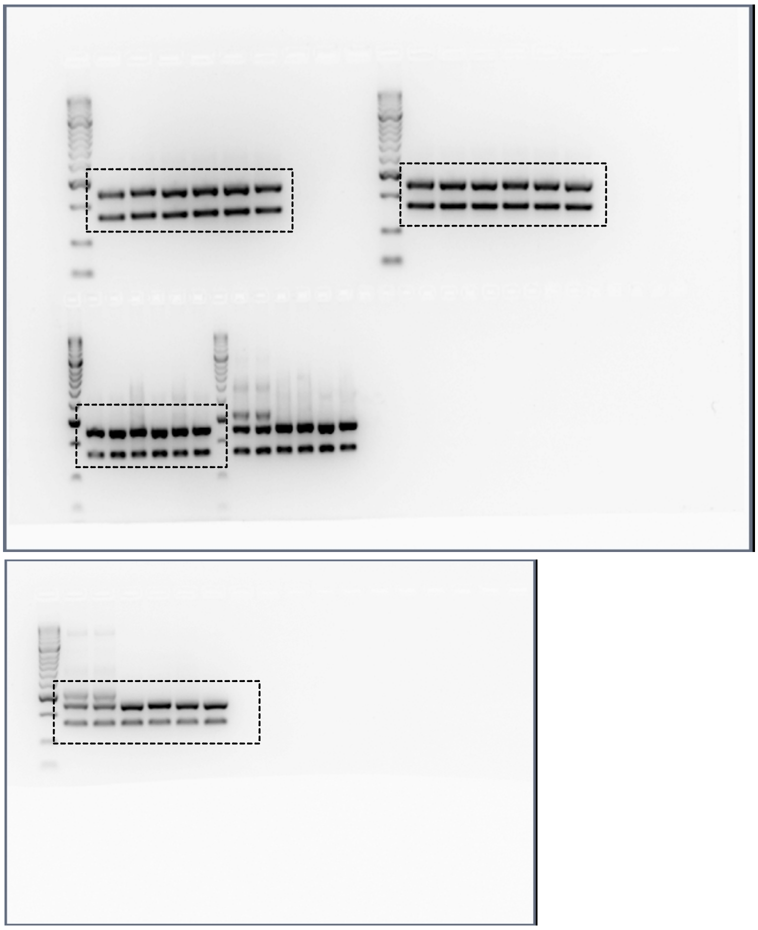

Appendix Figure S2 Panel F

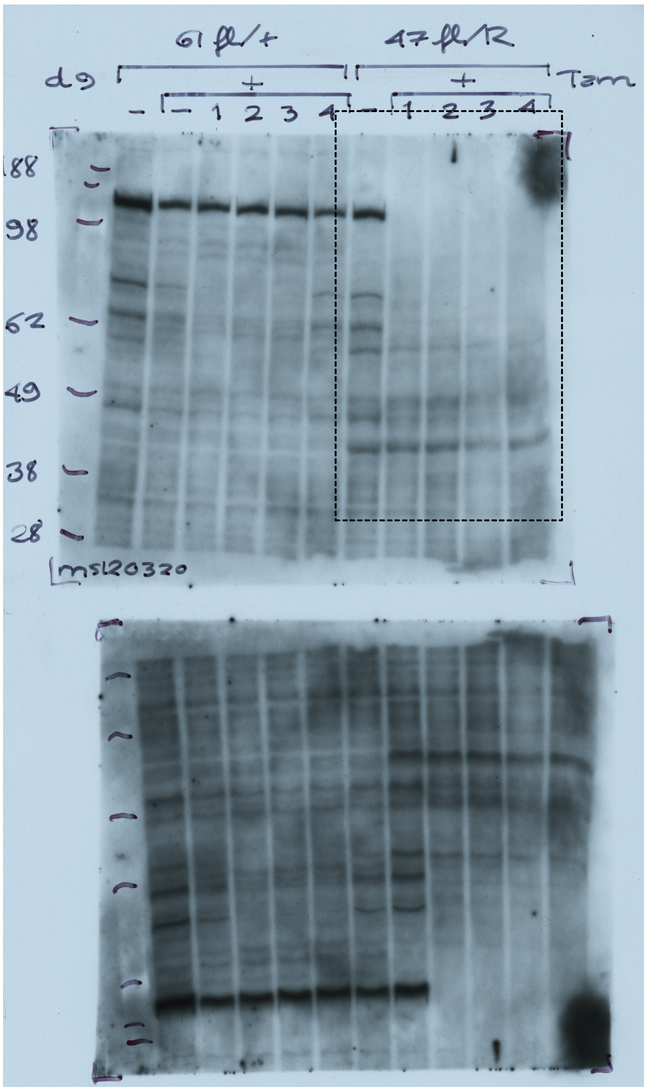

Appendix Figure S2 Panel G (Recql4)

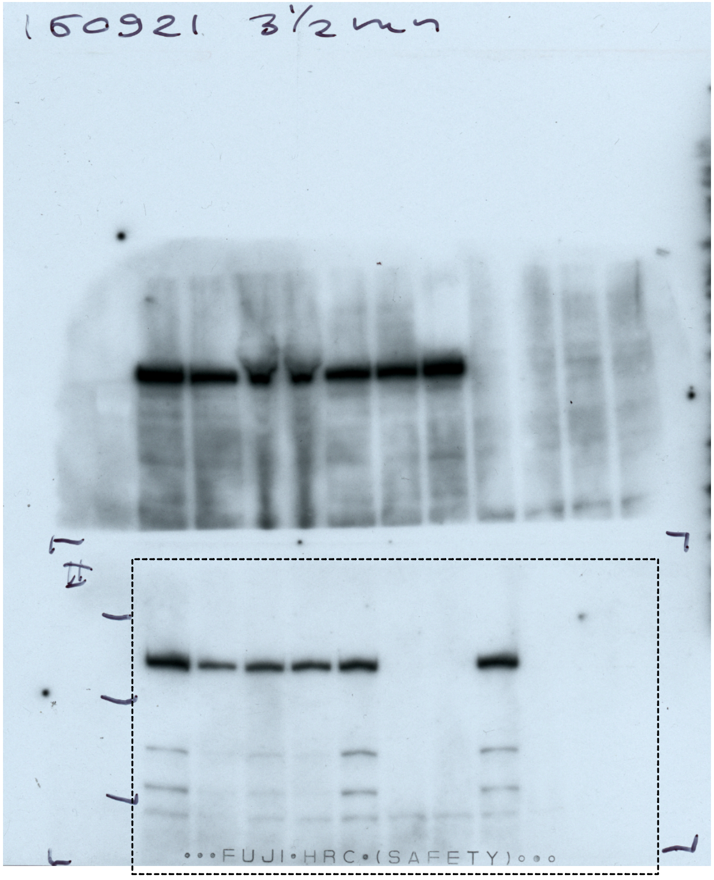

Appendix Figure S2 Panel G (Actin)

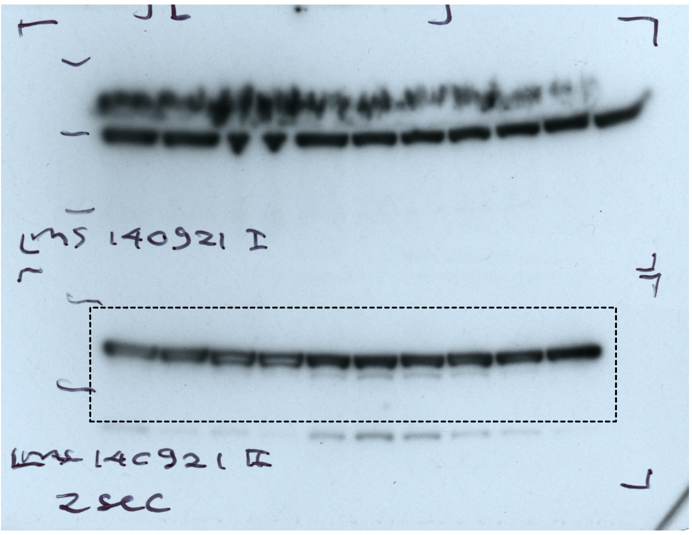

Supplement: Supplementary file 11 — Appendix Figure S2 Source Data [file 44319_2026_727_MOESM11_ESM.zip › Appendix Figure S2 Source data/Appendix Figure S2 Cropped areas panels 2E-2G.pdf]
