# Supplementary material for: Minute amounts of helicase-deficient truncated RECQL4 are sufficient for DNA replication
Source: EMBO Rep. 2026 Mar 10;27(7):1759–88. doi: 10.1038/s44319-026-00727-2 (PMC13076768; doi:10.1038/s44319-026-00727-2)

15.09.21
Full membrane (ms140921 II) probed
1st time with anti-RECQL4 (ms150921).

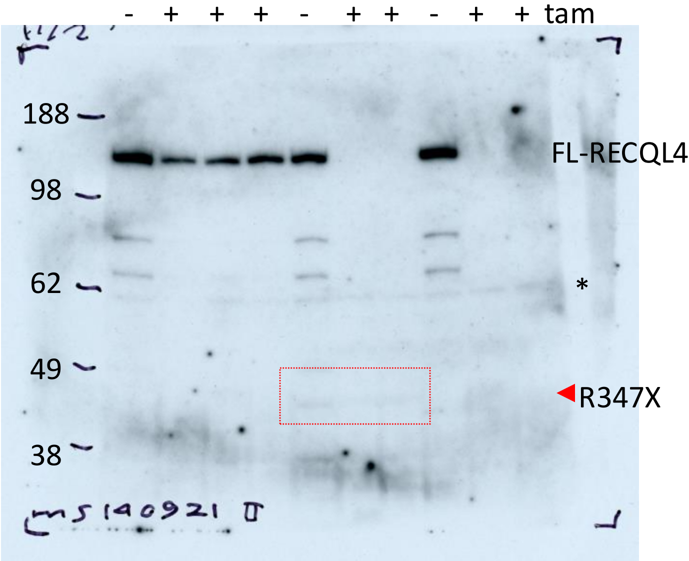

R347X bands are very faint on this blot.
You used a cropped version of the blot on the right for the DOD grant to show absence of full-length RECQL4.

- Background band

16.09.21
Membrane ms140921 II re-probed with:
Top: anti-RECQL4
Bottom: anti-Actin (ms160921).

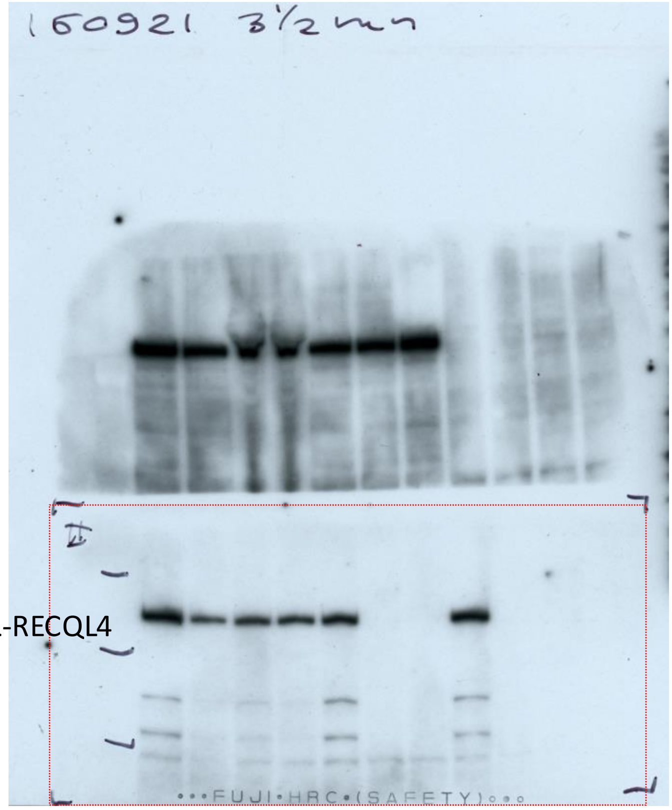

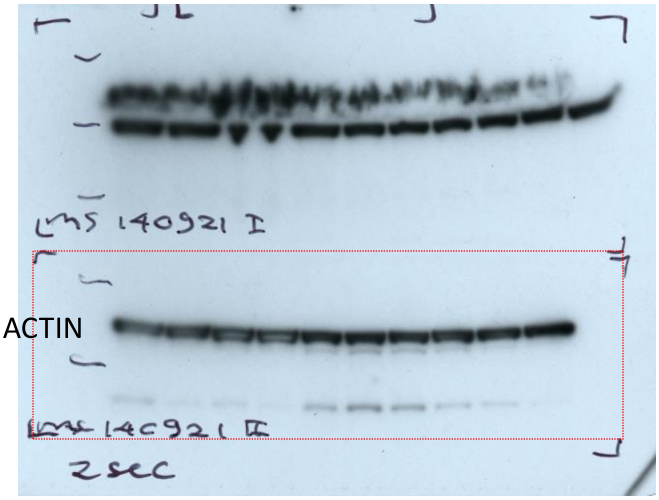

Supplement: Supplementary file 11 — Appendix Figure S2 Source Data [file 44319_2026_727_MOESM11_ESM.zip › Appendix Figure S2 Source data/Panel 2G/WB Klhdc3 validation Exp3.pdf]
