# Supplementary material for: Minute amounts of helicase-deficient truncated RECQL4 are sufficient for DNA replication
Source: EMBO Rep. 2026 Mar 10;27(7):1759–88. doi: 10.1038/s44319-026-00727-2 (PMC13076768; doi:10.1038/s44319-026-00727-2)
Supplement: Supplementary file 13 — Expanded View Figures [file 44319_2026_727_MOESM13_ESM.pdf]

Expanded View Figures

Figure EV1. Cell cycle kinetics of WT, point mutant and *Recql4*-deficient myeloid cells.

(**A**) Representative flow cytometry plots assessing DNA replication rates following pulse labelling of asynchronous cultures of myeloid cells with EdU incorporation and DAPI (DNA stain). Genotypes assessed as *Recql4* wild-type (WT), *Recql4*$^{fl/G522Efs}$, *Recql4*$^{fl/R347X}$ and *Recql4*$^{fl/fl}$. All cell lines are *R26*-CreER$^{T2ki/+}$ and *Klhdc3* wild-type. Day after tamoxifen addition as indicated. (**B**) Genomic PCR showing recombination of the floxed *Recql4* allele at the indicated days after the addition of tamoxifen in each respective genotype. All cells are *Klhdc3*$^{+/+}$. (**C**) Schematic of the predicted protein products from the different Recql4 alleles used in this study.

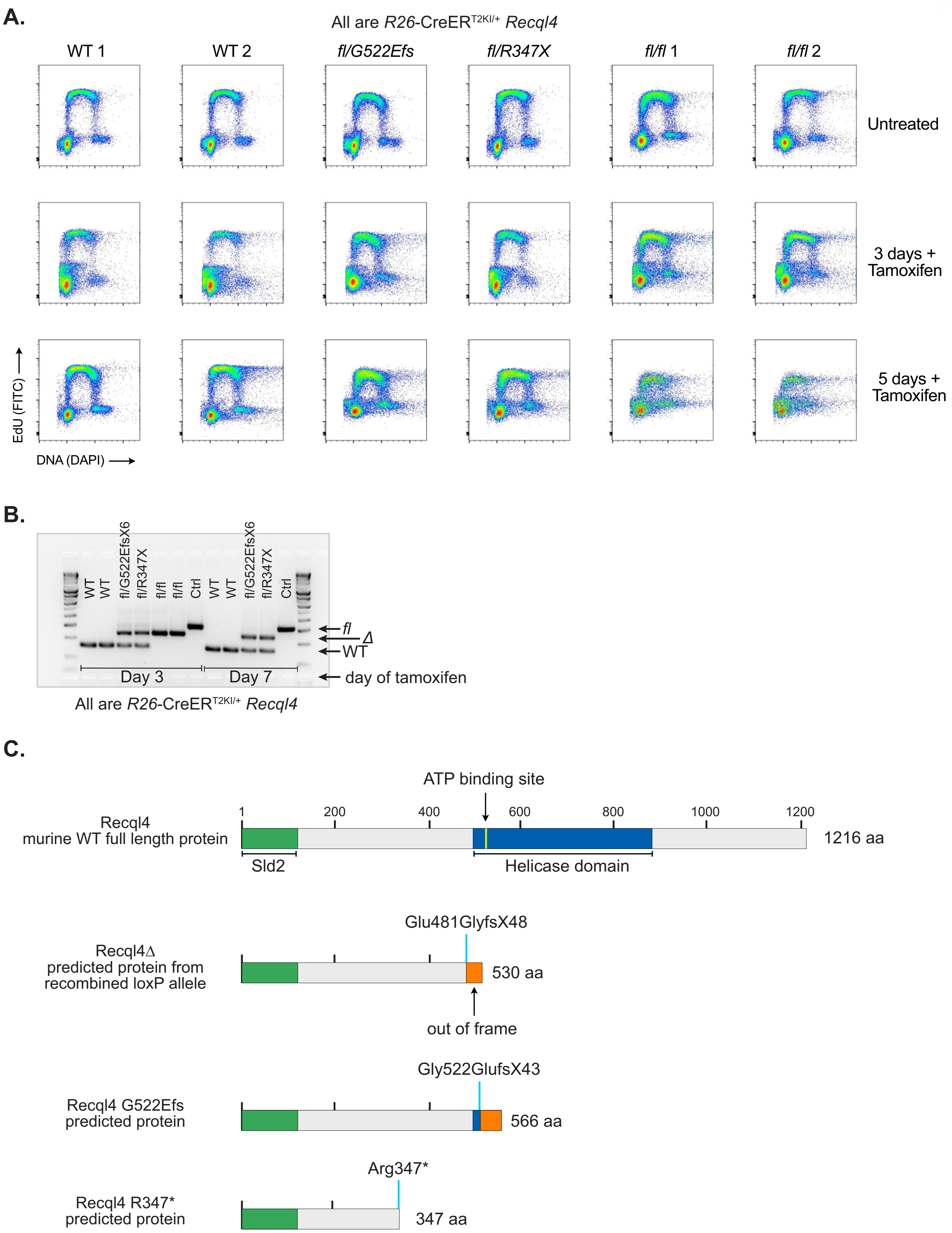
A.
All are R26-CreER T2KI/+ Recql4
WT 1
WT 2
fl/G522Efs
fl/R347X
fl/fl 1
fl/fl 2
Untreated
3 days + Tamoxifen
5 days + Tamoxifen
EdU (FITC)
DNA (DAPI)
B.
WT
WT
fl/G522EfsX6
fl/R347X
fl/fl
fl/fl
Ctrl
WT
WT
fl/G522EfsX6
fl/R347X
Ctrl
fl
Δ
WT
Day 3
Day 7
day of tamoxifen
All are R26-CreER T2KI/+ Recql4
C.
ATP binding site
1
200
400
600
800
1000
1200
Recql4
murine WT full length protein
1216 aa
Sld2
Helicase domain
Glu481GlyfsX48
Recql4Δ
predicted protein from
recombined loxP allele
530 aa
out of frame
Gly522GlufsX43
Recql4 G522Efs
predicted protein
566 aa
Arg347*
Recql4 R347*
predicted protein
347 aa

 © The Author(s)

A.

E12.5 *Recql4*$^{+/-}$*Klhdc3*$^{+/-}$ inbreeding

Mouse #	No. of Embryos	$R^{+/+}K^{+/+}$	$R^{+/+}K^{+/-}$	$R^{+/+}K^{-/-}$	$R^{+/-}K^{+/+}$	$R^{+/-}K^{+/-}$	$R^{+/-}K^{-/-}$	$R^{-/-}K^{-/-}$
F120	7	1	0	0	0	5	1	0
F128	6	0	3	0	1	0	0	2
F125	8	3	1	1	1	1	1	0
F147	6	0	1	2	0	2	1	0
F135	5	2	1	1	0	1	0	0
F182	7	0	2	1	1	2	1	0
F183	5	0	1	1	0	2	0	1
**Total**	**44**	**6**	**9**	**6**	**3**	**13**	**4**	**3**
		**13.64%**	**20.45%**	**13.64%**	**6.82%**	**29.55%**	**9.09%**	**6.82%**
Expected		2.75	5.5	2.75	5.5	11	5.5	2.75
		6.25%	12.50%	6.25%	12.50%	25.00%	12.50%	6.25%

B.

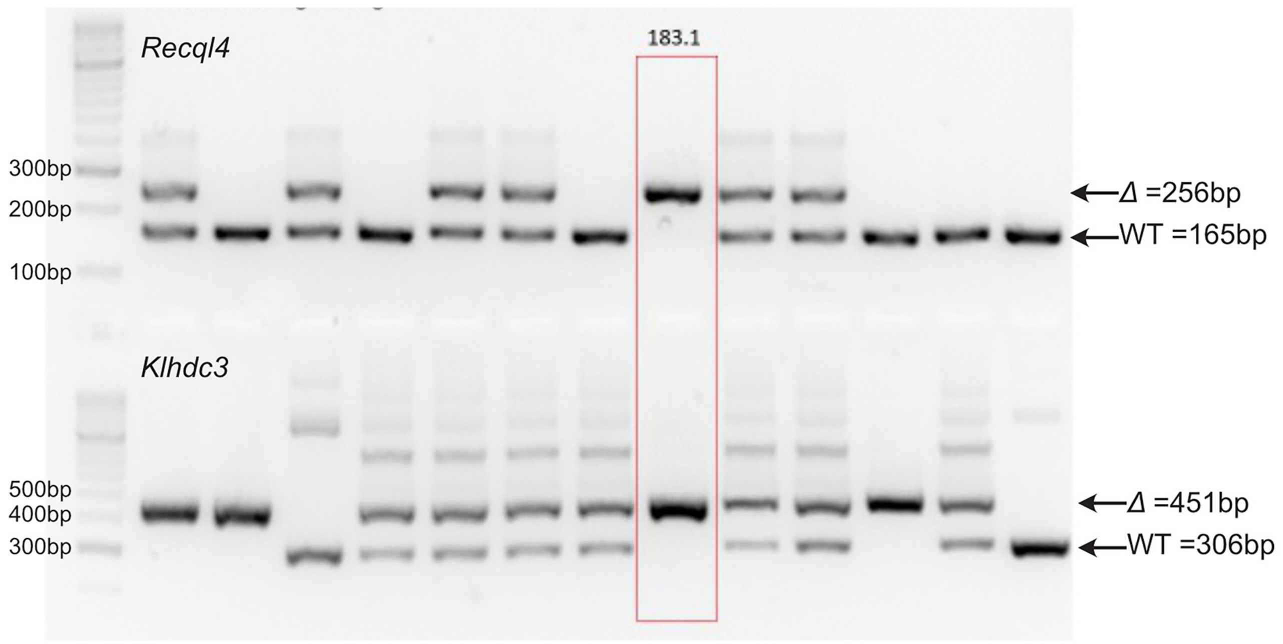

Each vertical lane represents DNA genotyping from an individual embryo

Figure EV2. Loss of *Klhdc3* extends the survival of *Recql4* deficient embryos in vivo.

(**A**) Recovery of indicated genotypes at embryonic day 12.5 (E12.5) from inbreeding of *Recql4*$^{+/-}$*Klhdc3*$^{+/-}$ breeding pairs. Previous analysis demonstrated that the *Recql4*$^{-/-}$ embryos were lethal prior to E10.5 (specific time point prior to this not determined). (**B**) Genomic PCR showing demonstrating recovery of a homozygous embryo.

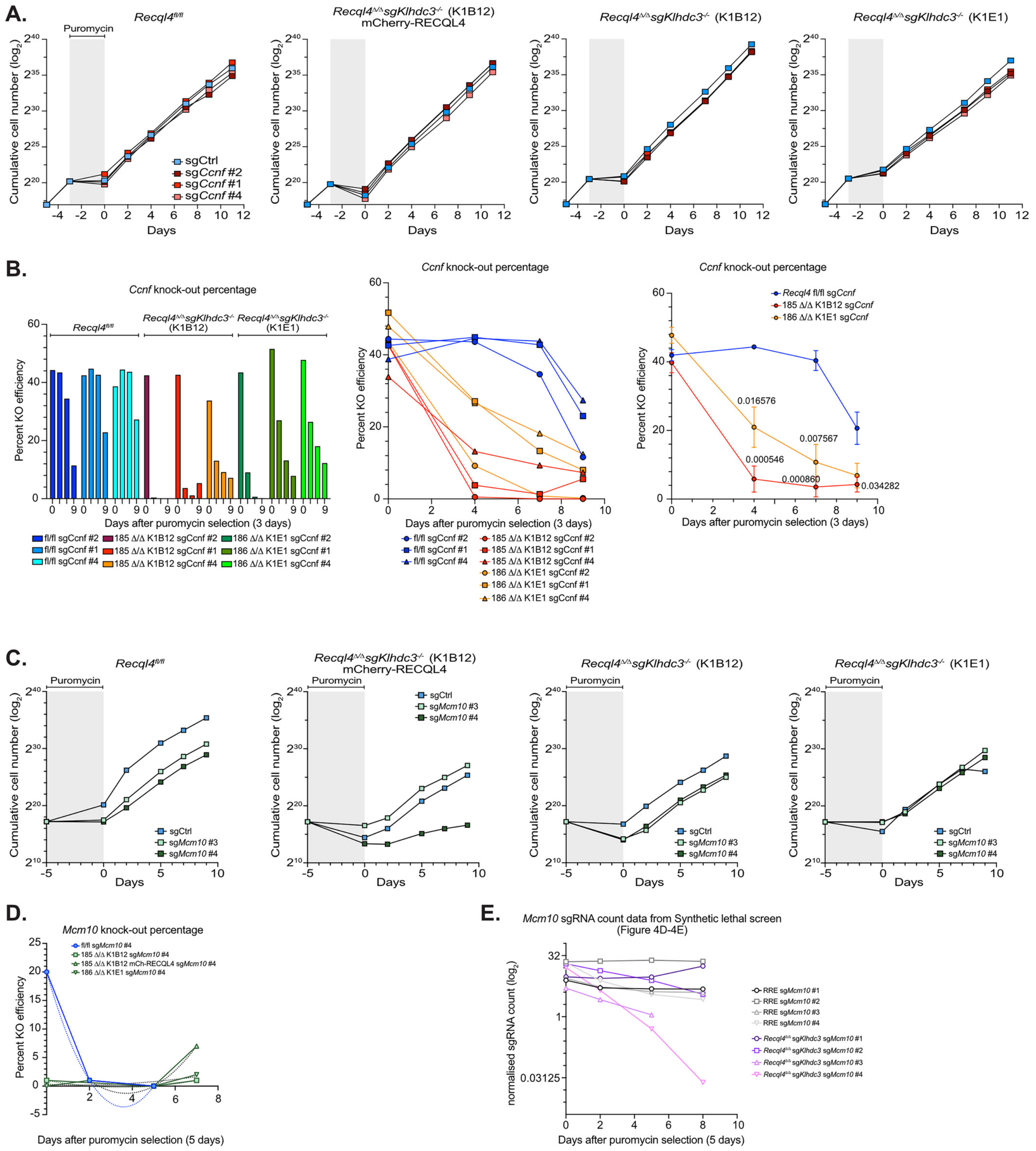
A.
Recql4fl/fl
Puromycin
Cumulative cell number (log2)
sgCtrl
sgCcnf #2
sgCcnf #1
sgCcnf #4
Days
Recql4Δ/ΔsgKlhdc3-/- (K1B12)
mCherry-RECQL4
Recql4Δ/ΔsgKlhdc3-/- (K1B12)
Recql4Δ/ΔsgKlhdc3-/- (K1E1)
B.
Ccnf knock-out percentage
Recql4fl/fl
Recql4Δ/ΔsgKlhdc3-/- (K1B12)
Recql4Δ/ΔsgKlhdc3-/- (K1E1)
Percent KO efficiency
Days after puromycin selection (3 days)
fl/fl sgCcnf #2
fl/fl sgCcnf #1
fl/fl sgCcnf #4
185 Δ/Δ K1B12 sgCcnf #2
185 Δ/Δ K1B12 sgCcnf #1
185 Δ/Δ K1B12 sgCcnf #4
186 Δ/Δ K1E1 sgCcnf #2
186 Δ/Δ K1E1 sgCcnf #1
186 Δ/Δ K1E1 sgCcnf #4
Recql4 fl/fl sgCcnf
185 Δ/Δ K1B12 sgCcnf
186 Δ/Δ K1E1 sgCcnf
0.016576
0.007567
0.000546
0.000860
0.034282
C.
sgCtrl
sgMcm10 #3
sgMcm10 #4
D.
Mcm10 knock-out percentage
fl/fl sgMcm10 #4
185 Δ/Δ K1B12 sgMcm10 #4
185 Δ/Δ K1B12 mCh-RECQL4 sgMcm10 #4
186 Δ/Δ K1E1 sgMcm10 #4
Days after puromycin selection (5 days)
E.
Mcm10 sgRNA count data from Synthetic lethal screen
(Figure 4D-4E)
normalised sgRNA count (log2)
RRE sgMcm10 #1
RRE sgMcm10 #2
RRE sgMcm10 #3
RRE sgMcm10 #4
Recql4Δ/Δ sgKlhdc3 sgMcm10 #1
Recql4Δ/Δ sgKlhdc3 sgMcm10 #2
Recql4Δ/Δ sgKlhdc3 sgMcm10 #3
Recql4Δ/Δ sgKlhdc3 sgMcm10 #4
Days after puromycin selection (5 days)

 © The Author(s)

Figure EV3. Validation that loss of Cyclin F is synthetic lethal with *Recql4* deficiency.

(**A**) Proliferation curves of sgCtrl and sg*Ccnf*-targeted cell lines of the indicated genotypes. Grey shaded area indicates puromycin selection. *Recql4*$^{\Delta/\Delta}$ sg*Klhdc3* K1B12 and *Recql4*$^{\Delta/\Delta}$ sg*Klhdc3* K1E1 are independently targeted and isolated clones. (**B**) The knockout efficiency of *Ccnf* was measured through Sanger Sequencing and analysed by TIDE at each time point in each genotype as indicated. Data shown as each sample individually and as the mean knockout efficiency of 3 independent sg*Ccnf* guides +/- SEM (right panel). Statistical analysis was done using multiple unpaired t-tests with significant p-values listed based on the nonlinear fit model of the data. (**C**) Proliferation curves of sgCtrl and sg*Mcm10*-targeted cell lines of the indicated genotypes. Grey shaded area indicates puromycin selection. *Recql4*$^{\Delta/\Delta}$ sg*Klhdc3* K1B12 and *Recql4*$^{\Delta/\Delta}$ sg*Klhdc3* K1E1 are independently targeted and isolated clones. (**D**) The knockout efficiency of *Mcm10* was measured through Sanger Sequencing and analysed by Synthego at each time point in each genotype as indicated. Data shown as each sample individually. (**E**) sgRNA counts from the synthetic lethal screen (Fig. 4) for *Mcm10* targeting sgRNAs in the *Recql4*$^{\Delta/\Delta}$ sg*Klhdc3* cells (labelled as DKO – double knock-out) and control *Recql4*$^{\Delta/\Delta}$ sg*Klhdc3* reconstituted with mCherry-RECQL4 (labelled as RRE – RECQL4 re-expressed).

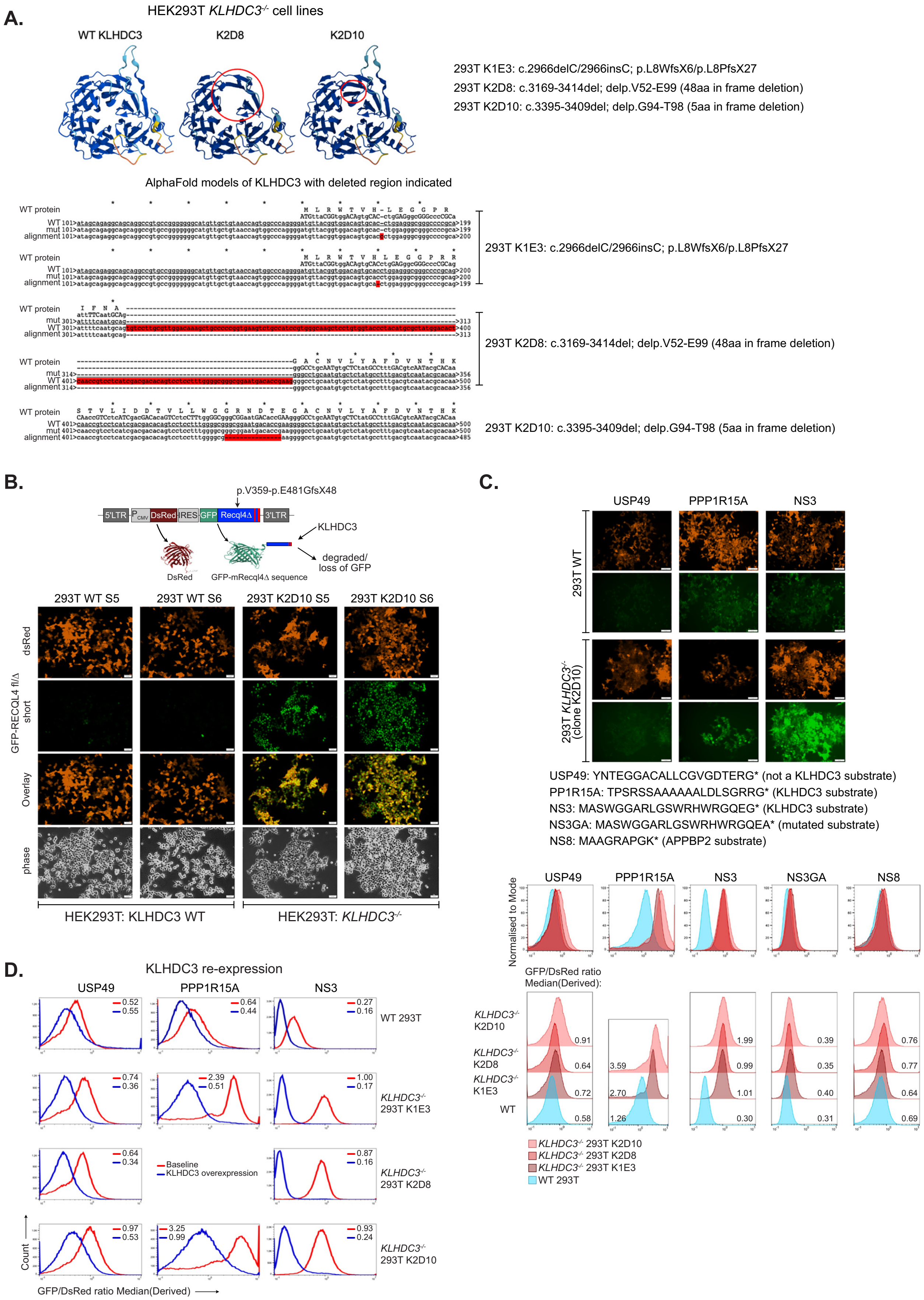
A.
HEK293T KLHDC3-/- cell lines
WT KLHDC3
K2D8
K2D10
293T K1E3: c.2966delC/2966insC; p.L8WfsX6/p.L8PfsX27
293T K2D8: c.3169-3414del; delp.V52-E99 (48aa in frame deletion)
293T K2D10: c.3395-3409del; delp.G94-T98 (5aa in frame deletion)
AlphaFold models of KLHDC3 with deleted region indicated
WT protein
WT
mut
alignment
293T K1E3: c.2966delC/2966insC; p.L8WfsX6/p.L8PfsX27
293T K2D8: c.3169-3414del; delp.V52-E99 (48aa in frame deletion)
293T K2D10: c.3395-3409del; delp.G94-T98 (5aa in frame deletion)
B.
p.V359-p.E481GfsX48
5'LTR
DsRed
IRES
GFP
Recql4Δ
3'LTR
KLHDC3
degraded/ loss of GFP
DsRed
GFP-mRecql4Δ sequence
293T WT S5
293T WT S6
293T K2D10 S5
293T K2D10 S6
dsRed
GFP-RECQL4 fl/Δ short
Overlay
phase
HEK293T: KLHDC3 WT
HEK293T: KLHDC3-/-
C.
USP49
PPP1R15A
NS3
293T WT
293T KLHDC3-/- (clone K2D10)
USP49: YNTEGGACALLCGVGDTERG* (not a KLHDC3 substrate)
PP1R15A: TPSRSSAAAAAALDLSGRRG* (KLHDC3 substrate)
NS3: MASWGGARLGSWRHWRGQEG* (KLHDC3 substrate)
NS3GA: MASWGGARLGSWRHWRGQEA* (mutated substrate)
NS8: MAAGRAPGK* (APPBP2 substrate)
USP49
PPP1R15A
NS3
NS3GA
NS8
Normalised to Mode
GFP/DsRed ratio Median(Derived):
KLHDC3-/- K2D10
KLHDC3-/- K2D8
KLHDC3-/- K1E3
WT
0.91
0.64
0.72
0.58
3.59
2.70
1.26
1.99
0.99
1.01
0.30
0.39
0.35
0.40
0.31
0.76
0.77
0.64
0.69
KLHDC3-/- 293T K2D10
KLHDC3-/- 293T K2D8
KLHDC3-/- 293T K1E3
WT 293T
D.
KLHDC3 re-expression
USP49
PPP1R15A
NS3
0.52
0.55
0.64
0.44
0.27
0.16
WT 293T
0.74
0.36
2.39
0.51
1.00
0.17
KLHDC3-/- 293T K1E3
0.64
0.34
Baseline
KLHDC3 overexpression
0.87
0.16
KLHDC3-/- 293T K2D8
0.97
0.53
3.25
0.99
0.93
0.24
KLHDC3-/- 293T K2D10
Count
GFP/DsRed ratio Median(Derived)

 © The Author(s)

Figure EV4. Generation of KLHDC3$^{-/-}$ 293 T cells.

(**A**) Schematic and analysis of 3 independent *KLHDC3$^{-/-}$* 293 T cell lines. Each line was confirmed as a homozygous mutant. (**B**) The C-terminal region of the *Recql4* deleted allele coding for p.V359-E481GfsX48 was cloned into the GPS-reporter plasmid. In this plasmid DsRed is constitutively expressed and GFP stability/expression is determined by the C-terminus of the fused RECQL4 deleted product. This reporter was expressed in KLHDC3 WT 293 T cells or *KLHDC3$^{-/-}$* K2D10 293 T cells as indicated. Fluorescence signal was detected by live cell fluorescent imaging; images of RECQL4 p.V359-E481GfsX48 clone S5 and S6 (clone S4 shown in Fig. 5). Scale bar represents 50 µm. (**C**) Validation of KLHDC3 deficiency using GPS reporter assay by either live cell fluorescent microscopy of flow cytometry. Representative images of GPS reporters for USP49, PPP1R15A and NS3 (all KLHDC3 substrates) and NS3GA and NS8 (not KLHDC3 substrates) by live cell fluorescent imaging in WT and a *KLHDC3$^{-/-}$* 293 T cell (clone K2D10). Scale bar represents 50 µm. Representative flow cytometric analysis of the GFP and DsRed expression in 3 independently generated *KLHDC3$^{-/-}$* 293 T cells compared to KLHDC3 WT 293 T cells; Derived mean value for each sample as indicated calculated using FlowJo. (**D**) Re-expression of KLHDC3 in the *KLHDC3$^{-/-}$* 293 T cells leads to loss of GFP signal for the known KLHDC3 substrates USP49, PP1R15A and NS3. The baseline GFP/DsRed derived median is in red, the KLHDC3 over-expressing samples are in blue. Derived mean value for each sample as indicated calculated using FlowJo. Note the left shift in the WT KLHDC3 re-expressing *KLHDC3$^{-/-}$* 293 T cells indicative of destruction of the GFP-fusion protein.

© The Author(s)

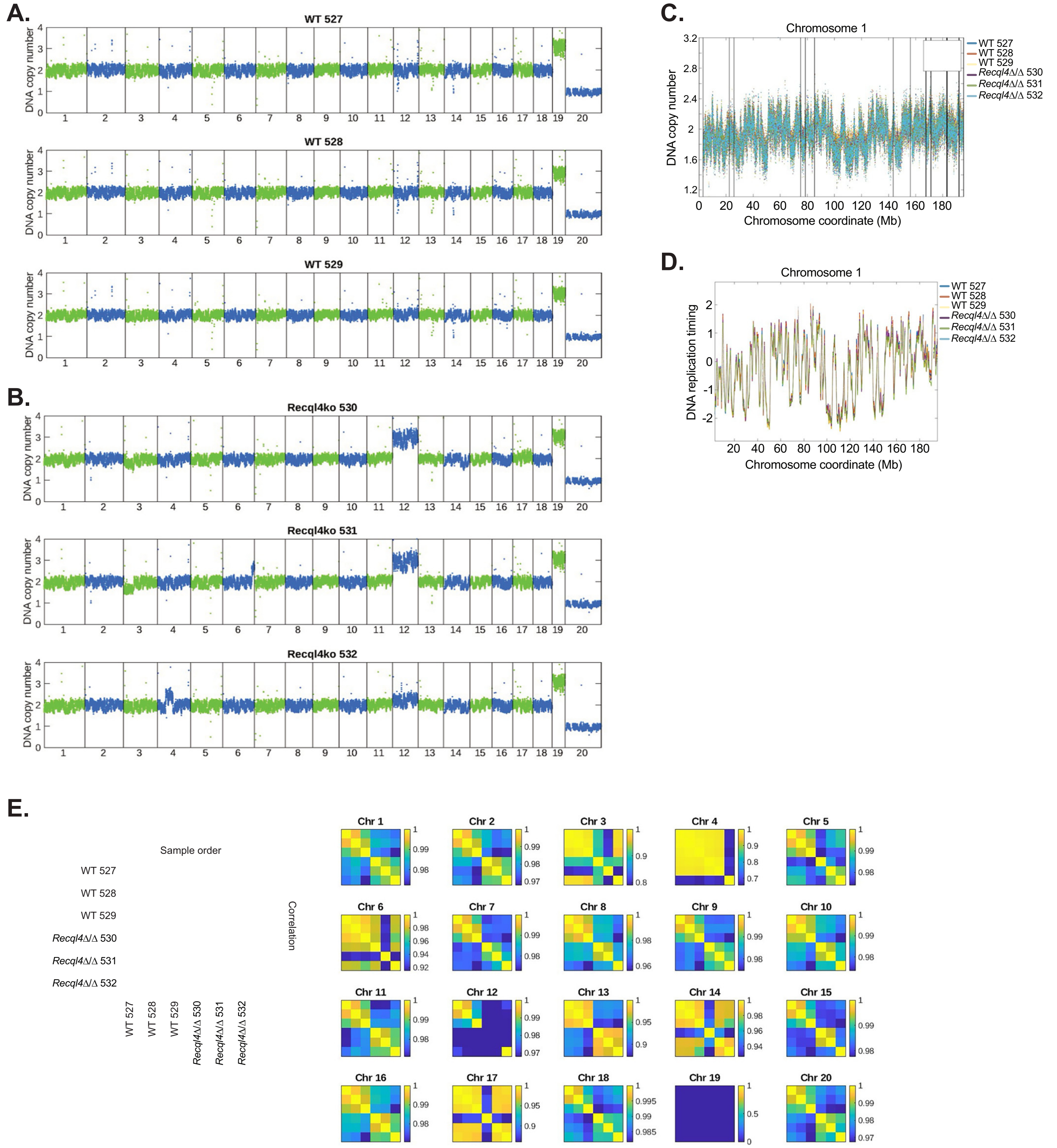

Figure EV5. Individual whole-genome DNA copy number analysis for the myeloid cell lines used for DNA replication timing inference.

(**A**) DNA copy number across all chromosomes for WT myeloid cell lines. (**B**) DNA copy number across all chromosomes for *Recql4*$^{\Delta/\Delta}$ sg*Klhdc3* myeloid cell lines. (**C**) Whole-genome sequencing was used to infer DNA replication timing across the genome in 3 control and 3 *Recql4*$^{\Delta/\Delta}$ sg*Klhdc3* cell lines (WT: 144 *Recql4* +/+ LCr. Hygro Ctrl C12, E12 and H11 and *Recql4* Δ/Δ: 185 *Recql4* Δ/Δ LCr. Hygro *sgKlhdc3* K2C12 and K1B12 and 186 *Recql4* Δ/Δ LCr. Hygro sg*Klhdc3* K1E1). Representative example from each sample is plotted individually for Chromosome 1. (**D**) Representative example of smoothed plots of DNA replication timing across chromosome 1 for each cell line. (**E**) Correlation analysis of DNA replication timing of each chromosome across the genome with the scale for each individual chromosome. Note that missing data, due to filtering of specific chromosomes in some samples, appears as dark blue in the correlation matrices.

 © The Author(s)
